# Ru‐Catalyzed Asymmetric Transfer Hydrogenation of α‐iminophosphonates: A Novel Synthetic Approach for Valuable Chiral α‐Aminophosphonates

**DOI:** 10.1002/chem.202502405

**Published:** 2025-09-09

**Authors:** Pierre Plouard, Laura Borrel, Pierre‐Olivier Butin, Paul Muller, Rémi Cherpitel, Séverine Loiseau, Ludovic Clarion, David Virieux, Tahar Ayad

**Affiliations:** ^1^ ICGM Univ Montpellier ENSCM CNRS Montpellier France; ^2^ Phost'in Therapeutics 104 rue de la galera 34090 Montpellier France

**Keywords:** asymmetric transfer hydrogenation, ruthenium catalyst, α‐Aminophosphonates, α‐Iminophosphonates

## Abstract

A novel Ru‐catalyzed asymmetric transfer hydrogenation (ATH) strategy has been developed for the efficient synthesis of valuable chiral *α*‐aminophosphonates from readily available *α*‐iminophosphonates. This method enables the conversion of both acyclic and cyclic substrates in high yields (up to 97%) and excellent enantioselectivities (up to >99:1), providing a practical entry to phosphorus‐containing chiral amines. Notably, this is the first reported application of ATH to *α*‐iminophosphonates, offering a robust and operationally convenient alternative to conventional asymmetric hydrogenation (AH) approaches.

## Graphical abstract

This study reports the first Ru‐catalyzed ATH of *α*‐iminophosphonates, enabling the efficient synthesis of chiral α‐aminophosphonates. Both acyclic and cyclic substrates are converted in high yields (up to 97%) with excellent enantioselectivities (up to >99:1), offering a practical alternative to conventional AH for accessing phosphorus‐containing chiral amines.

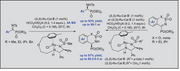

## 1 Introduction

**Figure 1:**
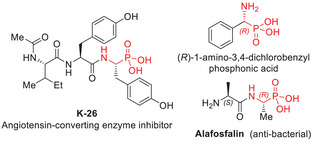
Representative bioactive molecules containing α‐amino‐phosphonic acid derivatives.

*α*‐aminophosphonic acid derivatives constitute a very appealing class of *α*‐amino acid analogs characterized by the substitution of the planar carboxylic acid group with a phosphonic acid group. This bioisosteric modification allows these compounds to stably and effectively mimic the transition state of peptide cleavage, making them potent inhibitors of proteolytic enzymes.^[^ ^1^ ^]^ A notable example is K‐26, a well‐known angiotensin‐converting enzyme inhibitor (Figure 1).^[^ ^2^ ^]^ Moreover, α‐aminophosphonic derivatives have attracted significant interest for their broad range of applications in pharmaceutical chemistry, demonstrating antimicrobial,^[^ ^3^ ^]^ anticancer^[^ ^4^ ^]^ and antiviral activities.^[^ ^5^ ^]^ They also play key roles in agrochemistry, particularly as herbicides.^[^ ^6^ ^]^ Given their varied biological activities, the importance of enantiomerically pure *α*‐aminophosphonic acid derivatives in medical applications is increasingly recognized, as their biological effects can vary significantly based on their absolute stereochemistry. For instance, among the four diastereomers of the antibiotic Alaphospholin, the (*S*,*R*) diastereomer exhibits the highest activity against pathogenic microorganisms, while the other three stereoisomers display much lower efficacy.^[^ ^7^ ^]^ Similarly, it has been shown that (*R*)‐1‐amino‐3,4‐dichlorobenzylphosphonic acid inhibits phenylalanine ammonia‐lyase nearly forty times more effectively than its (*S*)‐enantiomer. (Figure 1). As a result, significant efforts over the past few decades have focused on developing efficient methods for synthesizing single enantiomers of α‐aminophosphonic acids to fully unlock their therapeutic potential.^[^ ^8^ ^]^

**Scheme 1:**
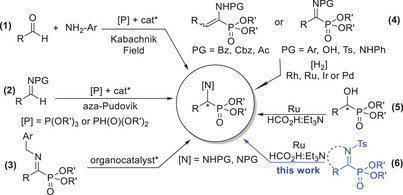
Reported preparation methods for chiral *α*‐iminophosphonates.

Among the various methods for synthesizing α‐aminophosphonates, two of the most widely used C─P bond forming approaches are the Kabachnik‐Fields condensation^[^ ^9^, ^10^ ^]^ and the aza‐Pudovik^[^ ^11^ ^]^ reaction (Scheme 1, methods 1 and 2). The Kabachnik‐Fields condensation, also known as the phospha‐Mannich reaction, involves a three‐component reaction between an oxo compound (such as an aldehyde or ketone) and a primary or a secondary amine in the presence of a P(O)H‐containing reagent. Typically, the reagent is a dialkyl phosphite, though alternatives like alkyl‐H‐phosphinates or secondary phosphine oxides can also be used. On the other hand, the Pudovik reaction involves the addition of a dialkyl phosphite to a preformed imine, providing a more direct route to α‐aminophosphonates. Although efficient, these established protocols often require high catalyst loadings (sometimes involving noncommercial chiral catalysts), cryogenic conditions, and extended reaction times to achieve satisfactory yields and selectivity. More recently, catalyst‐free and microwave‐assisted procedures have also been reported, offering greener and more sustainable alternatives.^[^ ^12^ ^]^ In 2015, Albrecht and coworkers introduced an elegant biomimetic strategy for synthesizing highly enantiomerically enriched α‐aminophosphonates (Scheme 1, method 4). This innovative approach relies on a base‐catalyzed isomerization of a double bond in the corresponding *N*‐benzylimines, followed by a hydrolytic deprotection step to reveal the amine functionality.^[^ ^13^ ^]^ While the method affords good to excellent enantiomeric excesses (up to 96%), the reaction yields are moderate (up to 63%) and are restricted to a limited range of alkyl‐substituted acylphosphonates. Transition‐metal‐catalyzed asymmetric hydrogenation (AH) of *α*‐phosphorylated enamines or imines offers an attractive and convenient alternative for the preparation of optically active *α*‐aminophosphonate derivatives (Scheme 1, method 5). Schöllkopf and coworkers disclosed in 1985, the first example of Rh‐catalyzed AH of *α,β*‐dehydroaminophosphonates, achieving moderate enantiomeric excesses up to 76% ee.^[^ ^14^ ^]^ Since this pioneering work, a considerable number of catalytic systems involving chiral ligands and transition metals, such as Ru, Rh, and Ir, are known to provide *α*‐aminophosphonate derivatives with enantioselectivities exceeding 95%.^[^ ^15^ ^]^ In sharp contrast, reports on the AH of α‐iminophosphonates remain scarce, a limitation attributed to the challenging synthesis of these substrates over the years. Notably, in 1994, Burk's group reported the first enantioselective synthesis of α‐aminophosphonates via Rh/DuPhos‐catalyzed hydrogenation of a hydrazone substrate, though only a single example was provided.^[^ ^15a^ ^]^ In 2008, Beletskaya and coworkers utilized a Rh/BINAP catalyst to reduce *α*‐iminophosphonates with a predominant E‐configuration of the imine bond, yielding α‐aminophosphonates with up to 82% yield and enantiomeric excesses ranging from 43% to 94%.^[^ ^16^ ^]^ Four years later, the same group extended this approach to the enantioselective reduction of *α*‐phosphorylated *α*‐aryl oximes using a Pd/BINAP complex and (1*S*)‐(+)‐10‐camphorsulfonic acid (CSA) as an activator, achieving yields up to 85% and enantiomeric excesses between 72% and 90%.^[^ ^17^ ^]^ Building on this strategy, the group developed a one‐pot, two‐step process for the synthesis of optically active, unprotected *α*‐aminophosphonates, with yields ranging from 52% to 82% and enantiomeric excesses between 90% and 98%, through Pd/(*R*)‐Cl─MeO‐BIPHEP catalyzed hydrogenation/ hydrogenolysis of *α*‐hydrazono phosphonates.^[^ ^18^ ^]^ In 2016, Zhou and coworkers reported an efficient Pd/(*R*)‐DifluorPhos‐catalyzed enantioselective reduction of *N*‐tosyl‐protected α‐iminophosphonates to α‐aminophosphonates, achieving isolated yields of 91–98% and enantiomeric purities ranging from 85% to 97%.^[^ ^19^ ^]^

Asymmetric transfer hydrogenation (ATH) using platinum‐group metal catalysts has emerged as a practical and versatile method for the enantioselective reduction of both carbonyl and imino compounds.^[^ ^20^ ^]^ This technique offers several advantages over traditional AH, including enhanced safety, operational simplicity, and the use of environmentally friendly, cost‐effective, and easy‐to‐handle hydrogen donors. By eliminating the need for hazardous hydrogen gas and high‐pressure reactors, ATH significantly improves accessibility and practicality. As far as ATH is concerned, the only reported application to the synthesis of α‐aminophosphonic acids was recently described by Pallitsch and coworkers.^[^ ^21^ ^]^ In this pioneering study, several enantiomerically pure α‐aminophosphonic acids were obtained via a multi‐step sequence, in which the ATH of oxo‐phosphonates played a central role, followed by a Mitsunobu reaction and subsequent hydrogenation. Although this method consistently delivered excellent enantiomeric excesses (97–99% ee), its broader applicability may be limited by the use of highly reactive and potentially hazardous reagents, such as hydrazoic acid and azodicarboxylates.

Despite significant advances in this field, the direct ATH of *α*‐iminophosphonate derivatives to yield *α*‐aminophosphonates remains unreported and continues to present a substantial challenge. Simultaneously to the work of Pallitsch and coworkers,^[^ ^21^ ^]^ we reported a highly enantioselective Ru‐catalyzed transfer hydrogenation method for readily accessible α‐ketophosphonates, yielding enantiomerically enriched *α*‐hydroxy phosphonates with consistently excellent isolated yields (up to 93%) and near‐perfect stereocontrol (>99% ee).^[^ ^22^ ^]^ Furthermore, Palacios and coworkers recently described a convenient synthetic route to N‐tosyl *α*‐ketiminophosphonate derivatives, which are structurally related to *α*‐ketophosphonates.^[^ ^23^ ^]^ Building on these developments, we investigated whether these derivatives could serve as suitable substrates for the synthesis of optically active α‐aminophosphonates via direct ATH (Scheme 1, method 6). In this work, we describe the first instance of ruthenium‐catalyzed direct ATH applied to both linear and cyclic *α*‐iminophosphonates, delivering *α*‐aminophosphonates in high to excellent isolated yields (up to 97%) and outstanding enantiomeric ratios (up to 99.5:0.5).

## 2 Results and Discussion

We began our study with the identification of the optimal reaction conditions, building on our previously optimized protocol for the ATH of *α*‐ketophosphonates.^[^ ^22^ ^]^ Using diisopropyl (phenyl(tosylimino)methyl)phosphonate **1a** as the model substrate, we employed 1 mol % of the Noyori − Ikaria Ru(II)−TsDPEN catalyst^[^ ^24^ ^]^ **A** with a 5:2 azeotropic mixture of formic acid and triethylamine as the hydrogen source in anhydrous CH_2_Cl_2_ at 25 °C for 8 hours (Table 1). This resulted in the isolation of desired product **2a** in a modest 46% yield with an enantiomeric ratio of 68:32 (entry 1). ^31^P NMR monitoring of the reaction clearly revealed that the low yield was due to the concomitant hydrolysis of the starting material by residual water, leading to the formation of the corresponding *α*‐ketophosphonate. This intermediate was subsequently reduced in situ to produce the *α*‐hydroxyphosphonate side‐product **2a’** in a 43% yield and with an excellent ee of 96%. To address this issue, we introduced drying agents and evaluated their impact on the formation of the α‐hydroxyphosphonate by‐product **2a’** (entries 1 − 3). Notably, the use of magnesium sulfate effectively suppressed hydrolysis, improving the yield of the desired product **2a** to 71%, while reducing the formation of **2a’** to 15% (entry 2). Moreover, the enantiomeric ratio of **2a** increased to 76:24 under these conditions. Further optimization using activated 4 Å molecular sieves was even more effective, almost completely suppressing the hydrolysis side pathway, with less than 5% of **2a’** being detected by ^31^P NMR analysis. As a result, the desired product **2a** was obtained in an excellent isolated yield of 87% with an enantiomeric ratio of 76:24 (entry 3). Next, a series of commercially available ruthenium catalysts bearing different substituents on the chiral diamine moieties was screened (Table 1, entries 4 − 7). Switching from a tosyl group (catalyst **A**) to an electron‐deficient pentafluorobenzenesulfonyl moiety (catalyst **B**) had almost no effect, as the product was isolated in a similar yield and selectivity (entries 3 and 4). In contrast, the use of tethered complex catalyst **C** developed by Wills^[^ ^25^ ^]^ resulted in a significant improvement in terms of both reactivity and selectivity, giving **2a** in 92% isolated yield and an 89:11 er (entry 5). The catalytic performance was further enhanced using Ikariya oxo‐tethered Ru(II) catalyst **D**,^[^ ^26^ ^]^ which furnished **2a** with a higher enantiomeric ratio of 96:4 (entry 6). Finally, replacing the tosyl substituent with a mesyl group (catalyst **E**) allowed us to reach an excellent er of 97:3 (entry 7). Given that metals other than ruthenium, such as Iridium and Rhodium, have been successfully employed in the ATH of imines, iso‐electronic catalysts **F** and **G**, generated in situ by mixing the dimers [Cp*MCl_2_]_2_ (M = Ir, Rh) with the TsDPEN chiral diamine ligand in a 1:2 ratio, were also tested. However, neither of these alternative catalysts provided better results. Specifically, the Ir‐based catalyst **F** exhibited no detectable hydrolysis, likely due to its higher intrinsic reactivity. However, it afforded the desired product **2a** with only moderate selectivity of 67:33 er (entry 8). In contrast, the Rh‐based catalyst **G** displayed sluggish reactivity, achieving only 94% conversion after an extended reaction time of 18 hours, accompanied by a high degree of hydrolysis. In both cases, the level of stereoinduction was comparable to that obtained with nontethered Ru catalysts **A** and **B** (entry 9).


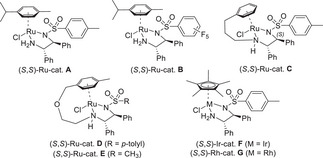


**Table 1:** Optimization of reaction conditions.^[^ ^a^ ^]^

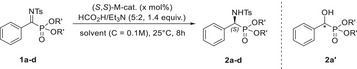					
				2a‐d	
Entry	M‐cat. (x mol%)	R’	Solvent	Yield (%)^[^ ^b^ ^]^	er (%)^[^ ^c^ ^],[^ ^d^ ^]^
1^[^ ^e^ ^]^	**A** (1 mol%)	*i*Pr, **1a**	CH_2_Cl_2_	46	68:32
2^[^ ^f^ ^]^	**A** (1 mol%)	*i*Pr, **1a**	CH_2_Cl_2_	71	76:24
3^[^ ^g^ ^]^	**A** (1 mol%)	*i*Pr, **1a**	CH_2_Cl_2_	87	76:24
4	**B** (1 mol%)	*i*Pr, **1a**	CH_2_Cl_2_	86	77:23
5	**C** (1 mol%)	*i*Pr, **1a**	CH_2_Cl_2_	92	89:11
6	**D** (1 mol%)	*i*Pr, **1a**	CH_2_Cl_2_	91	96:4
7	**E** (1 mol%)	*i*Pr, **1a**	CH_2_Cl_2_	88	97:3
8	**F** (1 mol%)	*i*Pr, **1a**	CH_2_Cl_2_	88	67:33
9^[^ ^h^ ^]^	**G** (1 mol%)	*i*Pr, **1a**	CH_2_Cl_2_	53	76:24
10	**E** (1 mol%)	*i*Pr, **1a**	Cl(CH_2_)_2_Cl	76	94:6
11	**E** (1 mol%)	*i*Pr, **1a**	THF	79	93:7
12	**E** (1 mol%)	*i*Pr, **1a**	2‐Me THF	81	93:7
13	**E** (1 mol%)	*i*Pr, **1a**	CH_3_CN	79	87:13
14	**E** (1 mol%)	Me,**1b**	CH_2_Cl_2_	71	98:2
15	**E** (1 mol%)	Et, **1c**	CH_2_Cl_2_	93	98:2 (>99:1)^[^ ^i^ ^]^
16	**E** (1 mol%)	Bn,**1d**	CH_2_Cl_2_	84	97:3
17	**E** (0.5 mol%)	Et, **1c**	CH_2_Cl_2_	82	97:3

^[a]^ Conditions:**1** (0.2 mmol), M‐cat. (x mol %), HCO_2_H/Et_3_N (5:2, 1.4 equiv), solvent (C = 0.1 M), rt, 8 hours.

^[b]^ Isolated yields of **2a**.

^[c]^ Determined by chiral HPLC analysis.

^[d]^ Absolute configurations of **2a − d** were determined to be *S* by comparison of the signs of the optical rotation values and the HPLC chromatograms previously reported in the literature.

^[e]^ With no additive, **2a’** was isolated in 36% yield.

^[f]^ Reaction run with 50 mg of MgSO4, 15% of **2a’**.

^[g]^ Reaction run with 50 mg of 4 Å MS, 5% of **2a’**.

^[h]^ 18 hours, 94% conv, 16% of ketone, and 4% of **3a**.

^[i]^ After recrystallization in CH_2_Cl_2_/*n*‐hexane.

**Figure 2:**
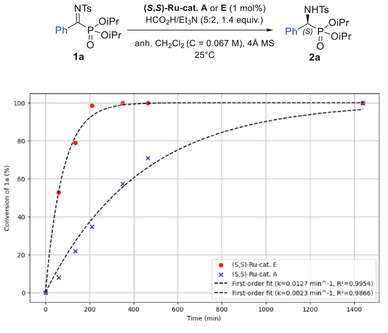
Comparative kinetic study of catalysts **A** and **E** for the ATH of **1a**.

From this initial screening, the tethered catalyst **E** emerged as the most efficient, which we attributed to its higher reactivity that allowed it to bypass the hydrolysis pathway caused by residual water present in the reaction mixture. To confirm this hypothesis, a comparative kinetic study was conducted, evaluating tethered catalyst **E** against the nontethered catalyst **A** using ^31^P NMR. As shown in Figure 2, the conversion over time followed a first‐order kinetic model, consistent with literature reports,^[^ ^27^ ^]^ allowing the calculation of the observed kinetic constant, k_obs_, for both systems. Catalyst **E** exhibited significantly higher activity, with a k_obs_ value 4.6 times greater than that of catalyst **A**, achieving full conversion within 350 minutes (5 hours 50 minutes). Notably, unlike catalyst **A**, the percentage of hydrolysis for catalyst **E** remained unchanged over time, indicating that under these conditions, the hydrogenation rate surpasses the rate of imine hydrolysis.

Having identified the optimal catalyst, we examined the influence of the solvent on the stereochemical outcome of the reaction. Replacing dichloromethane with dichloroethane led to the isolation of **2a** with a good, albeit slightly reduced, enantiomeric ratio of 94:6 (entry 10). Similar trends were observed with ethereal solvents such as THF and Me‐THF, both of which led to decreased selectivity (entries 11 and 12). Using acetonitrile was even more detrimental, lowering the selectivity to 87:13 er (entry 13). Overall, we investigated the influence of the alkyl group in dialkyl phosphonates. Replacing the isopropyl substituent with a methyl, ethyl, or benzyl group was found to have a negligible effect on selectivity, with enantiomeric ratios ranging from 97:3 to 98:2. However, the ethyl group demonstrated the highest efficiency in the present ATH, achieving an isolated yield of 93% (entries 14–16). Notably, the enantiomeric ratio of compound **2c** could be improved up to >99:1 by a single recrystallization from a mixture of dichloromethane and *n*‐hexane. Additionally, reducing the catalyst loading from 1 mol% to 0.5 mol% had little effect on the catalytic performance (entry 17).

Having established the optimal conditions, we explored the scope of the reaction using a series of diversely functionalized diethyl (aryl(tosylimino)methyl)phosphonates. Reactions were conducted with 1 mol% of the tethered catalyst **E** in an anhydrous CH_2_Cl_2_ at 25 °C for 8 hours, using a 5:2 azeotropic mixture of formic acid and triethylamine as the hydrogen source. As summarized in Table 2, the reaction proceeded efficiently across all substrates, delivering the corresponding enantioenriched *α*‐aminophosphonates (**2c**–**n**) with good to excellent isolated yields (81–93%) and enantiomeric ratios (up to >99:1) with no detectable traces of *α*‐hydroxyphosphonate side‐products.

**Table 2:** Substrate scope.^[^ ^a^ ^]^

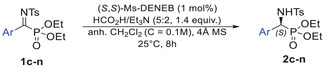				
Entry	2	Ar	Yield (%)^[^ ^b^ ^]^	er (%)^[^ ^[c]^, ^[d]^ ^]^
1	**2c**	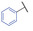	93	98:2 (>99:1)^[^ ^e^ ^]^
2	**2e**	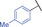	90	99:1
3	**2f**	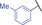	89	97:3
4	**2g**	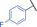	87	97:3
5	**2h**	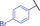	91	98:2
6	**2i**	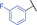	88	96:4
7	**2j**	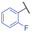	90	99:1
8	**2k**	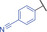	87	92:8
9	**2l**	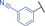	84	88:12
10	**2m**	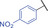	81	82:18
11	**2n**	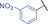	82	80:20

^[a]^ Conditions: **1** (0.2 mmol), (*S*,*S*)‐Ms‐DENEB (1 mol %), HCO_2_H/Et_3_N (5:2, 1.4 equiv), CH_2_Cl_2_ (C = 0.1 M), rt, 8 hours.

^[b]^ Isolated yields.

^[c]^ Determined by chiral HPLC analysis.

^[d]^ Absolute configurations of **2a − d** were determined to be S by comparison of the signs of the optical rotation values and the HPLC chromatograms obtained with those previously reported in the literature.

^[e]^ After recrystallization in CH_2_Cl_2_/*n*‐hexane.

The electronic nature and position of the substituent on the phenyl ring had a significant influence on the outcomes of the reactions. Substrates **1e** and **1f**, which have para‐ or meta‐methyl electron‐donating groups, produced **2e** and **2f** with very good isolated yields and excellent enantiomeric ratios (entries 2 and 4). Similarly, substrates with electron‐withdrawing groups such as ‐F or ‐Br, in the para or meta positions, produced comparable results in terms of yields and selectivities (entries 4–6). Notably, the ortho‐substituted substrate **1j** exhibited significantly improved enantioselectivity, yielding **2j** with an almost perfect enantiomeric ratio of 99:1 (entry 7). This suggests that steric effects play a key role in influencing selectivity. In contrast, as previously reported by Zhou and coworkers,^[^ ^19^ ^]^ substituents with strong electron‐withdrawing effects through mesomeric interactions significantly impacted enantioselectivity (entries 8–11). For instance, the para‐cyano derivative **1k** delivered a slightly reduced, yet still high, enantiomeric ratio of 92:8 (entry 8), while its meta‐substituted counterpart **1l** exhibited an even lower ratio of 87:13, suggesting that the position of the substituent also plays a role in stereochemical outcome (entry 9). This effect was even more pronounced with the highly electron‐withdrawing nitro group, as ortho‐ and meta‐substituted derivatives **1 m** and **1n** afforded even lower enantiomeric ratios of 82:18 and 80:20, respectively (entries 10 and 11). To further demonstrate the versatility of our method, we broadened the scope of the ATH to encompass a range of diversely substituted five‐ and six ‐membered cyclic *α*‐iminophosphonates. These compounds were readily synthesized from commercially available salicylaldehyde derivatives and *N*‐tert‐butylbenzenesulfonamide following a modified procedure reported in the literature by Zhou and coworkers.^[^ ^19^ ^]^ Given that aryl substituents within the molecular framework can enhance rigidity and influence electronic properties, we decided to re‐optimize the reaction conditions, with a focus on the influence of the catalysts, using compound **3a** as a model substrate (see Supporting Information for details). Additionally, due to the enhanced hydrolytic stability of the cyclic *α*‐iminophosphonates, the reactions were carried out without the use of molecular sieves. As shown in table 3, and in contrast to the results observed with acyclic substrates, the Ru(II) (*S*,*S*)‐Ts‐DENEB catalyst **D** exhibited significantly superior catalytic performance compared to the Ru(II) (*S*,*S*)‐Ms‐DENEB **E** when applied to the cyclic *α*‐iminophosphonate **3a** bearing an isopropyl substituent. As anticipated, no detectable hydrolysis by‐product was observed over the course of the ATH reaction. Under the optimized conditions, the desired *α*‐aminophosphonate **4a** was obtained with an excellent isolated yield of 97% (vs. 91%) and an outstanding enantiomeric ratio of >99:1 (vs. 93:7). This result may be attributed to the enhanced stabilizing *π*‐*π* interactions between the tosyl group of the catalyst and the phenyl moiety of the substrate. Remarkably, high catalytic activity was maintained even at a reduced catalyst loading of 0.1 mol%, affording **4a** with an enantiomeric ratio of 97:3, albeit with a slightly lower yield of 88% and an extended reaction time of 72 hours. Interestingly, the substrate **4b** bearing an ethyl phosphonate group gave a similar enantiomeric ratio to its isopropyl counterpart, albeit with a slightly lower isolated yield of 92%. To assess the impact of steric and electronic effects, a series of derivatives (**3c–f**) were prepared by introducing substituents onto the phenyl ring. A methyl group at the 7‐position preserved the excellent catalytic performance (96% yield, >99:1 er), while a methoxy group led to consistently lower enantioselectivities (up to 96:4 er) and yields (up to 90%), regardless of its position on the phenyl ring. Furthermore, this methodology was also effective for five‐membered cyclic *α*‐iminophosphonates, which underwent complete conversion to the corresponding *α*‐aminophosphonates under the optimized conditions using the (*S*,*S*)‐Ms‐DENEB catalyst instead of the (*S*,*S*)‐Ts‐DENEB catalyst (see Supporting Information for catalyst screening). Interestingly, the introducing of a methyl group at the 5‐position of the phenyl ring enhanced the overall reactivity without compromising selectivity, while the incorporation of an electron‐withdrawing fluorine atom at the same position adversely affected both the isolated yield and selectivity.

**Table 3:** Substrate scope: cyclic *α*‐iminophosphonates^[^ ^a^ ^]^

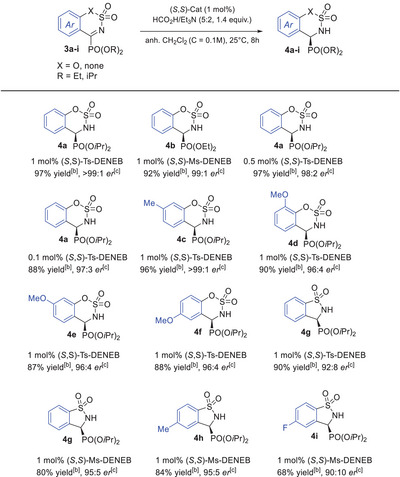

^[a]^ Conditions:**3** (0.2 mmol), (*S*,*S*)‐Cat (1 mol %), HCO_2_H/Et_3_N (5:2, 1.4 equiv), CH_2_Cl_2_ (C = 0.1 M), rt, 8 hours.

^[b]^ Isolated yields.

^[c]^ Determined by chiral HPLC analysis. Absolute configurations of **4a − e** were determined to be S by comparison of the signs of the optical rotation values and the HPLC chromatograms obtained with those previously reported in the literature.

## 3 Conclusion

In summary, we have developed a novel Ru‐catalyzed ATH methodology for the efficient synthesis of chiral *α*‐aminophosphonates from readily accessible *α*‐iminophosphonates. Under optimized conditions, a broad range of acyclic aryl α‐iminophosphonates were smoothly converted to the corresponding *α*‐aminophosphonates in good to excellent yields (up to 93%) and with high enantiomeric ratios (up to 99:1). This strategy was also successfully extended to five‐ and six‐membered cyclic *α*‐iminophosphonates, delivering the corresponding cyclic products with outstanding enantioselectivities (up to >99:1) and isolated yields reaching 97%. To the best of our knowledge, this represents the first reported application of ATH to *α*‐iminophosphonates, offering a practical and versatile alternative to traditional AH. Ongoing work in our laboratory aims to expand the substrate scope to include more challenging and functionally diverse derivatives, such as alkyl‐substituted analogues, further demonstrating the synthetic potential of this methodology.

## 4 Experimental section

### General informations

All reactions were run under an atmosphere of nitrogen using standard Schlenk techniques otherwise stated. Liquid aldehydes were distilled under reduced pressure before use. Reaction vessels were flame‐dried under vacuum and cooled under a stream of nitrogen. Solvents were carefully dried by conventional methods or were purified with an MBRAUN Solvent Purification System and degassed prior to use. Reactions were monitored by thin layer chromatography (TLC) on silica gel precoated plastic sheets (0.2 mm, Machery‐Nagel). Visualization of the developed chromatogram was performed by UV light and revealed using either potassium permanganate or phosphomolybdic acid solutions. Flash column chromatography (FC) was performed on Merck silica gel (60, particle size 0.040‐0.063 mm).

### General procedure for Asymmetric Transfer Hydrogenation of 1e‐n

In a microwave tube containing activated molecular sieves (approximately 20 beads) under an argon atmosphere, α‐iminophosphonates (0.2 mmol) and (*S*,*S*)‐Ms‐DENEB catalyst (1.1 mg, 1 mol%) are dissolved in anhydrous CH_2_Cl_2_ (2 mL). The setup undergoes three cycles of vacuum and argon backfill to ensure an inert environment. Subsequently, HCO_2_H:Et_3_N (5:2, 0.024 mL, 1.4 equiv.) is added dropwise, and the reaction mixture is stirred at room temperature for 8 hours. Upon completion as monitored by ^31^P NMR, the reaction mixture was concentrated *under vacuum* and purified by column chromatography on silica gel (AcOEt/petroleum ether).

### General procedure for Asymmetric Transfer Hydrogenation of 3a‐f

In a sealed tube under an argon atmosphere, α‐iminophosphonate (0.2 mmol) and (*S*,*S*)‐Ts‐DENEB catalyst (1.3 mg, 1 mol%) are dissolved in anhydrous CH_2_Cl_2_ (2 mL). The setup undergoes three cycles of vacuum and argon backfill to ensure an inert environment. Subsequently, HCO_2_H:Et_3_N (5:2, 0.024 mL, 1.4 equiv.) is added dropwise, and the reaction mixture is stirred at room temperature for 16 hours. Upon completion as monitored by ^31^P NMR, the reaction mixture was concentrated *under vacuum* and purified by column chromatography on silica gel (AcOEt/petroleum ether).

### General procedure for Asymmetric Transfer Hydrogenation of 3g‐i

In a microwave tube under an argon atmosphere, α‐iminophosphonates (0.2 mmol) and (S,S)‐Ms‐DENEB catalyst (1.1 mg, 1 mol%), are dissolved in anhydrous CH_2_Cl_2_ (2 mL). The setup undergoes three cycles of vacuum and argon backfill to ensure an inert environment. Subsequently, HCO_2_H:Et_3_N (5:2, 0.024 mL, 1.4 equiv.) is added dropwise, and the reaction mixture is stirred at room temperature for 16 hours. Upon completion as monitored by ^31^P NMR, the reaction mixture was concentrated *under vacuum* and purified by column chromatography on silica gel (AcOEt/petroleum ether).

## Supporting Information

Experimental procedures, characterization data, and copies of NMR spectra and HPLC data of all the compounds.

## Conflict of Interest

The authors declare no conflict of interest.

## Supporting information



Supporting Information

## Data Availability

The data that support the findings of this study are available in the supplementary material of this article.
