# Supplementary material for: Ru‐Catalyzed Asymmetric Transfer Hydrogenation of α‐iminophosphonates: A Novel Synthetic Approach for Valuable Chiral α‐Aminophosphonates
Source: Chemistry. 2025 Sep 9;31(55):e02405. doi: 10.1002/chem.202502405 (PMC12498074; doi:10.1002/chem.202502405)
Supplement: Supplementary file 1 — Supporting Information [file CHEM-31-e02405-s001.pdf]

SUPPORTING INFORMATION
Ru-Catalyzed Asymmetric Transfer Hydrogenation of α-iminophosphonates: A Novel Synthetic Approach for Valuable Chiral α-Aminophosphonates

Pierre Plouard,[a] Laura Borrel,[a] Pierre-Olivier Butin,[a] Paul Muller,[a] Rémi Cherpitel,[a] Séverine Loiseau[b] Ludovic Clarion[b] David Virieux*[a] and Tahar Ayad*[a]

[a] ICGM Univ Montpellier, CNRS, ENSCM, Montpellier, France
[b] Phost'in Therapeutics, 104 rue de la galera, 34090 Montpellier (France)
* E-mail: david.virieux@enscm.fr, tahar.ayad@enscm.fr

Table of contents

1. General informations 1
2. General procedure for synthesis of linear aryl α-iminophosphonates 2
3. Procedure for kinetic study of catalyst (S,S)-Ru-cat. **A** and **E**... 2
4. General procedure for Asymetric Transfer Hydrogenation of **1e-n** 6
5. General procedure for ATH of six-membered cyclic α-iminophosphonates **4a-f** 11
6. General procedure for ATH of five-membered cyclic α-iminophosphonates **4g-i** 14
7. ^{1}H, $^{31}P\{^{1}H\}$, ^{19}F and $^{13}C\{^{1}H\}$ NMR spectra of **2a-n** and **4a-i**... 17
8. HPLC chromatogram of **2a-n** and **4a-i** 49

1. General informations

All reactions were run under an atmosphere of nitrogen using standard Schlenk techniques otherwise stated. Liquid aldehydes were distilled under reduced pressure before use. Reaction vessels were flame-dried under vacuum and cooled under a stream of nitrogen. Solvents were carefully dried by conventional methods or were purified with an MBRAUN Solvent Purification System and degassed prior to use. Reactions were monitored by thin layer chromatography (TLC) on silica gel pre-coated plastic sheets (0.2 mm, Machery-Nagel). Visualization of the developed chromatogram was performed by UV light and revealed using either potassium permanganate or phosphomolybdic acid solutions. Flash column chromatography (FC) was performed on Merck silica gel (60, particle size 0.040-0.063 mm). ^{1}H, ^{13}C, ^{19}F and ^{31}P NMR spectra were recorded with a bruker Avance 400 MHz spectrometer. Chemical shifts are reported in delta (δ) units, part per million (ppm) downfield from tetramethylsilane (TMS) relative to the residual deuterated solvent peaks. Coupling constants are reported in Hertz (Hz). The following abbreviations are used: s = singlet, d = doublet, t = triplet, q = quartet, m = multiplet, br = broad signal. Enantiomeric ratios were determined by HPLC analysis measured on a Shimadzu®LC 20 AHPLC with a UV/visible detector using a chiral stationary phase column specified in the individual

experiment, by comparing the samples with the appropriate racemic mixtures. Optical rotations were measured on a polarimeter at 589 nm (sodium lamp) and 25°C (Peltier control) with a Bellingham + Stanley®ADP 450 Polarimeter. High resolution mass spectroscopic (HRMS) analysis were measured on a Xevo G2 Q TOF spectrometer using the electrospray method by the Laboratoire de Mesures Physiques of the University of Montpellier

2. General procedure for synthesis of linear aryl α-iminophosphonates

Linear aryl α-iminophosphonates substrates were synthesized in three steps from N-tosylaldimines[1] according to Palacios's procedure[2].

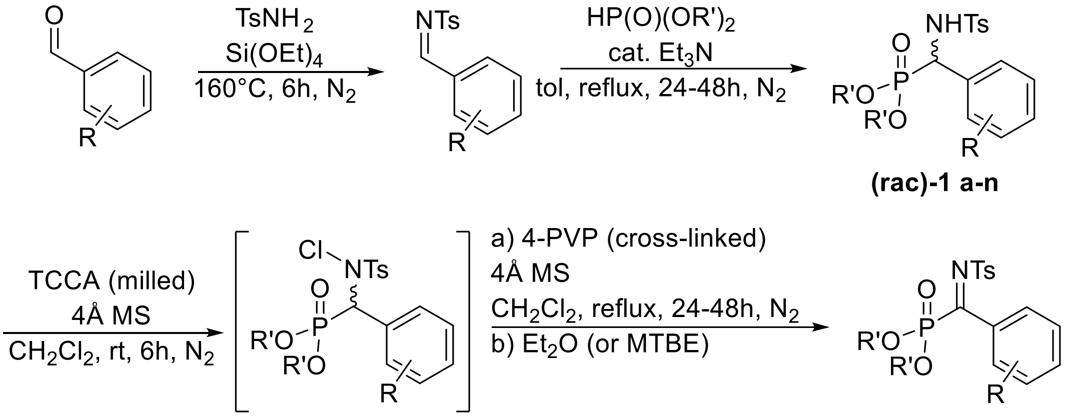

(rac)-1 a-n

2a-n

3. Procedure for kinetic study of catalyst (S,S)-Ru-cat. A and E

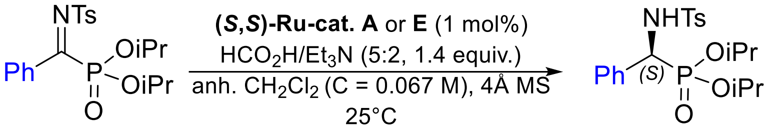

1a **2a**

In a microwave tube containing activated molecular sieves (approximately 20 beads) under an argon atmosphere, diisopropyl (phenyl(tosylimino)methyl)phosphonate (84.7mg, 0.2 mmol) and Ru-cat (**A** or **E**) (1.15 mg, 1 mol%) are dissolved in anhydrous CH_2Cl_2 (3 mL). The setup undergoes three cycles of vacuum and argon backfill to ensure an inert environment. Subsequently, HCO_2H:Et_3N (5:2, 0.024 mL, 1.4 equiv.) is added dropwise, and the reaction mixture is stirred at room temperature. After each given time intervals, an aliquot (0.5mL) is sampled and analyzed by $^{31}P\{^1H\}$ NMR using a DSMO-d_6 probe to determine the ratio of compounds **1a**, **2a** and **2a'.**

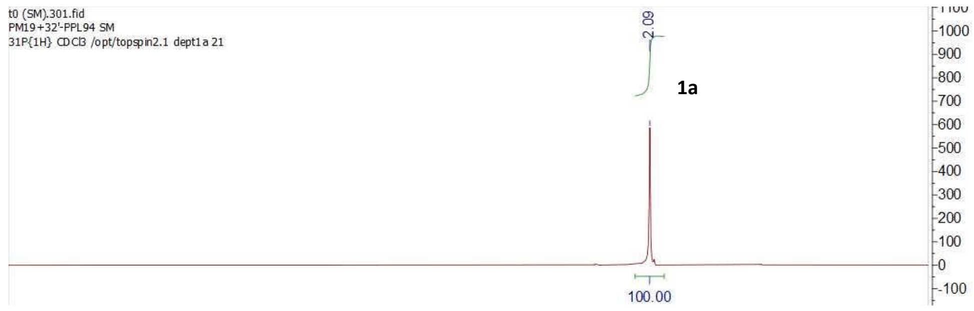

---

[1] Love, B. E., Raje, P. S., & Williams II, T. C. . *Synlett*, ***1994***(07), 493-494

[2] Vicario, J.; Ortiz, P.; Ezpeleta, J. M.; Palacios, F. *J. Org. Chem.* **2015**, 80, 156.

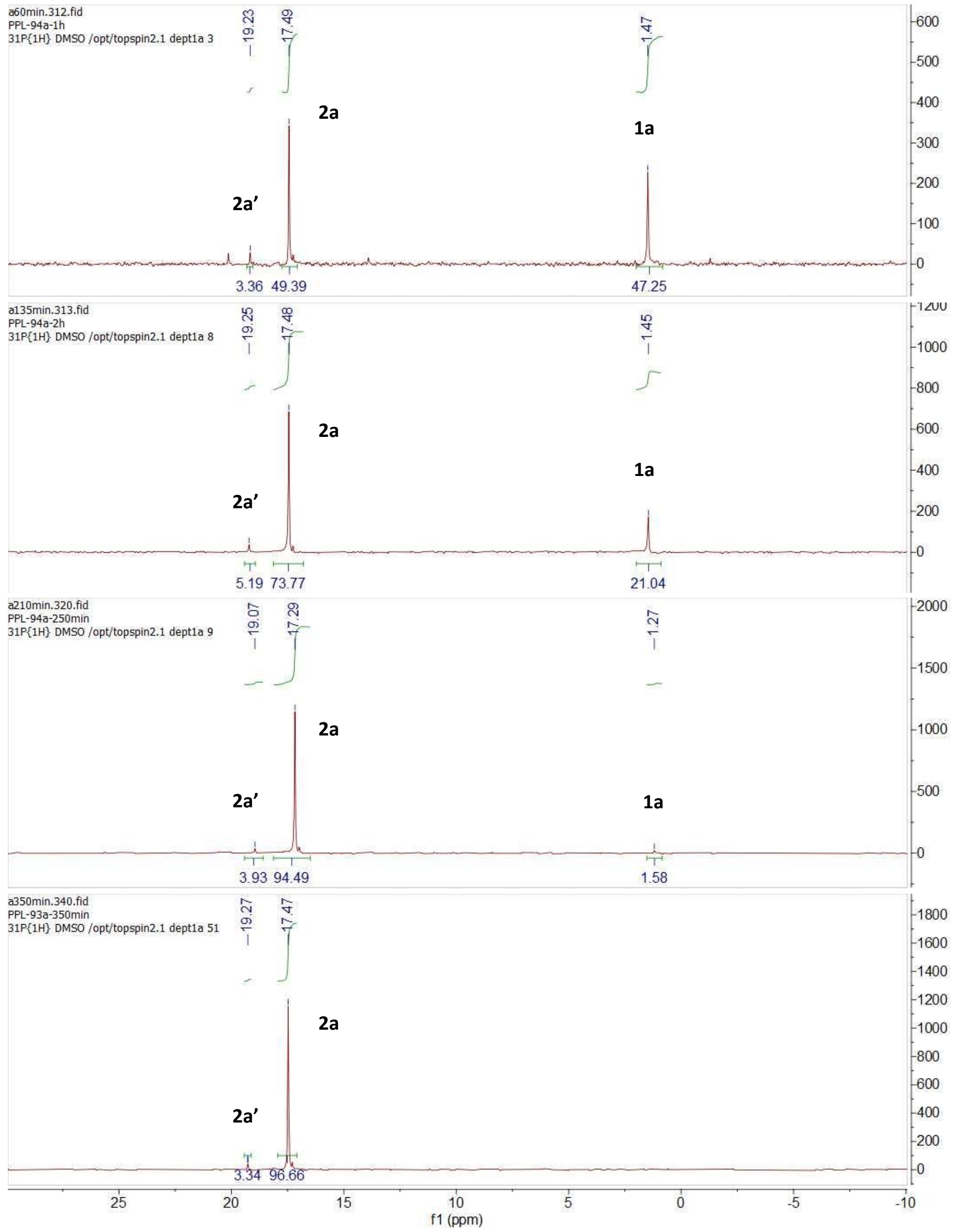

Figure S1. $^{31}P\{^{1}H\}$ NMR kinetic monitoring of ATH of **1a** with ***(S,S)*-Ru-cat. E**

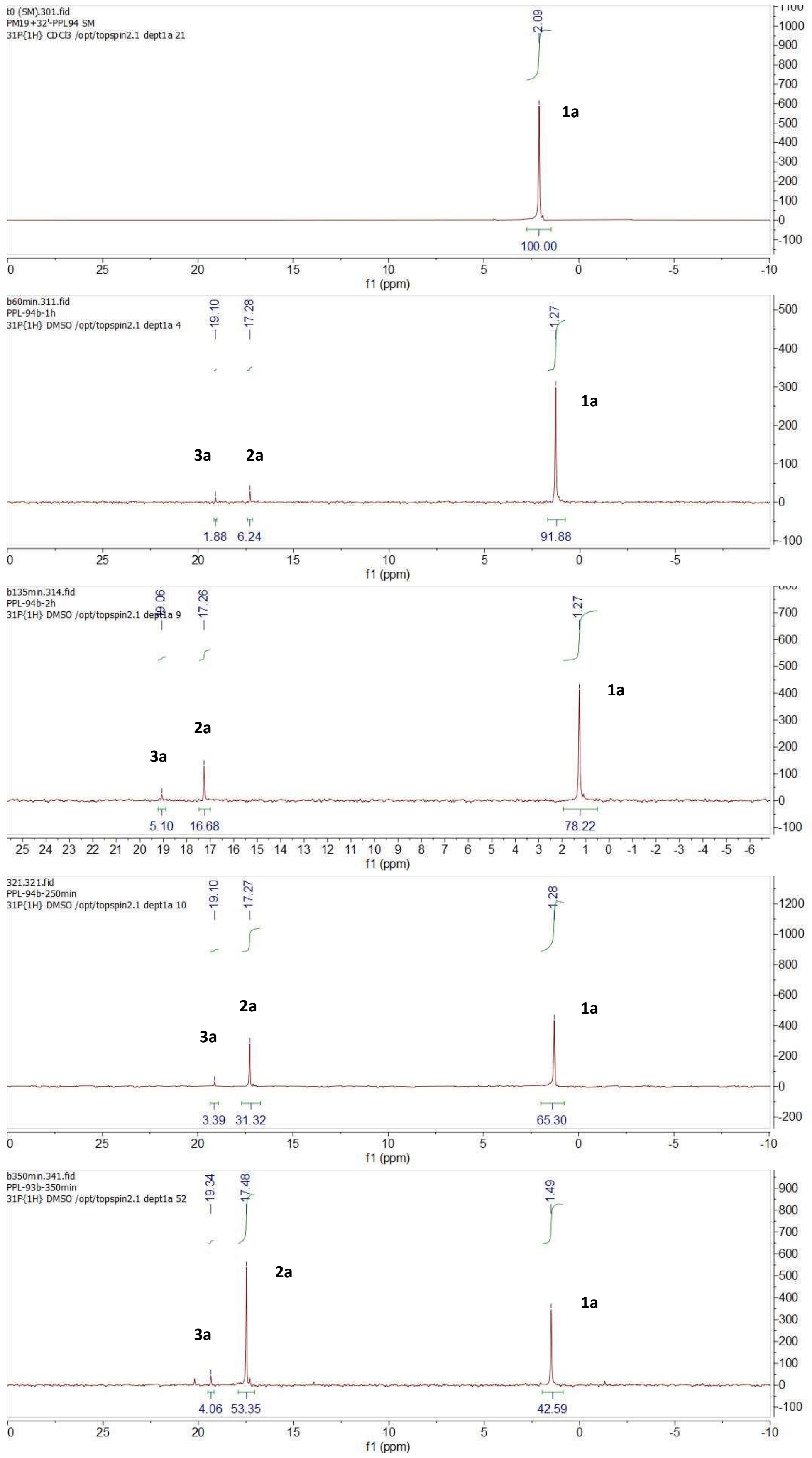

t0 (SM).301.fid
PM19+32'-PPL94 SM
31P{1H} CDCl3 /opt/topspin2.1 dept1a 21
2.09
1a
100.00
f1 (ppm)
b60min.311.fid
PPL-94b-1h
31P{1H} DMSO /opt/topspin2.1 dept1a 4
19.10
17.28
1.27
1a
3a
2a
1.88
6.24
91.88
f1 (ppm)
b135min.314.fid
PPL-94b-2h
31P{1H} DMSO /opt/topspin2.1 dept1a 9
19.06
17.26
1.27
1a
2a
3a
5.10
16.68
78.22
f1 (ppm)
321.321.fid
PPL-94b-250min
31P{1H} DMSO /opt/topspin2.1 dept1a 10
19.10
17.27
1.28
2a
1a
3a
3.39
31.32
65.30
f1 (ppm)
b350min.341.fid
PPL-93b-350min
31P{1H} DMSO /opt/topspin2.1 dept1a 52
19.34
17.48
1.49
2a
1a
3a
4.06
53.35
42.59
f1 (ppm)

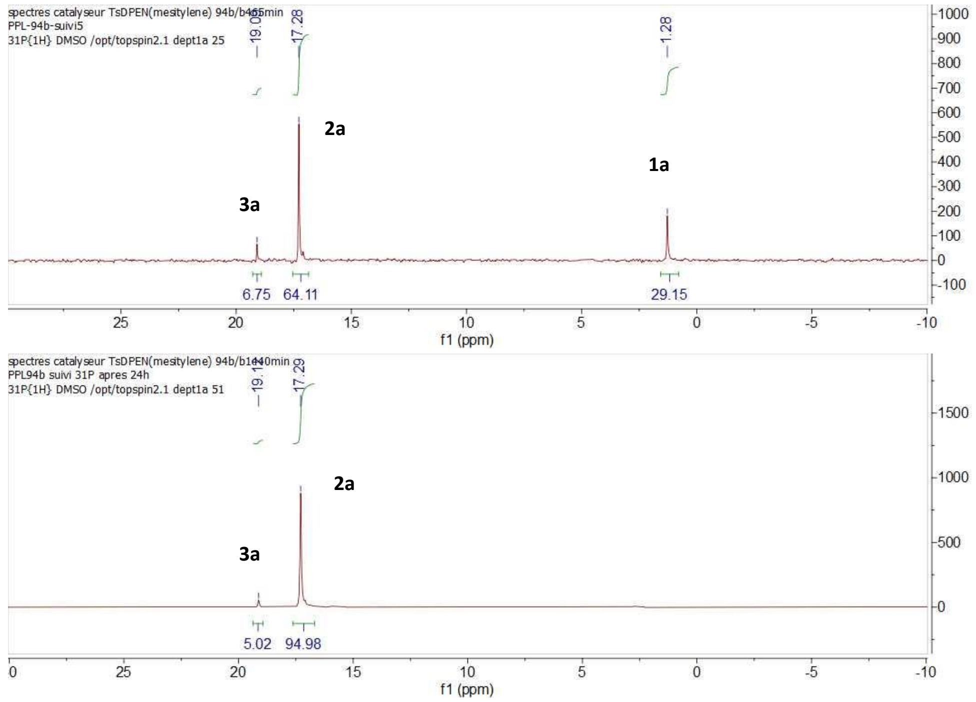

*Figure S2. $^{31}P\{^{1}H\}$ NMR kinetic monitoring of ATH of **1a** with **(S,S)-Ru-cat. A***

	Conversion of **1a** (%) (=100-%**1a**)	
t(min)	**(*S*,*S*)-Ru-cat. E**	**(*S*,*S*)-Ru-cat. A**
0	0	0
60	52,8	8,1
135	79	21,8
210	98,4	34,7
350	100	57,4
465	100	70,9
1440	100	100

*Table S1. Conversion of **1a** (%) over time in the ATH reaction catalyzed by **Ru-cat A** or **E***

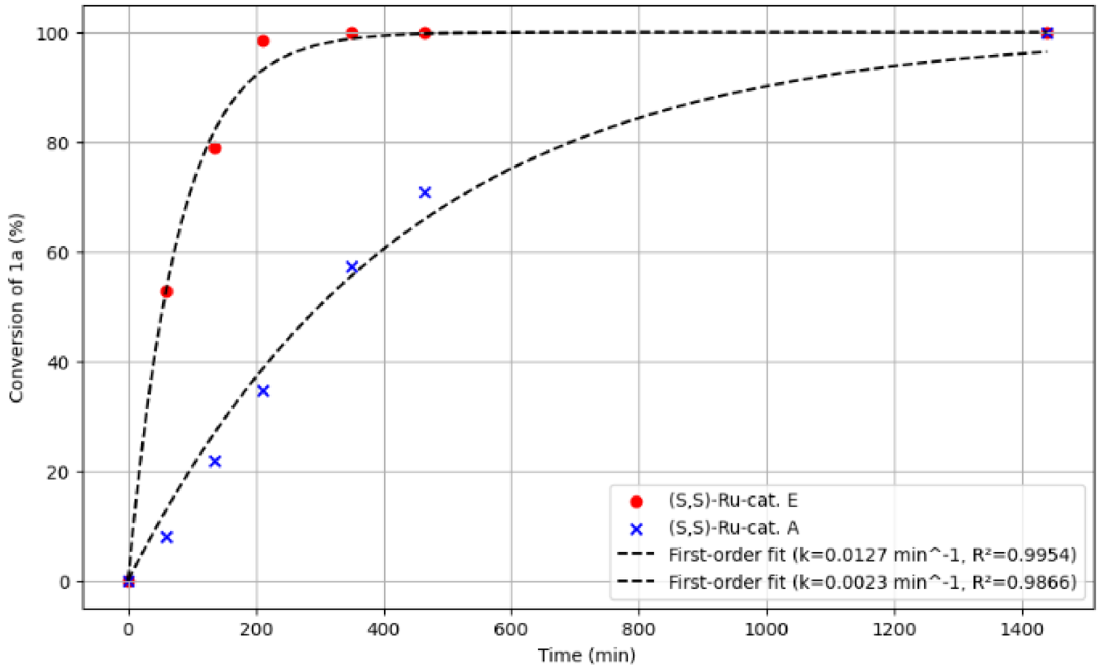

*Figure S3. Conversion of **1a** (**Ru-cat A** or **E** catalyzed ATH) versus time and first order fitting curves*

4. General procedure for Asymmetric Transfer Hydrogenation of 1e-n

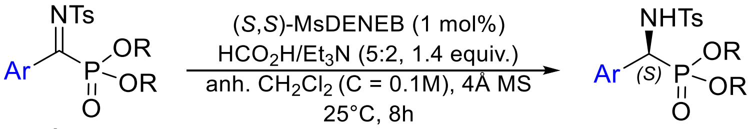

1a-n **2a-n**

In a microwave tube containing activated molecular sieves (approximately 20 beads) under an argon atmosphere, α-iminophosphonates (0.2 mmol) and (*S,S*)-Ms-DENEB catalyst (1.1 mg, 1 mol%) are dissolved in anhydrous CH_2Cl_2 (2 mL). The setup undergoes three cycles of vacuum and argon backfill to ensure an inert environment. Subsequently, HCO_2H:Et_3N (5:2, 0.024 mL, 1.4 equiv.) is added dropwise, and the reaction mixture is stirred at room temperature for 8 hours. Upon completion as monitored by ^{31}P NMR, the reaction mixture was concentrated *under vacuum* and purified by column chromatography on silica gel (AcOEt/petroleum ether).

Diisopropyl (S)-(((4-methylphenyl)sulfonamido)(phenyl)methyl)phosphonate (2a) (known compound[3]):

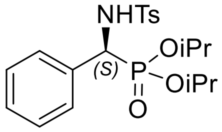

2a was obtained starting from diisopropyl (phenyl(tosylimino)methyl)phosphonate **1a** (84.7 mg, 0.2 mmol). Yield= 88%, er= 97:3 (94% ee), $[\alpha]^{25}_D$ = -9.2 (*c* 0.87, $CHCl_3$) [Lit. $[\alpha]^{20}_D$ = -9.75 (*c* 0.80, $CHCl_3$) for 96% ee (*S*)]. HPLC analysis: Chiralpak IA column, *n*-hexane/i-PrOH 65:35, flow = 0.7 mL/min, λ = 235 nm, t_R = 13.6 min (*R*, minor), t_R = 24.2 min (*S*, major).

$^{31}P\{^1H\}$ NMR (162 MHz, $CDCl_3$) δ 18.0. ^{1}H NMR (400 MHz, $CDCl_3$) δ 7.47 – 7.37 (m, 2H), 7.21 – 7.13 (m, 2H), 7.13 – 7.00 (m, 3H), 6.96 – 6.88 (m, 2H), 6.74 (dd, *J* = 9.2, 5.3 Hz, 1H), 4.91 – 4.77 (m, 1H), 4.72 (dd, *J* = 24.4, 9.6 Hz, 1H), 4.42 – 4.25 (m, 1H), 2.25 (s, 3H), 1.40 – 1.34 (m, 6H), 1.20 (d, *J* = 6.2 Hz, 3H),

[3] *Organic letters, 2016*, *18*(4), 692-695

0.76 (d, *J* = 6.2 Hz, 3H). $^{13}C\{^1H\}$ NMR (101 MHz, $CDCl_3$) δ 142.8, 138.0 (d, *J* = 1.8 Hz), 134.0, 129.0, 128.5 (d, *J* = 5.9 Hz), 128.1 (d, *J* = 2.2 Hz), 127.7 (d, *J* = 2.9 Hz), 127.2, 72.9 (d, *J* = 7.3 Hz), 72.7 (d, *J* = 7.3 Hz), 55.9 (d, *J* = 157.7 Hz), 24.4 (d, *J* = 3.2 Hz), 24.3 (d, *J* = 3.1 Hz), 23.9 (d, *J* = 5.6 Hz), 23.0 (d, *J* = 6.0 Hz).
Chemical Formula: C20H28NO5PS / Exact Mass: 425,1426 / Internal reference PPL-66/70-rac, PM-21F

Dimethyl (S)-(((4-methylphenyl)sulfonamido)(phenyl)methyl)phosphonate (2b) (known compound Erreur ! Signet non défini.**):**

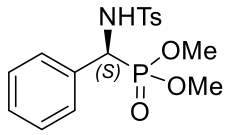

2b was obtained starting from dimethyl(phenyl(tosylimino)methyl)phosphonate **1b** (73.5 mg, 0.2 mmol).
Yield= 71%, er= 98.5:1.5 (97% ee), $[\alpha]^{25}_D$ = -22.2 (*c* 0.9, $CHCl_3$) [Lit. $[\alpha]^{20}_D$ = -26 (*c* 0.6, $CHCl_3$) for 94% ee (*S*)]. HPLC analysis: Chiralpak IA column, *n*-hexane/i-PrOH 65:35, flow = 0.7 mL/min, λ = 235 nm, t_R = 15.2 min (*R*, minor), t_R = 20.7 min (*S*, major).
$^{31}P\{^1H\}$ NMR (162 MHz, $CDCl_3$) δ 21.9. ^1H NMR (400 MHz, $CDCl_3$) δ 7.47 (d, *J* = 8.3 Hz, 2H), 7.23 – 7.05 (m, 5H), 6.99 (d, *J* = 7.9 Hz, 2H), 6.55 (s, 1H), 4.81 (d, *J* = 24.5 Hz, 1H), 3.83 (d, *J* = 10.8 Hz, 3H), 3.40 (d, *J* = 10.6 Hz, 3H), 2.28 (s, 3H). $^{13}C\{^1H\}$ NMR (101 MHz, $CDCl_3$) δ 143.1, 137.8 (d, *J* = 1.6 Hz), 133.5, 129.2, 128.5 (d, *J* = 2.2 Hz), 128.2 (d, *J* = 5.9 Hz), 128.1 (d, *J* = 2.9 Hz), 127.2, 54.9 (d, *J* = 155.9 Hz), 54.6 (d, *J* = 7.1 Hz), 54.2, 54.1 (d, *J* = 6.9 Hz), 21.5.
Chemical Formula: C16H20NO5PS / Exact Mass: 369,0800 / Internal reference PM-33A, PM-009 (rac)

Diethyl (S)-(((4-methylphenyl)sulfonamido)(phenyl)methyl)phosphonate (2c) (known compound Erreur ! Signet non défini.**):**

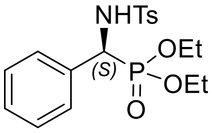

2c was obtained starting from diethyl(phenyl(tosylimino)methyl)phosphonate **1c** (79.1 mg, 0.2 mmol).
Yield= 93%, er= 98.5:1.5 (97% ee), $[\alpha]^{25}_D$ = -13.8 (*c* 0.87, $CHCl_3$) [Lit. $[\alpha]^{20}_D$ = -16.22 (*c* 0.74, $CHCl_3$) for 96% ee (*S*)]. HPLC analysis: Chiralpak IA column, *n*-hexane/i-PrOH 65:35, flow = 0.7 mL/min, λ = 235 nm, t_R = 14.5 min (*R*, minor), t_R = 25.4 min (*S*, major). $^{31}P\{^1H\}$ NMR (162 MHz, $CDCl_3$) δ 19.6. ^1H NMR (400 MHz, $CDCl_3$) δ 7.53 – 7.39 (m, 2H), 7.22 – 7.06 (m, 5H), 7.04 – 6.92 (m, 2H), 6.23 (dd, *J* = 8.9, 5.8 Hz, 1H), 4.75 (dd, *J* = 23.7, 9.3 Hz, 1H), 4.28 – 4.08 (m, 2H), 3.92 – 3.77 (m, 1H), 3.67 – 3.51 (m, 1H), 2.28 (s, 3H), 1.32 (t, *J* = 7.0 Hz, 3H), 1.02 (t, *J* = 7.0 Hz, 3H). $^{13}C\{^1H\}$ NMR (100 MHz, $CDCl_3$) δ 142.7, 138.2 (d, *J* = 1.8 Hz), 133.8, 129.0, 128.5 (d, *J* = 5.9 Hz), 128.2 (d, *J* = 2.2 Hz), 127.8 (d, *J* = 2.9 Hz), 127.2, 64.3 (d, *J* = 7.0 Hz), 63.7 (d, *J* = 7.0 Hz), 55.6 (d, *J* = 157.2 Hz), 21.4, 16.6 (d, *J* = 6.0 Hz), 16.2 (d, *J* = 5.6 Hz).
Chemical Formula: C18H24NO5PS / Exact Mass: 397,1113 / Internal reference PM-33B, PM-17 (rac)

Dibenzyl (S)-(((4-methylphenyl)sulfonamido)(phenyl)methyl)phosphonate (2d) (known compopund Erreur ! Signet non défini.**):**

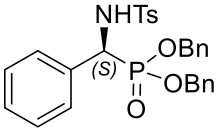

2d was obtained starting from dibenzyl(phenyl(tosylimino)methyl)phosphonate **1d** (103.9 mg, 0.2 mmol).
Yield= 84%, er= 97.5:2.5 (95% ee), $[\alpha]^{25}_D$ = +16.2 (*c* 0.91, $CHCl_3$) [Lit. $[\alpha]^{20}_D$ = +20.12 (*c* 0.84, $CHCl_3$) for 95% ee (*S*)]. HPLC analysis: Chiralpak IA column, *n*-hexane/i-PrOH 65:35, flow = 0.7 mL/min, λ = 235 nm, t_R = 28.4 min (*R*, minor), t_R = 36 min (*S*, major).
$^{31}P\{^1H\}$ NMR (162 MHz, $CDCl_3$) δ 20.5. ^1H NMR (400 MHz, $CDCl_3$) δ 7.56 – 7.41 (m, 2H), 7.39 – 7.08 (m, 13H), 7.07 – 6.84 (m, 4H), 6.11 – 6.00 (m, 1H), 5.11 – 4.93 (m, 2H), 4.90 – 4.79 (m, 1H), 4.78 – 4.71 (m, 1H), 4.51 – 4.40 (m, 1H), 2.27 (s, 3H). $^{13}C\{^1H\}$ NMR (101 MHz, $CDCl_3$) δ 143.1, 137.8 (d, *J* = 1.5 Hz), 136.0

(d, J = 6.0 Hz), 135.7 (d, J = 6.0 Hz), 133.6, 129.2, 128.7, 128.6, 128.6, 128.5, 128.4, 128.3, 128.3, 128.1, 127.9, 127.7, 127.6, 127.3, 127.2, 69.3 (d, J = 7.1 Hz), 68.9 (d, J = 7.0 Hz), 55.5 (d, J = 156.0 Hz), 21.5.
Chemical Formula: C28H28NO5PS / Exact Mass: 521,1426 / Internal reference PM-33C, PM-6 (rac)

Diethyl (S)-(((4-methylphenyl)sulfonamido)(p-tolyl)methyl)phosphonate (2e):

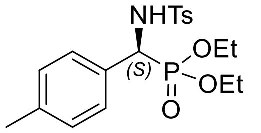

2e was obtained starting from diethyl(*p*-tolyl(tosylimino)methyl)phosphonate **1e** (81.9 mg, 0.2 mmol).
Yield = 90%, er = 99:1 (98% ee), $[\alpha]^{25}_{D}$ = -21.51 (c 0.93, $CHCl_3$). HPLC analysis: Chiralpak IA column, *n*-hexane/i-PrOH 65:35, flow = 0.7 mL/min, λ = 214 nm, t_R = 17.7 min (*R*, minor), t_R = 27.3 min (*S*, major).
$^{31}P\{^1H\}$ NMR (162 MHz, $CDCl_3$) δ 19.7. ^1H NMR (400 MHz, $CDCl_3$) δ 7.52 – 7.44 (m, 2H), 7.15 – 7.06 (m, 2H), 7.03 – 6.96 (m, 2H), 6.94 – 6.86 (m, 2H), 6.78 (m, 1H), 4.76 (dd, $^1J_{PH}$ = 24.1, $^1J_{NH}$ = 9.5 Hz, 1H), 4.40 – 4.13 (m, 2H), 3.90 (m, 1H), 3.62 (m, 1H), 2.31 (s, 3H), 2.27 (d, $^2J_{CH}$ = 1.7 Hz, 3H), 1.36 (td, $^3J_{PH}$ = 7.1, 0.5 Hz, 3H), 1.07 (td, $^3J_{PH}$ = 7.1, 0.5 Hz, 3H). $^{13}C\{^1H\}$ NMR (101 MHz, $CDCl_3$) δ 142.9, 138.0 (d, J_{PC} = 1.8 Hz), 137.7 (d, J_{PC} = 3.0 Hz), 130.7 (d, J_{PC} = 1.1 Hz), 129.1, 129.0 (d, J_{PC}= 2.3 Hz), 128.2 (d, J_{PC} = 6.0 Hz), 64.0 (d, J_{PC} = 7.1 Hz), 63.7 (d, J_{PC} = 6.9 Hz), 55.9, 54.3, 21.5, 21.2, 16.5 (d, J_{PC} = 6.0 Hz), 16.2 (d, J_{PC}= 5.7 Hz).
HRMS (ESI) m/z [M+H] + calcd for $C_{19}H_{27}NO_5PS$, 412.1342; found, 412.1335.
Chemical Formula: C19H26NO5PS / Exact Mass: 411,1269 / Internal reference PPL-(97)-rac, RC-023-ATH

Diethyl (S)-(((4-methylphenyl)sulfonamido)(m-tolyl)methyl)phosphonate (2f):

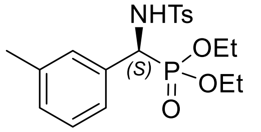

2f was obtained starting from diethyl(*m*-tolyl(tosylimino)methyl)phosphonate **1f** (81.9 mg, 0.2 mmol).
Yield = 89%, er = 97:3 (94% ee), $[\alpha]^{25}_{D}$ = -13,19 (c 0.91, $CHCl_3$). HPLC analysis: Chiralpak IA column, *n*-hexane/i-PrOH 65:35, flow = 0.7 mL/min, λ = 214 nm, t_R = 11.4 min (*R*, minor), t_R = 22.0 min (*S*, major).
$^{31}P\{^1H\}$ NMR (162 MHz, $CDCl_3$) δ 19.7. ^1H NMR (400 MHz, $CDCl_3$) δ 7.47 – 7.40 (m, 2H), 7.03 – 6.93 (m, 4H), 6.90 (m, 2H), 6.54 (s, 1H), 4.80 – 4.66 (dd, $^1J_{PH}$ = 24 Hz , $^1J_{NH}$ = 9.65Hz, 1H), 4.21 (m, 2H), 3.86 (m, 1H), 3.58 (m, 1H), 2.27 (s, 3H), 2.08 (s, 3H), 1.38 – 1.31 (m, 3H), 1.03 (td, $^3J_{PH}$ = 7.1, 0.6 Hz, 3H).$^{13}C\{^1H\}$ NMR (101 MHz, $CDCl_3$) δ 142.8, 138.0 (d, J_{PC} = 1.8 Hz), 137.8 (d, J_{PC} = 2.3 Hz), 133.4 (d, J_{PC} = 0.9 Hz), 129.0, 128.9, 128.9, 128.6 (d, J_{PC} = 3.0 Hz), 128.2 (d, J_{PC} = 2.3 Hz), 127.2, 125.5 (d, J_{PC} = 5.7 Hz), 64.1 (d, J_{PC} = 6.9 Hz), 63.7 (d, J_{PC} = 7.1 Hz), 56.2, 54.6, 21.4, 21.1, 16.6 (d, J_{PC} = 6.0 Hz), 16.2 (d, J_{PC} = 5.7 Hz).
HRMS (ESI) m/z [M+H] + calcd for $C_{19}H_{27}NO_5PS$, 412.1342; found, 412.1335.
Chemical Formula: C19H26NO5PS / Exact Mass: 411,13 / Internal Reference : RC-073-rac, RC-081-ATH

Diethyl (S)-((4-fluorophenyl)((4-methylphenyl)sulfonamido)methyl)phosphonate (2g):

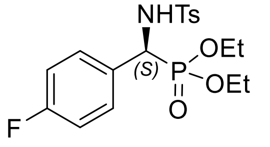

2g was obtained starting from diethyl((4-fluorophenyl)-(tosylimino)methyl)phosphonate **1g** (82.7 mg, 0.2 mmol).
Yield = 87%, er = 97.5:2.5 (95% ee), $[\alpha]^{25}_{D}$ = -11,32 (c 1.06, $CHCl_3$). HPLC analysis: Chiralpak IA column, *n*-hexane/i-PrOH 65:35, flow = 0.7 mL/min, λ = 214 nm, t_R = 18.7 min (*R*, minor), t_R = 27.7 min (*S*, major).
$^{31}P\{^1H\}$ NMR (162 MHz, $CDCl_3$) δ 19.3 (d, $^6J_{PF}$ = 4.7 Hz). $^{19}F\{^1H\}$ NMR (377 MHz, $CDCl_3$) δ -114.1 (d, $^6J_{FP}$ = 4.7 Hz). ^1H NMR (400 MHz, $CDCl_3$) δ 7.52 – 7.39 (m, 2H), 7.23 – 7.10 (m, 2H), 7.03 – 6.96 (m, 2H), 6.92 (dd, J = 9.3, 5.0 Hz, 1H), 6.83 – 6.69 (m, 2H), 4.77 (dd, J = 24.2, 9.2 Hz, 1H), 4.42 – 4.13 (m, 2H), 4.04 –

3.82 (m, 1H), 3.77 – 3.59 (m, 1H), 2.29 (s, 3H), 1.35 (t, *J* = 7.1 Hz, 3H), 1.07 (t, *J* = 7.1 Hz, 3H).^{13}C NMR (101 MHz, $CDCl_3$) δ 163.7 (d, J_{PC} = 3.32 Hz), 161.3 (d, J_{PC} = 3.32 Hz), 143.1, 138.1 (d, J_{PC} = 1.8 Hz), 130.1 (dd, J_{PC} = 8.2, 5.9 Hz), 129.7, 129.1, 127.2, 115.2 (d, J_{PC} = 2.2 Hz), 115.0 (d, J_{PC} = 2.2 Hz), 64.4 (d, J_{PC} = 7.3 Hz), 63.7 (d, J_{PC} = 7.0 Hz), 55.5, 53.9, 21.5, 16.6 (d, J_{PC} = 6.2 Hz), 16.2 (d, J_{PC} = 5.5 Hz).
HRMS (ESI) m/z [M+H] + calcd for $C_{18}H_{24}FNO_5PS$, 416.1091; found, 416.1094
Chemical Formula: C18H23FNO5PS / Exact Mass: 415,10 / Internal Reference RC-071-rac, RC-078 ATH

Diethyl (S)-((4-bromophenyl)((4-methylphenyl)sulfonamido)methyl)phosphonate (2h):

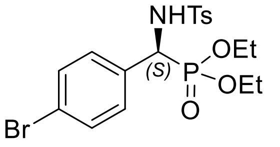

2h was obtained starting from diethyl((4-bromophenyl)-(tosylimino)methyl)phosphonate **1h** (94.9 mg, 0.2 mmol).
Yield = 91%, er = 98:2 (96% ee), $[\alpha]^{25}_D$ = -4,71 (c 0.85, $CHCl_3$). HPLC analysis: Chiralpak IA column, *n*-hexane/i-PrOH 65:35, flow = 0.7 mL/min, λ = 214 nm, t_R = 24.6 min (*R*, minor), t_R = 38.3 min (*S*, major).
$^{31}P\{^1H\}$ NMR (162 MHz, $CDCl_3$) δ 18.9.^{1}H NMR (400 MHz, $CDCl_3$) δ 7.44 – 7.40 (m, 2H), 7.21 – 7.15 (m, 2H), 7.07 – 7.01 (m, 2H), 7.01 – 6.96 (m, 2H), 6.80 (m, 1H), 4.79 – 4.66 (dd,$^1J_{PH}$ = 24.34 , $^1J_{NH}$ = 9.54 Hz, 1H), 4.26 (m, 2H), 3.91 (m, 1H), 3.70 (m, 1H), 2.32 (s, 3H), 1.36 (td, $^3J_{HP}$ = 7.1, 0.6 Hz, 3H), 1.10 (td, $^3J_{HP}$ = 7.1, 0.6 Hz, 3H).$^{13}C\{^1H\}$ NMR (101 MHz, $CDCl_3$) δ 143.2, 138.0 (d, J_{PC} = 2.1 Hz), 132.9, 131.3 (d, J_{PC} = 2.3 Hz), 130.1 (d, J_{PC} = 6.0 Hz), 129.1, 127.1, 122.0 (d, J_{PC} = 3.7 Hz), 64.5 (d, J_{PC} = 7.1 Hz), 63.7 (d, J_{PC} = 7.1 Hz), 55.7, 54.2, 21.5, 16.6 (d, J_{PC} = 6.0 Hz), 16.3 (d, J_{PC} = 5.5 Hz).
HRMS (ESI) m/z [M+H] + calcd for $C_{18}H_{24}BrNO_5PS$, 476.0291; found, 476.0279.
Chemical Formula: C18H23BrNO5PS / Exact Mass: 475,02 / Internal reference : RC-049-rac, RC-058 ATH

Diethyl (S)-((3-fluorophenyl)((4-methylphenyl)sulfonamido)methyl)phosphonate (2i):

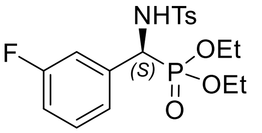

2i was obtained starting from diethyl((3-fluorophenyl)-(tosylimino)methyl)phosphonate **1i** (82.7 mg, 0.2 mmol).
Yield = 88%, er = 96:4 (92% ee), $[\alpha]^{25}_D$ = -13,33 (c 0.90, $CHCl_3$). HPLC analysis: Chiralpak IA column, *n*-hexane/i-PrOH 65:35, flow = 0.7 mL/min, λ = 214 nm, t_R = 12.5 min (*R*, minor), t_R = 19.4 min (*S*, major).
$^{31}P\{^1H\}$ NMR (162 MHz, $CDCl_3$) δ 19.0 (d, $^5J_{PF}$ = 1.8 Hz). $^{19}F\{^1H\}$ NMR (376 MHz, $CDCl_3$) δ -113.1 (d, $^5J_{FP}$ = 5.2 Hz). ^{1}H NMR (400 MHz, $CDCl_3$) δ 7.72 (m, 1H), 7.51 – 7.43 (m, 2H), 7.08 – 6.90 (m, 5H), 6.77 (tq, $^2J_{NP}$ = 8.2, $^1J_{NH}$= 1.9 Hz, 1H), 4.87 – 4.74 (dd, $^1J_{PH}$ = 24.7 Hz, $^1J_{NH}$ = 10.82 Hz, 1H), 4.30 (m, 2H), 3.89 (m, *J* = 10.1, 7.2 Hz, 1H), 3.64 (m, 1H), 2.24 (s, 3H), 1.38 (t, *J* = 7.1 Hz, 3H), 1.05 (t, *J* = 7.1 Hz, 3H.$^{13}C\{^1H\}$ NMR (101 MHz, $CDCl_3$) δ 163.8, 161.3, 143.0, 138.0, 136.3 (d, J_{PC} = 7.1 Hz), 129.7 (d, J_{PC}= 10.6 Hz), 129.0, 127.1, 124.2 (d, J_{PC} = 9.2 Hz), 115.4 (dd,J_{PC} = 22.8, 5.5 Hz), 114.7 (dd, J_{PC} = 21.4, 3.0 Hz), 64.5 (d, J_{PC} = 6.9 Hz), 63.8 (d, J_{PC} = 6.9 Hz), 55.9, 54.4, 21.4, 16.6 (d, J_{PC} = 6.0 Hz), 16.2 (d, J_{PC} = 5.5 Hz).
HRMS (ESI) m/z [M+H] + calcd for $C_{18}H_{24}FNO_5PS$, 416.1105; found, 416.1094.
Chemical Formula: C18H23FNO5PS / Exact Mass: 415,10 / Internal reference RC-052-rac, RC-066-ATH

Diethyl (S)-((2-fluorophenyl)((4-methylphenyl)sulfonamido)methyl)phosphonate (2j):

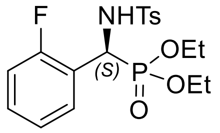

2j was obtained starting from diethyl((2-fluorophenyl)-(tosylimino)methyl)phosphonate **1j** (82.7 mg, 0.2 mmol).
Yield = 90%, er = 99:1 (98% ee), $[\alpha]^{25}_D$ = -13,04 (c 0.92, $CHCl_3$). HPLC analysis: Chiralpak IA column, *n*-hexane/i-PrOH 65:35, flow = 0.7 mL/min, λ = 214 nm, t_R = 15.5 min (*R*, minor), t_R = 35.0 min (*S*, major).

^{31}P{^{1}H} NMR (162 MHz, $CDCl_3$) δ 18.6 (d, $^{4}J_{PF}$ = 5.0 Hz).^{19}F{^{1}H} NMR (376 MHz, $CDCl_3$) δ -117.3 (d, $^{4}J_{FP}$ = 5.2 Hz).^{1}H NMR (400 MHz, $CDCl_3$) δ 7.51 (m, 2H), 7.34 (m, 1H), 7.10 (m, 1H), 6.98 (m, 2H), 6.89 – 6.77 (m, 2H), 6.70 (m, 1H), 5.20 – 5.08 (dd, $^{1}J_{PH}$ = 24.58 Hz , $^{1}J_{NH}$ = 10.15 Hz, 1H), 4.27 (m, 2H), 3.92 (m, 1H), 3.82 – 3.64 (m, 1H), 2.25 (s, 3H), 1.37 (t, J = 7.1 Hz, 3H), 1.05 (t, J = 7.1 Hz, 3H).^{13}C{^{1}H} NMR (101 MHz, $CDCl_3$) δ 161.2 (d, J_{PC} = 6.4 Hz), 158.7 (d, J_{PC} = 6.7 Hz), 143.1, 137.4, 129.8 (dd, J_{PC} = 4.5, 2.9 Hz), 129.6 (dd, J_{PC} = 8.3, 3.0 Hz), 129.2, 127.1, 124.2 (t, J_{PC} = 3.1 Hz), 121.5 (d, J_{PC} = 14.0 Hz), 115.1 (d, J_{PC} = 22.1 Hz), 64.5 (d, J_{PC} = 7.1 Hz), 63.8 (d, J_{PC} = 7.4 Hz), 48.9 (d, J_{PC} = 3.2 Hz), 47.3 (d, J_{PC} = 3.2 Hz), 21.5, 16.6 (d, J_{PC} = 5.7 Hz), 16.1 (d, J_{PC} = 5.7 Hz).

HRMS (ESI) m/z [M+H] + calcd for $C_{18}H_{24}FNO_5PS$, 416.1091; found, 416.1088.

Chemical Formula: C18H23FNO5PS / Exact Mass: 415,10 / Internal reference : RC-072-rac, RC-079-ATH

Diethyl (S)-((4-cyanophenyl)((4-methylphenyl)sulfonamido)methyl)phosphonate (2k):

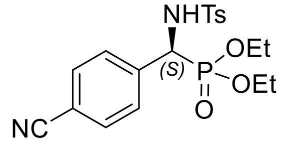

2k was obtained starting from diethyl((4-cyanophenyl)-(tosylimino)methyl)phosphonate **1k** (84.1 mg, 0.2 mmol). Yield = 87%, er = 92:8 (86% ee), $[\alpha]^{25}_{D}$ = -15,38 (c 0.78, $CHCl_3$). HPLC analysis: Chiralpak IA column, *n*-hexane/i-PrOH 65:35, flow = 0.7 mL/min, λ = 214 nm, t_R = 26.2 min (*R*, minor), t_R = 42.7 min (*S*, major).

^{31}P{^{1}H} NMR (162 MHz, $CDCl_3$) δ 18.4 (d, $^{1}J_{PH}$ = 23.2 Hz). ^{1}H NMR (400 MHz, $CDCl_3$) δ 7.49 – 7.43 (m, 2H), 7.41 – 7.30 (m, 4H), 7.02 (m, 2H), 6.84 (s, 1H), 4.90 – 4.75 (dd,$^{1}J_{PH}$ = 24.7 Hz ,6.67 Hz, 1H), 4.29 – 4.18 (m, 2H), 3.94 (m, 1H), 3.75 (m, 1H), 2.32 (s, 3H), 1.35 (m, 3H), 1.10 (t, J = 7.1 Hz, 3H).^{13}C{^{1}H} NMR (101 MHz, $CDCl_3$) δ 143.1, 137.9, 135.2, 132.9 (d, J_{PC} = 5.8 Hz), 131.6 (d, J_{PC} = 5.4 Hz), 131.1 (d, J_{PC}= 3.2 Hz), 129.0, 128.8, 127.0, 117.9, 112.4 (d, J_{PC} = 2.6 Hz), 65.1 (d, J_{PC} = 7.0 Hz), 64.0(d, J_{PC} = 7.2 Hz), 55.9, 54.3, 21.3, 16.5 (d, J_{PC} = 6.0 Hz), 16.2 (d, J_{PC} = 5.5 Hz).

HRMS (ESI) m/z [M+H] + calcd for $C_{19}H_{24}N_2O_5PS$, 423.1138; found, 423.1137.

Chemical Formula: C19H23N2O5PS / Exact Mass: 422,11 / Internal Reference : RC-063-rac, RC-085-ATH

Diethyl (S)-((3-cyanophenyl)((4-methylphenyl)sulfonamido)methyl)phosphonate (2l):

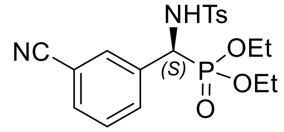

2l was obtained starting from diethyl((3-cyanophenyl)-(tosylimino)methyl)phosphonate **1l** (84.1 mg, 0.2 mmol). Yield = 84%, er = 88:12 (74% ee), $[\alpha]^{25}_{D}$ = -15,38 (c 0.78, $CHCl_3$). HPLC analysis: Chiralpak IA column, *n*-hexane/i-PrOH 65:35, flow = 0.7 mL/min, λ = 214 nm, t_R = 12.8 min (*R*, minor), t_R = 18.6 min (*S*, major).

^{31}P{^{1}H} NMR (162 MHz, $CDCl_3$) δ 18.5. ^{1}H NMR (400 MHz, $CDCl_3$) δ 7.78 (m, 1H), 7.49 – 7.35 (m, 5H), 6.98 – 6.92 (m, 2H), 4.93 – 4.79 (dd, $^{1}J_{PH}$= 24.97, $^{1}J_{NH}$ = 10.01 Hz, 1H), 4.38 (m, 2H), 3.96 (m, 1H), 3.73 (m, 1H), 2.27 (s, 3H), 1.43 (td, $^{3}J_{PH}$ = 7.1, 0.7 Hz, 3H), 1.12 (td,$^{3}J_{PH}$ = 7.1, 0.6 Hz, 3H).^{13}C{^{1}H} NMR (101 MHz, $CDCl_3$) δ 143.2, 137.9 (d, J_{PC} = 2.1 Hz), 135.2, 132.9 (d, J_{PC} = 5.7 Hz), 131.6 (d, J_{PC} = 5.5 Hz), 131.1 (d, J_{PC} = 3.0 Hz), 129.0, 128.8 (d, J_{PC} = 2.3 Hz), 127.0, 117.9, 112.4 (d, J_{PC} = 2.5 Hz), 65.1 (d, J_{PC} = 7.1 Hz), 64.0 (d, J_{PC} = 7.1 Hz), 55.9, 54.3, 21.3, 16.6 (d, J_{PC} = 6.0 Hz), 16.2 (d, J_{PC} = 5.5 Hz).

HRMS (ESI) m/z [M+H] + calcd for $C_{19}H_{24}N_2O_5PS$, 423.1138; found, 423.1139.

Chemical Formula: $C_{19}H_{23}N_2O_5PS$ / Exact Mass: 422,11 / Internal Reference : RC-064-rac, RC-083-ATH

Diethyl (S)-(((4-methylphenyl)sulfonamido)(4-nitrophenyl)methyl)phosphonate (2m):

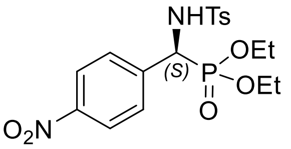

2m was obtained starting from diethyl((4-nitrophenyl)-(tosylimino)methyl)phosphonate **1m** (88.1 mg, 0.2 mmol).

Yellow solid, Yield = 81%, er = 82:18 (64% ee), $[\alpha]^{25}_{D}$ = -8,89 (c 0.90, $CHCl_3$). HPLC analysis: Chiralpak IA column, *n*-hexane/i-PrOH 65:35, flow = 0.7 mL/min, λ = 214 nm, t_R = 29.1 min (*R*, minor), t_R = 47.4 min (*S*, major).

^{1}P{^{1}H} NMR (162 MHz, $CDCl_3$) δ 18.0. ^{1}H NMR (400 MHz, $CDCl_3$) δ 7.95 (m, 2H), 7.50 – 7.44 (m, 2H), 7.42 – 7.35 (m, 2H), 7.05 – 6.97 (m, 2H), 6.86 – 6.69 (m, 1H), 4.94 – 4.82 (dd, $^{1}J_{PH}$ = 25.19 Hz , $^{1}J_{NH}$ = 9.9 Hz, 1H), 4.29 – 4.17 (m, 2H), 4.03 – 3.91 (m, 1H), 3.78 (m, 1H), 2.23 (s, 3H), 1.35 (tdd, $^{3}J_{PH}$ = 7.1, 0.7 Hz, 3H), 1.12 (t, *J* = 7.1, 3H).^{13}C{^{1}H} NMR (101 MHz, $CDCl_3$) δ 147.4 (d, J_{PC} = 3.4 Hz), 143.6, 141.4 (d, J_{PC} = 0.7 Hz), 137.7 (d, J_{PC} = 1.8 Hz), 129.3 (d, J_{PC} = 5.5 Hz), 129.2, 127.1, 123.2 (d, J_{PC} = 2.5 Hz), 64.9 (d, J_{PC} = 7.1 Hz), 63.9 (d, J_{PC} = 7.4 Hz), 55.8, 54.3, 21.4, 16.5 (d, J_{PC} = 6.0 Hz), 16.3 (d, J_{PC} = 5.5 Hz).

HRMS (ESI) m/z [M+H] + calcd for $C_{18}H_{24}N_2O_7PS$, 443.1036; found, 443.1035.

Chemical Formula: C18H23N2O7PS / Exact Mass: 442,0964 / Internal reference RC-053-rac, RC-034 ATH

Diethyl (S)-(((4-methylphenyl)sulfonamido)(3-nitrophenyl)methyl)phosphonate (2n):

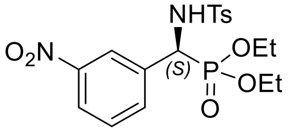

2m was obtained starting from diethyl((3-nitophenyl)-(tosylimino)methyl)phosphonate **1m** (88.1 mg, 0.2 mmol).
Yellow solid, Yield = 82%, er = 80:20 (60% ee), $[\alpha]^{25}_{D}$ = -8,89 (c 0.90, $CHCl_3$). HPLC analysis: Chiralpak IA column, *n*-hexane/i-PrOH 65:35, flow = 0.7 mL/min, λ = 214 nm, t_R = 15.1 min (*R*, minor), t_R = 19.5 min (*S*, major).

^{31}P{^{1}H} NMR (162 MHz, $CDCl_3$) δ 18.5. ^{1}H NMR (400 MHz, $CDCl_3$) δ 7.99 (m, 1H), 7.92 (m, 1H), 7.59 – 7.52 (m, 2H), 7.47 – 7.40 (m, 2H), 6.92 – 6.84 (m, 2H), 4.98 – 4.87 (dd,$^{1}J_{PH}$ = 25.43 Hz ,$^{1}J_{NH}$ = 10.52 Hz, 1H), 4.36 (m, 2H), 4.01 (m, 1H), 3.80 (m, 1H), 2.19 (s, 3H), 1.42 (td, $^{3}J_{PH}$ = 7.1, 0.7 Hz, 3H), 1.15 (td, $^{3}J_{PH}$= 7.1, 0.6 Hz, 3H). ^{13}C{^{1}H} NMR (101 MHz, $CDCl_3$) δ 147.9 (d, *J* = 3.0 Hz), 142.8, 138.1 (d, *J* = 2.3 Hz), 135.6, 134.7 (d, *J* = 6.0 Hz), 128.8, 126.9,123.3 (d, *J* = 5.1 Hz), 122.3 (d, *J* = 3.0 Hz), 65.1 (d, J_{PC} = 7.1 Hz), 63.9 (d, J_{PC} = 7.4 Hz), 56.0, 54.4, 21.1, 16.5 (d, J_{PC} = 6.0 Hz), 16.2 (d, J_{PC} = 5.5 Hz).

HRMS (ESI) m/z [M+H] + calcd for $C_{18}H_{24}N_2O_7PS$, 443.1036; found, 443.1038.

Chemical Formula: C18H23N2O7PS / Exact Mass: 442,10 / Internal Reference RC-047-rac, RC-065-ATH

5. General procedure for ATH of six-membered cyclic α-iminophosphonates 4a-f

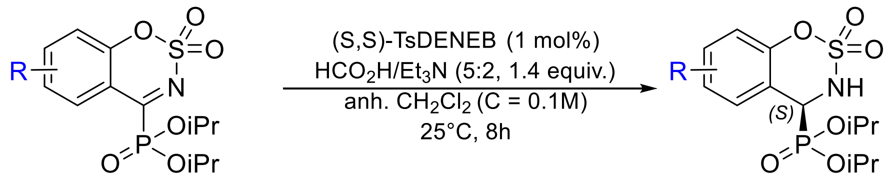

3a-f **4a-f**

In a sealed tube under an argon atmosphere, α-iminophosphonate (0.2 mmol) and (*S*,*S*)-Ts-DENEB catalyst (1.3 mg, 1 mol%) are dissolved in anhydrous CH_2Cl_2 (2 mL). The setup undergoes three cycles of vacuum and argon backfill to ensure an inert environment. Subsequently, HCO_2H:Et_3N (5:2, 0.024 mL, 1.4 equiv.) is added dropwise, and the reaction mixture is stirred at room temperature for 16 hours. Upon completion as monitored by ^{31}P NMR, the reaction mixture was concentrated *under vacuum* and purified by column chromatography on silica gel (AcOEt/petroleum ether).

The catalyst optimization studies yielded the following results:

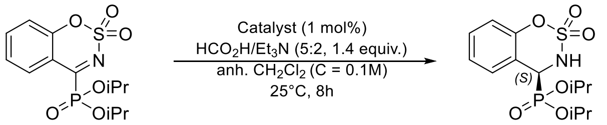

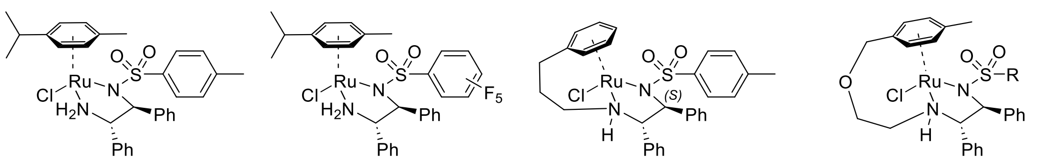

(*S*,*S*)-Ru-cat. **A**	(*S*,*S*)-Ru-cat. **B**	(*S*,*S*)-Ru-cat. **C**	(*S*,*S*)-Ru-cat. **D** (R = *p*-tolyl)
Yield : 88%	Yield : 91%	Yield : 89%	Yield : 97%
er : 62.5 : 37.5	er : 50.5 : 49.5	er : 85.5 : 14.5	er : 99.5 : 0.5

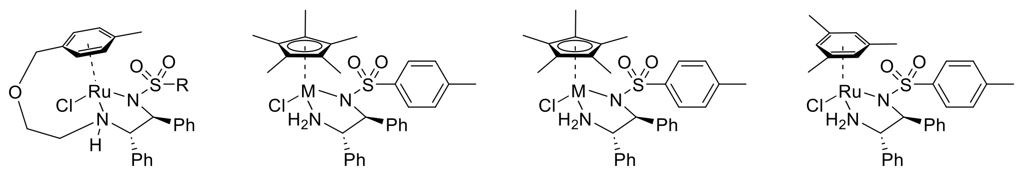

(*S*,*S*)-Ru-cat. **E** (R = CH_3)	(*S*,*S*)-Ir-cat. **F**	(*S*,*S*)-Rh-cat. **G**	(*S*,*S*)-Ru-cat. **H**
Yield : 91%	Yield : 86%	Yield : 93%	Yield : 91%
er : 92.5 : 7.5	er : 67.5 : 32.5	er : 84 : 16	er : 70 : 30

(S)-3,4-Dihydro-1,2,3-benzoxathiazine-2,2-dioxide-4-diisopropylphosphonate (4a) (known compound[3]):

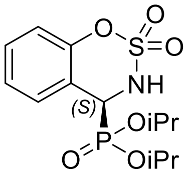

4a was obtained starting from diisopropyl (2,2-dioxidobenzo[e][1,2,3]oxathiazin-4-yl)phosphonate **3a** (69.5 mg, 0.2 mmol).

Yield = 97%, er = 99.5:0.5 (99% ee), $[\alpha]^{25}_D$ = -60,4 (c 1.1, $CHCl_3$). HPLC analysis: Chiralpak AD-H column, *n*-hexane/i-PrOH 80:20, flow = 0.8 mL/min, λ = 220 nm, t_R = 7.5 min (*S*, major), t_R = 8.4 min (*R*, minor).

$^{31}P\{^1H\}$ NMR (162 MHz, $CDCl_3$) δ 14.4. ^1H NMR (400 MHz, Chloroform-*d*) δ 7.88 – 7.80 (m, 1H), 7.38 – 7.29 (m, 1H), 7.23 – 7.17 (m, 1H), 7.05 – 7.00 (m, 1H), 5.08 (d, *J* = 20.3 Hz, 1H), 4.87 – 4.73 (m, 1H), 4.73 – 4.60 (m, 1H), 1.37 (dd, *J* = 9.4, 6.2 Hz, 6H), 1.32 (d, *J* = 6.2 Hz, 3H), 1.08 (d, *J* = 6.2 Hz, 3H). ^{13}C $\{^1H\}$ NMR (101 MHz, Chloroform-*d*) δ 151.7 (d, J_{PC} = 8.9 Hz), 130.0 (d, J_{PC} = 2.0 Hz), 128.0 (d, J_{PC} = 3.4 Hz), 125.5 (d, J_{PC} = 2.3 Hz), 119.3, 116.4 (d, J_{PC} = 4.1 Hz), 73.8 (d, J_{PC} = 7.1 Hz), 73.4 (d, J_{PC} = 6.8 Hz), 54.4 (d, J_{PC} = 152.9 Hz), 24.2 (d, J_{PC} = 3.4 Hz), 24.1 (d, J_{PC} = 4.0 Hz), 23.9 (d, J_{PC} = 4.8 Hz), 23.5 (d, J_{PC} = 5.3 Hz). HRMS (ESI) m/z [M+H] + calcd for $C_{13}H_{21}NO_6PS$, 350.0822; found, 350.0825.
Chemical Formula: C13H20NO6PS / Exact Mass: 349.34 / Internal Reference LB-505, LB-517

(S)-3,4-Dihydro-1,2,3-benzoxathiazine-2,2-dioxide-7-methyl-4-diisopropylphosphonate (4c) (known compound[3]):

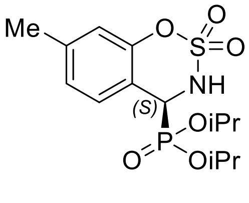

4c was obtained starting from diisopropyl (7-methyl-2,2-dioxidobenzo[e][1,2,3]oxathiazin-4-yl)phosphonate **3c** (72.3 mg, 0.2 mmol).

Yield = 96%, er = 99.5:0.5 (99% ee), $[\alpha]^{25}_D$ = -74,5 (c 1.0, $CHCl_3$). HPLC analysis: Chiralpak AD-H column, *n*-hexane/i-PrOH 90:10, flow = 0.8 mL/min, λ = 220 nm, t_R = 11.0 min (*S*, major), t_R = 13.9 min (*R*, minor).

$^{31}P\{^1H\}$ NMR (162 MHz, $CDCl_3$) δ 14.6. ^1H NMR (400 MHz, Chloroform-*d*) δ 7.74 – 7.67 (m, 1H), 7.05 – 6.97 (m, 1H), 6.87 – 6.80 (m, 1H), 5.04 (d, *J* = 20.0 Hz, 1H), 4.87 – 4.73 (m, 1H), 4.72 – 4.59 (m, 1H),

1.36 (dd, *J* = 8.3, 6.2 Hz, 6H), 1.32 (d, *J* = 6.1 Hz, 3H), 1.09 (d, *J* = 6.2 Hz, 3H). $^{13}C\{^1H\}$ NMR (101 MHz, Chloroform-*d*) δ 151.5 (d, J_{PC} = 9.0 Hz), 140.6 (d, J_{PC} = 2.2 Hz), 127.7 (d, J_{PC} = 3.3 Hz), 126.5 (d, J_{PC} = 2.3 Hz), 119.5, 113.1 (d, J_{PC} = 3.9 Hz), 73.7 (d, J_{PC} = 7.0 Hz), 73.3 (d, J_{PC} = 6.9 Hz), 54.2 (d, J_{PC} = 153.1 Hz), 24.2 (d, J_{PC} = 3.4 Hz), 24.1 (d, J_{PC} = 4.0 Hz), 23.9 (d, J_{PC} = 4.9 Hz), 23.6 (d, J_{PC} = 5.3 Hz), 21.1.
HRMS (ESI) m/z [M+H] + calcd for $C_{14}H_{23}NO_6PS$, 364.0978; found, 364.0978.
Chemical Formula: C14H23NO6PS / Exact Mass: 363,36 / Internal Reference LB-530, LB-535

(S)-3,4-Dihydro-1,2,3-benzoxathiazine-2,2-dioxide-8-methoxy-4-diisopropylphosphonate (4d) (known compound[3]):

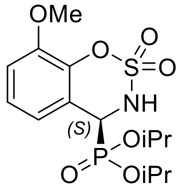

4d was obtained starting from diisopropyl (8-methoxy-2,2-dioxidobenzo[e][1,2,3]oxathiazin-4-yl)phosphonate **3d** (75.5 mg, 0.2 mmol).
Yield = 90%, er = 96.5:3.5 (93% ee), $[\alpha]^{25}_D$ = -60.6 (c 1.0, $CHCl_3$). HPLC analysis: Chiralpak IG column, Heptane/Ethanol 80:20, flow = 1 mL/min, λ = 220 nm, t_R = 10.8 min (*S*, major), t_R = 12.8 min (*R*, minor).
$^{31}P\{^1H\}$ NMR (162 MHz, $CDCl_3$) δ 14.4. ^1H NMR (400 MHz, Chloroform-*d*) δ 7.42 (d, *J* = 8.0 Hz, 1H), 7.14 (t, *J* = 8.2 Hz, 1H), 6.93 (d, *J* = 8.2 Hz, 1H), 5.09 (d, *J* = 20.3 Hz, 1H), 4.81 (dq, *J* = 12.3, 6.1 Hz, 1H), 4.70 (dq, *J* = 13.3, 6.2 Hz, 1H), 3.87 (s, 3H), 1.37 (dd, *J* = 7.4, 6.2 Hz, 6H), 1.32 (d, *J* = 6.2 Hz, 3H), 1.08 (d, *J* = 6.2 Hz, 3H). $^{13}C\ \{^1H\}$ NMR (101 MHz, Chloroform-*d*) δ 149.2 (d, *J* = 0.7 Hz), 141.4 (d, *J* = 9.1 Hz), 125.2 (d, *J* = 2.3 Hz), 119.0 (d, *J* = 3.4 Hz), 117.4 (d, *J* = 3.7 Hz), 112.5 (d, *J* = 112.5 Hz), 73.7 (d, *J* = 7.0 Hz), 73.4 (d, *J* = 6.8 Hz), 56.4, 54.6 (d, *J* = 152.3 Hz), 24.2 (d, *J* = 3.4 Hz), 24.2 (d, *J* = 4.0 Hz), 23.9 (d, *J* = 4.8 Hz), 23.6 (d, *J* = 5.3 Hz).
HRMS (ESI) m/z [M+H] + calcd for $C_{14}H_{22}NO_7PS$, 380.0927; found, 380.0927.
Chemical Formula: C14H22NO7PS / Exact Mass: 379.09 / Internal Reference BPO-127, BPO-137.

(S)-3,4-Dihydro-1,2,3-benzoxathiazine-2,2-dioxide-7-methoxy-4-diisopropylphosphonate (4e) (known compound[3]):

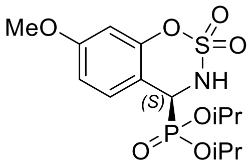

4e was obtained starting from diisopropyl (7-methoxy-2,2-dioxidobenzo[e][1,2,3]oxathiazin-4-yl)phosphonate **3e** (75.5 mg, 0.2 mmol).
Yield = 87%, er = 97.5:2.5 (95% ee), $[\alpha]^{25}_D$ = -64.6 (c 1.0, $CHCl_3$). HPLC analysis: Chiralpak IF column, Heptane/Ethanol 80:20, flow = 1 mL/min, λ = 220 nm, t_R = 10.6 min (*S*, major), t_R = 16.0 min (*R*, minor).
$^{31}P\{^1H\}$ NMR (162 MHz, $CDCl_3$) δ 14.6. ^1H NMR (400 MHz, Chloroform-*d*) δ 7.75 (d, *J* = 9.1 Hz, 1H), 6.74 (dd, *J* = 8.9, 2.6 Hz, 1H), 6.54 (d, *J* = 2.6 Hz, 1H), 5.01 (d, *J* = 19.6 Hz, 1H), 4.80 (dp, *J* = 7.2, 6.1 Hz, 1H), 4.66 (dp, *J* = 7.2, 6.1 Hz, 1H), 3.79 (s, 3H), 1.37 (t, *J* = 6.2 Hz, 6H), 1.32 (d, *J* = 6.2 Hz, 3H), 1.10 (d, *J* = 6.2 Hz, 3H). $^{13}C\ \{^1H\}$ NMR (101 MHz, Chloroform-*d*) δ 160.7 (d, *J* = 1.8 Hz), 152.2 (d, *J* = 9.2 Hz), 128.7 (d, *J* = 3.2 Hz), 112.2 (d, *J* = 2.1 Hz), 107.8 (d, *J* = 3.9 Hz), 104.2 (d, *J* = 0.7 Hz), 73.7 (d, *J* = 7.0 Hz), 73.3 (d, *J* = 6.9 Hz), 55.7, 53.9 (d, *J* = 153.3 Hz), 24.2 (d, *J* = 3.3 Hz), 24.1 (d, *J* = 4.0 Hz), 23.9 (d, *J* = 4.8 Hz), 23.6 (d, *J* = 5.2 Hz).
HRMS (ESI) m/z [M+H] + calcd for $C_{14}H_{22}NO_7PS$, 380.0927; found, 380.0928.
Chemical Formula: C14H22NO7PS / Exact Mass: 379.09 / Internal Reference BPO-129, BPO-138.

(S)-3,4-Dihydro-1,2,3-benzoxathiazine-2,2-dioxide-6-methoxy-4-diisopropylphosphonate (4f)

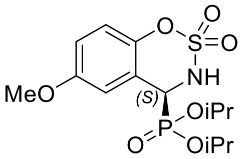

(known compound[3]):

4f was obtained starting from diisopropyl (6-methoxy-2,2-dioxidobenzo[e][1,2,3]oxathiazin-4-yl)phosphonate **3f** (75.5 mg, 0.2 mmol).

Yield = 88%, er = 96:4 (92% ee), $[\alpha]^{25}_{D}$ = -73.8 (c 1.0, $CHCl_3$). HPLC analysis: Chiralpak IF column, Heptane/Ethanol 80:20, flow = 1 mL/min, λ = 220 nm, t_R = 9.5 min (*S*, major), t_R = 12.7 min (*R*, minor). $^{31}P\{^1H\}$ NMR (162 MHz, $CDCl_3$) δ 14.5. ^1H NMR (400 MHz, Chloroform-*d*) δ 7.39-7.42 (m, 1 H), 6.96 (dd, *J* = 9.0, 1.0 Hz, 1 H), 6.89-6.55 (m, 1 H), 5.08-5.00 (m, 1 H), 4.82 (dp, *J* = 7.4, 6.2 Hz, 1H), 4.68 (dp, *J* = 7.4, 6.2 Hz, 1H), 3.79 (s, 1 H), 1.39 (t, *J* = 5.9 Hz, 6 H), 1.33 (d, *J* = 6.2 Hz, 3 H), 1.10 (d, *J* = 6.2 Hz, 3 H). $^{13}C\ \{^1H\}$ NMR (101 MHz, Chloroform-*d*) δ 156.8 (d, *J* = 2.3 Hz), 145.4 (d, *J* = 9.0 Hz), 120.2 (d, *J* = 0.9 Hz), 116.8 (d, *J* = 4.0 Hz), 116.5 (d, *J* = 2.3 Hz), 111.9 (d, *J* = 3.2 Hz), 73.8 (d, *J* = 7.0 Hz), 73.4 (d, *J* = 6.9 Hz), 55.9, 54.5 (d, *J* = 151.9 Hz), 24.2 (d, *J* = 3.4 Hz), 24.2 (d, *J* = 4.0 Hz), 23.9 (d, *J* = 4.9 Hz), 23.6 (d, *J* = 5.3 Hz).

HRMS (ESI) m/z [M+H] + calcd for $C_{14}H_{22}NO_7PS$, 380.0927; found, 380.0929.

Chemical Formula: C14H22NO7PS / Exact Mass: 379.09 / Internal Reference BPO-128, BPO-139.

6. General procedure for ATH of five-membered cyclic α-iminophosphonates 4g-i

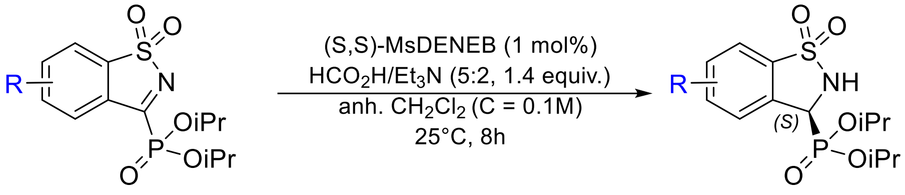

3g-i **4g-i**

In a microwave tube under an argon atmosphere, α-iminophosphonates (0.2 mmol) and (S,S)-Ms-DENEB catalyst (1.1 mg, 1 mol%), are dissolved in anhydrous CH_2Cl_2 (2 mL). The setup undergoes three cycles of vacuum and arsgon backfill to ensure an inert environment. Subsequently, HCO_2H:Et_3N (5:2, 0.024 mL, 1.4 equiv.) is added dropwise, and the reaction mixture is stirred at room temperature for 16 hours. Upon completion as monitored by ^{31}P NMR, the reaction mixture was concentrated *under vacuum* and purified by column chromatography on silica gel (AcOEt/petroleum ether).

The catalyst optimization studies yielded the following results:

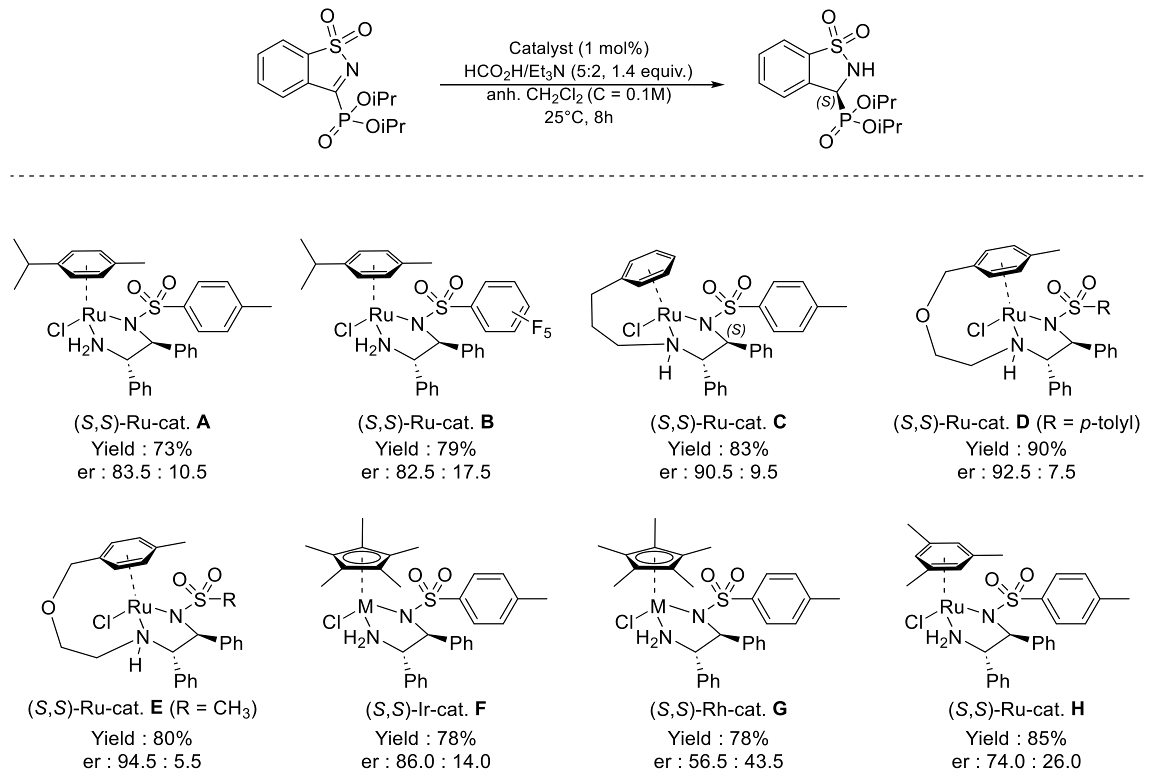

(S)-Diisopropyl(1,1-dioxido-2,3-dihydrobenzo[d]isothiazol-3-yl)phosphonate (4g):

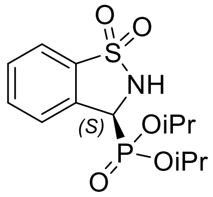

4g was obtained starting from diisopropyl (1,1-dioxidobenzo[d]isothiazol-3-yl)phosphonate **3g** (66.3 mg, 0.2 mmol).
Yield = 80%, er = 94.5:5.5 (89% ee), $[\alpha]^{25}_D$ = -27,2 (c 1.1, CHCl3). HPLC analysis: Chiralpak OD column, *n*-hexane/i-PrOH 80:20, flow = 0.8 mL/min, λ = 220 nm, t_R = 10.2 min (*R*, minor), t_R = 12.0 min (*S*, major);
$^{31}P\{^1H\}$ NMR (162 MHz, $CDCl_3$) δ 14.6. ^1H NMR (400 MHz, Chloroform-*d*) δ 7.82-7.77 (m, 2 H), 7.66 (td, *J* = 7.6, 1.1 Hz, 1 H), 7.61-7.56 (m, 1 H), 4.98 (d, *J* = 11.3 Hz, 1 H), 4.82 (dp, *J* = 7.1, 6.1 Hz, 1H), 4.59 (dp, *J* = 7.2, 6.2 Hz, 1H), 1.35 (d, *J* = 6.2 Hz, 3 H), 1.29 (t, *J* = 6.1 Hz, 6 H), 1.02 (d, *J* = 6.2 Hz, 3 H). $^{13}C\ \{^1H\}$ NMR (101 MHz, Chloroform-*d*) δ 135.2 (d, *J* = 6.0 Hz), 133.3 (d, *J* = 2.6 Hz), 132.7 (d, *J* = 6.0 Hz), 130.0 (d, *J* = 2.5 Hz), 126.0 (d, *J* = 3.0 Hz), 121.6 (d, *J* = 1.9 Hz), 73.5 (d, *J* = 1.9 Hz), 73.4 (d, *J* = 1.9 Hz), 54.6 (d, *J* = 160.6 Hz), 24.3 (d, *J* = 1.7 Hz), 24.2 (d, *J* = 2.0 Hz), 23.9 (d, *J* = 5.3 Hz), 23.5 (d, *J* = 5.1 Hz).
HRMS (ESI) m/z [M+H] + calcd for $C_{13}H_{20}NO_5PS$, 334.0872; found, 334.0872.
Chemical Formula: C13H20NO5PS / Exact Mass: 333.08 / Internal Reference BPO-148, BPO-153.

(S)-Diisopropyl(5-methyl-1,1-dioxido-2,3-dihydrobenzo[d]isothiazol-3-yl)phosphonate (4h):

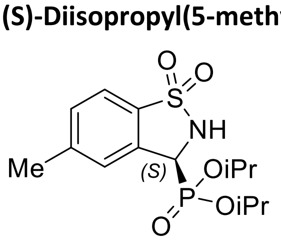

4h was obtained starting from diisopropyl (5-methyl-1,1-dioxidobenzo[d]isothiazol-3-yl)phosphonate **3h** (69.1 mg, 0.2 mmol).
Yield = 84%, er = 95:5 (90% ee), $[\alpha]^{25}_D$ = -46,15 (c 1.1, $CHCl_3$). HPLC analysis: Chiralpak IK column, Heptane/Ethanol 60:40, flow = 1 mL/min, λ = 220 nm, t_R = 6.9 min (*S*, major), t_R = 8.9 min (*R*, minor).
$^{31}P\{^1H\}$ NMR (162 MHz, $CDCl_3$) δ 14.8. ^1H NMR (400 MHz, Chloroform-*d*) δ 7.68 (d, *J* = 8.0, 1 H), 7.59 (s, 1 H), 7.38 (d, *J* = 8.0 Hz, 1 H), 4.92 (d, *J* = 11.4 Hz, 1 H), 4.82 (dp, *J* = 7.2, 6.2 Hz, 1H), 4.60-4.51 (m, 1H), 2.48 (s, 3H), 1.36 (d, *J* = 6.2 Hz, 3 H), 1.30 (t, *J* = 6.4 Hz, 6 H), 1.00 (d, *J* = 6.2 Hz, 3 H). $^{13}C\ \{^1H\}$ NMR (101 MHz, Chloroform-*d*) δ 144.4 (d, *J* = 2.5 Hz), 132.9 (d, *J* = 6.0 Hz), 132.5 (d, *J* = 6.0 Hz), 131.0 (d, *J* = 2.5

Hz), 126.1 (d, *J* = 3.0 Hz), 121.4 (d, *J* = 2.0 Hz), 73.4 (d, *J* = 7.3 Hz), 73.3 (d, *J* = 7.1 Hz), 54.5 (d, *J* = 160.1 Hz), 24.3 (d, *J* = 1.2 Hz), 24.2 (d, *J* = 1.1 Hz), 24.0 (d, *J* = 5.3 Hz), 23.5 (d, *J* = 5.1 Hz), 22.0.
HRMS (ESI) m/z [M+H] + calcd for $C_{14}H_{22}NO_5PS$, 348.1029; found, 348.1029.
Chemical Formula: C14H20NO5PS / Exact Mass: 347.096 / Internal Reference BPO-151, BPO-161

(S)-diisopropyl(5-fluoro-1,1-dioxido-2,3-dihydrobenzo[d]isothiazol-3-yl)phosphonate (4i):

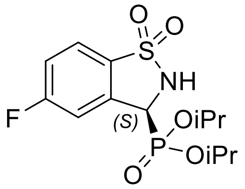

4i was obtained starting from diisopropyl(5-fluoro-1,1-dioxidobenzo[d]isothiazol-3-yl)phosphonate **3i** (69.9 mg, 0.2 mmol)
Yield = 68%, er = 90:10 (80% ee), $[\alpha]^{25}_{D}$ = -27,7 (c 1.1, $CHCl_3$). HPLC analysis: Chiralpak IB column, *n*-hexane/i-PrOH 88:12, flow = 0.8 mL/min, λ = 220 nm, t_R = 17.2 min (*R*, minor), t_R = 22.2 min (*S*, major).
$^{31}P\{^1H\}$ NMR (162 MHz, $CDCl_3$) δ 14.4. $^{19}F\{^1H\}$ NMR (376 MHz, $CDCl_3$) δ -103.9. ^1H NMR (400 MHz, Chloroform-*d*) δ 7.84 – 7.71 (m, 1H), 7.47 – 7.40 (m, 1H), 7.29 – 7.22 (m, 1H), 4.96 (dd, *J* = 11.9, 4.5 Hz, 1H), 4.90 – 4.77 (m, *J* = 6.2 Hz, 1H), 4.75 – 4.63 (m, 1H), 1.34 (dd, *J* = 6.2, 1.6 Hz, 6H), 1.25 (dt, *J* = 6.2, 1.6 Hz, 3H), 1.17 (dd, *J* = 6.3, 2.3 Hz, 3H). $^{13}C\ \{^1H\}$ NMR (101 MHz, Chloroform-*d*) δ 165.4 (dd, J_{PC} = 254.9, 3.0 Hz), 136.0 (dd, J_{PC} = 10.2, 6.1 Hz), 131.3 (dd, J_{PC} = 5.7, 2.5 Hz), 123.8 (dd, J_{PC} = 10.0, 2.1 Hz), 118.0 (dd, J_{PC} = 24.3, 1.8 Hz), 113.1 (dd, J_{PC} = 25.3, 2.8 Hz), 74.0 (d, J_{PC} = 6.5 Hz), 73.5 (d, J_{PC} = 7.4 Hz), 54.3 (dd, J_{PC} = 162.8, 2.4 Hz), 24.3 (d, J_{PC} = 3.3 Hz), 24.2 (d, J_{PC} = 3.8 Hz), 23.8 (d, J_{PC} = 2.4 Hz), 23.8 (d, J_{PC} = 1.5 Hz).
HRMS (ESI) m/z [M+H] + calcd for $C_{13}H_{19}FNO_5PS$, 352.0778; found, 352.0779.
Chemical Formula: C13H19FNO5PS / Exact Mass: 351.071 / Internal Reference BPO-190, LB-553

7. ^{1}H, ^{31}P{^{1}H}, ^{19}F and ^{13}C{^{1}H} NMR spectra of 2a-n and 4a-i

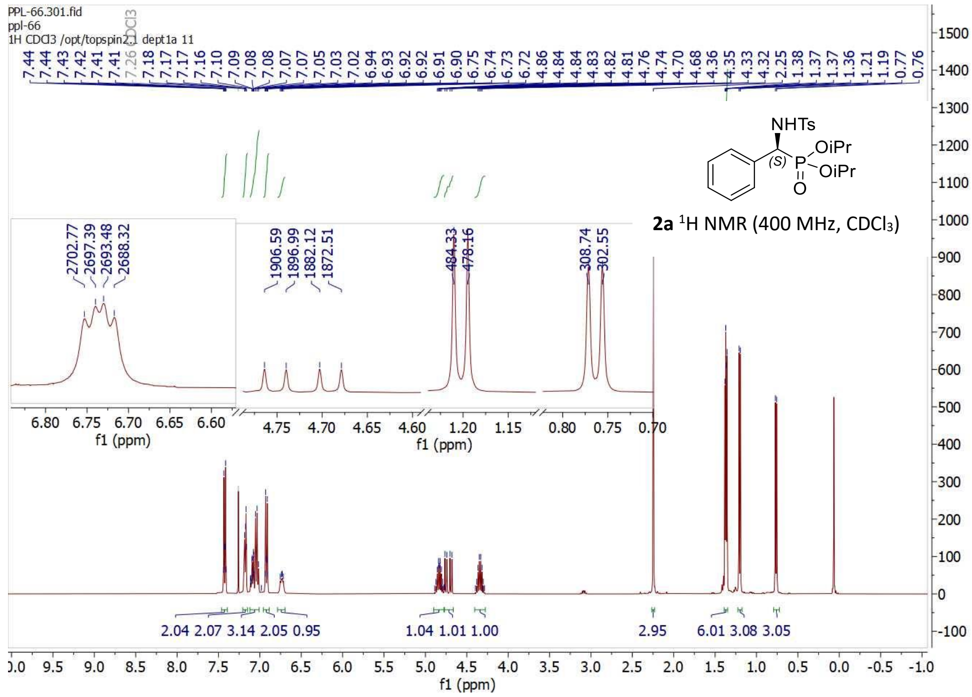

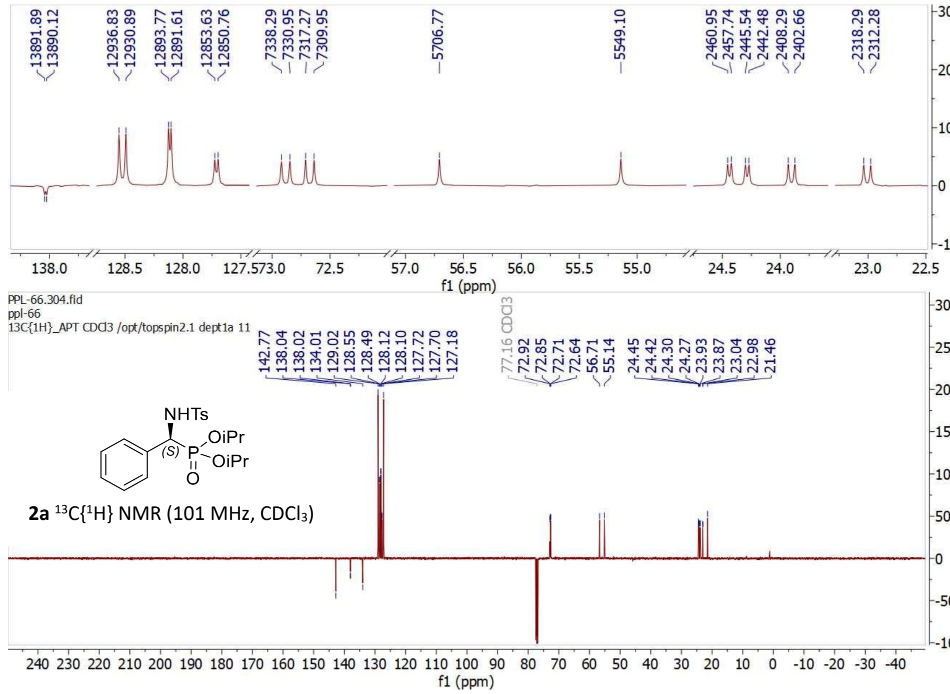

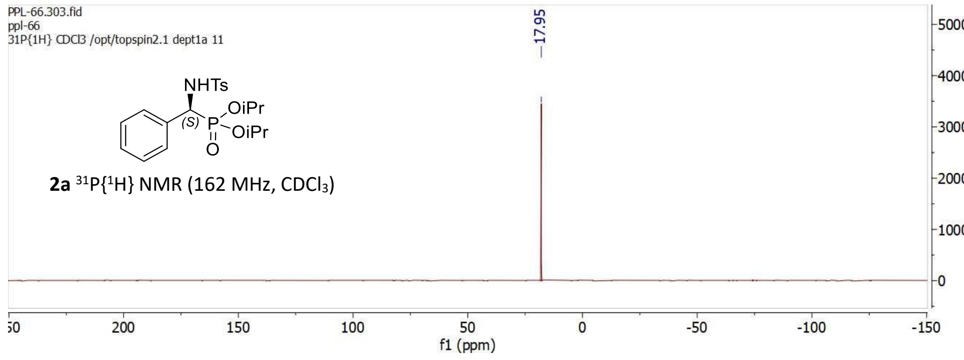

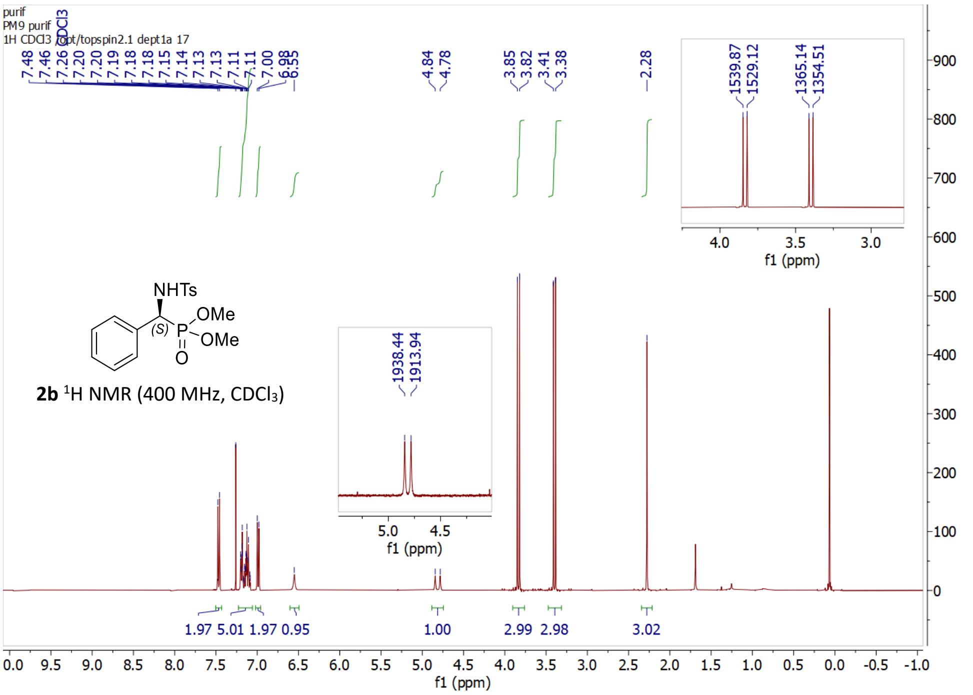

2b ^{1}H NMR (400 MHz, $CDCl_3$)

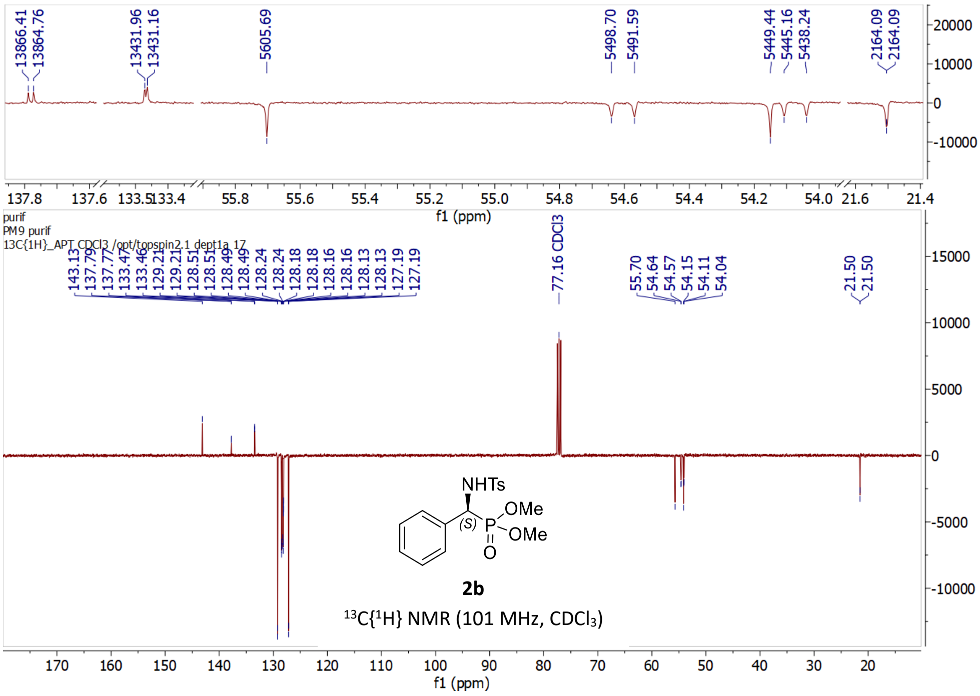

2b

$^{13}C\{^{1}H\}$ NMR (101 MHz, $CDCl_3$)

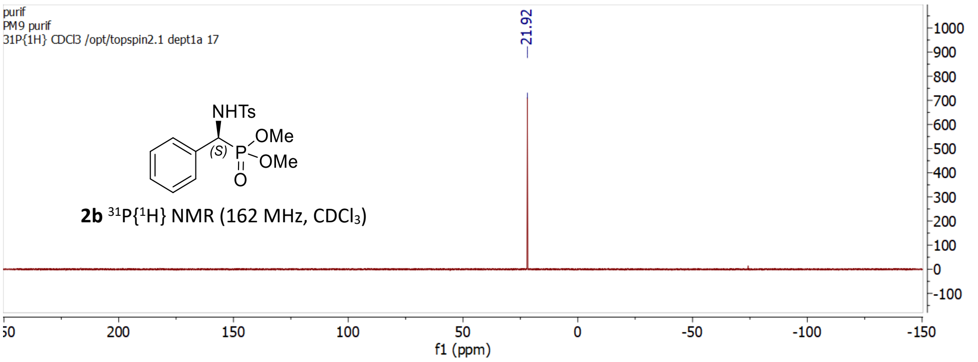

2b $^{31}P\{^{1}H\}$ NMR (162 MHz, $CDCl_3$)

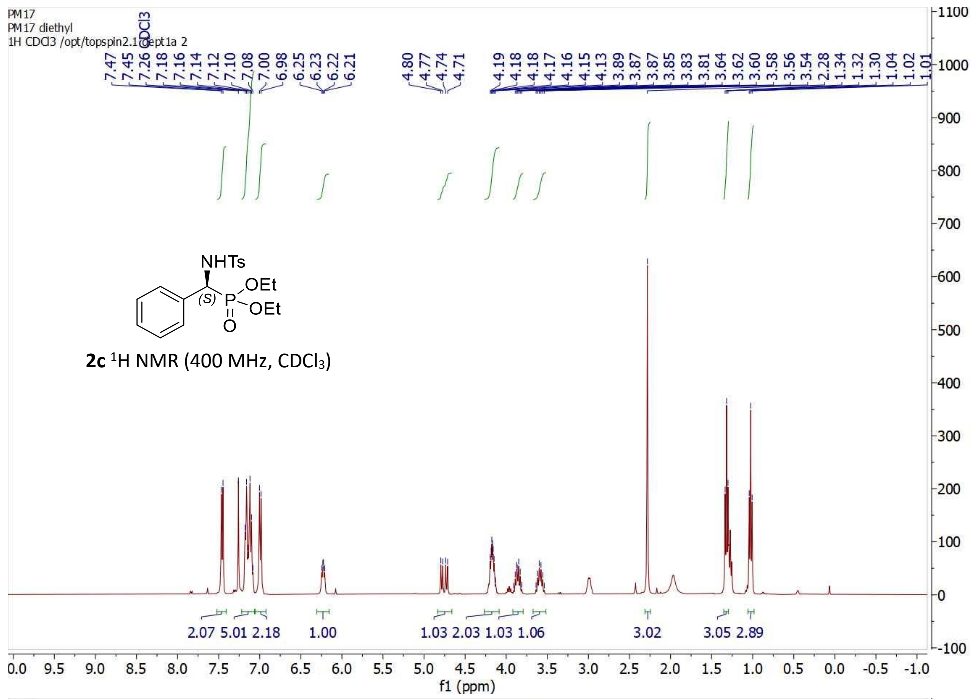
PM17
PM17 diethyl
1H CDCl3 /opt/topspin2.1 dept1a 2
NHTs
(S)
P
OEt
OEt
O
2c ^{1}H NMR (400 MHz, CDCl$_3$)
2.07 5.01 2.18 1.00 1.03 2.03 1.03 1.06 3.02 3.05 2.89
f1 (ppm)

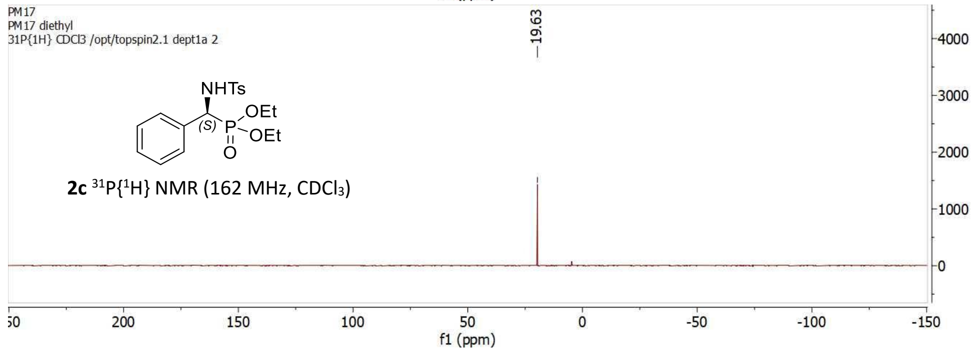
PM17
PM17 diethyl
31P{1H} CDCl3 /opt/topspin2.1 dept1a 2
19.63
NHTs
(S)
P
OEt
OEt
O
2c $^{31}P\{^{1}H\}$ NMR (162 MHz, CDCl$_3$)
f1 (ppm)

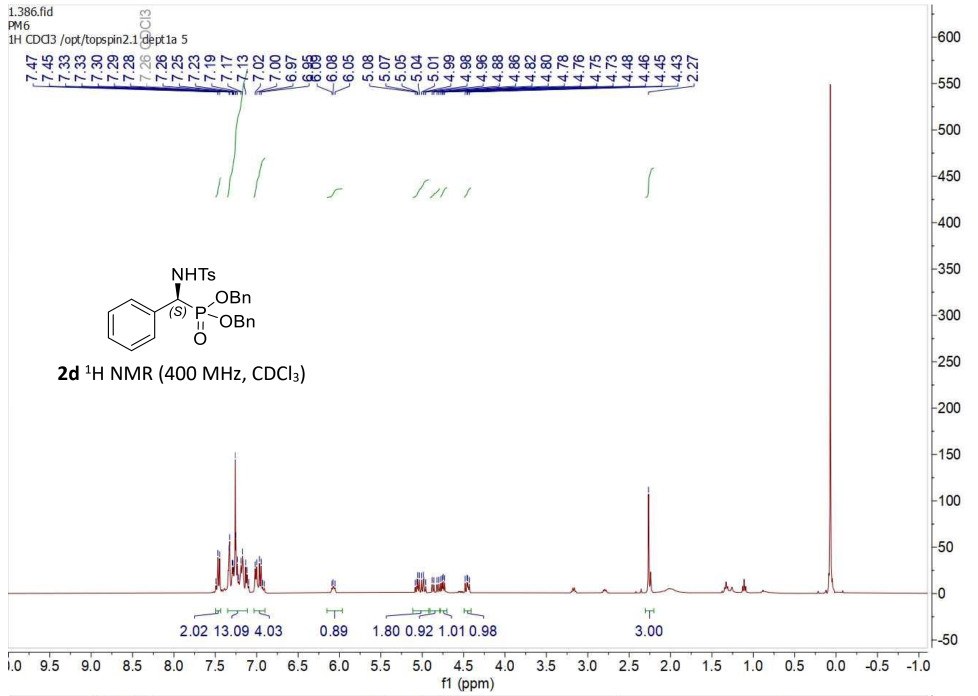
NHTs
(S)
OBn
OBn
O
2d ¹H NMR (400 MHz, CDCl₃)
f1 (ppm)

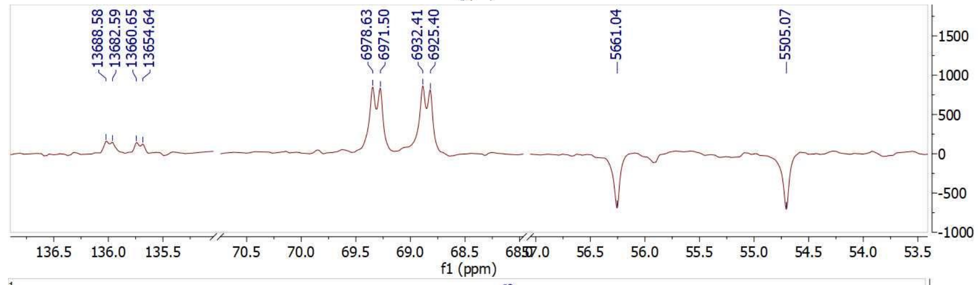
f1 (ppm)

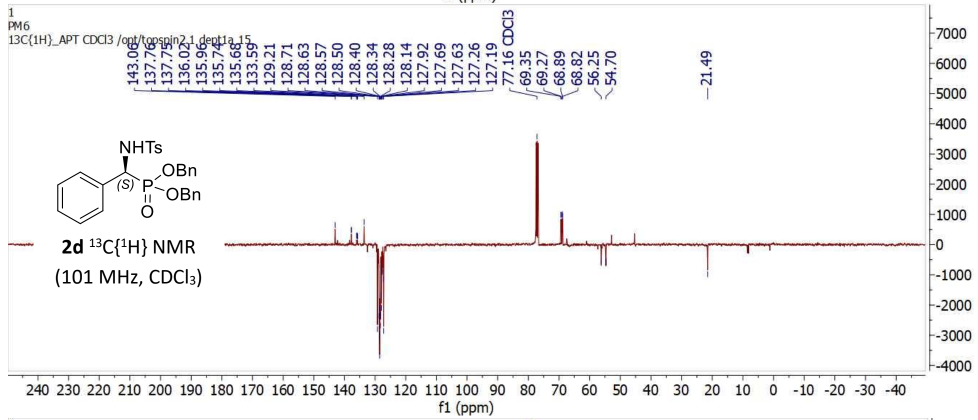
NHTs
(S)
OBn
OBn
O
2d ¹³C{¹H} NMR
(101 MHz, CDCl₃)
f1 (ppm)

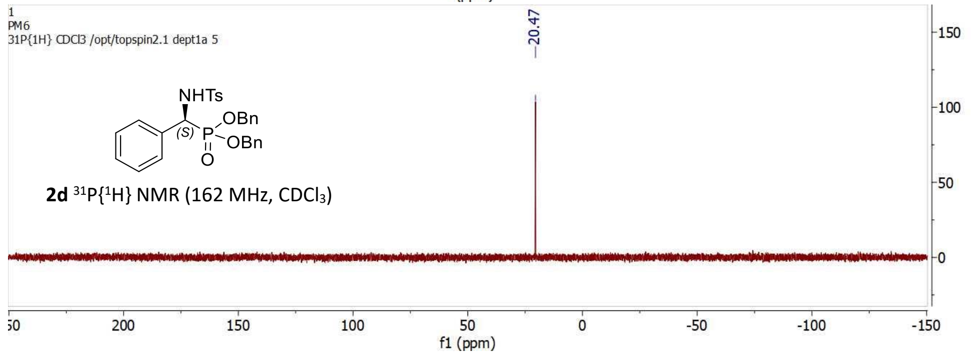
NHTs
(S)
OBn
OBn
O
2d ³¹P{¹H} NMR (162 MHz, CDCl₃)
f1 (ppm)

PPL-97 4-Me

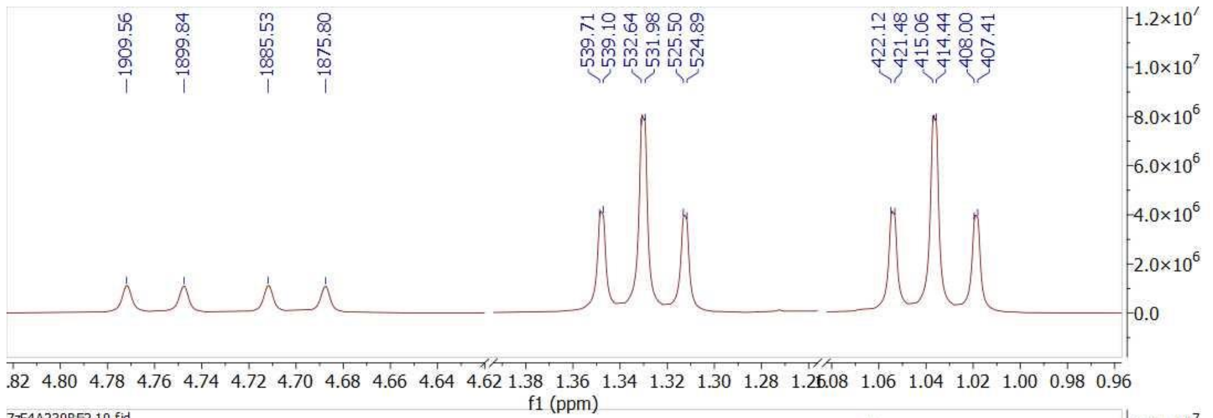
1909.56
1899.84
1885.53
1875.80
539.71
539.10
532.64
531.98
525.50
524.89
422.12
421.48
415.06
414.44
408.00
407.41
f1 (ppm)

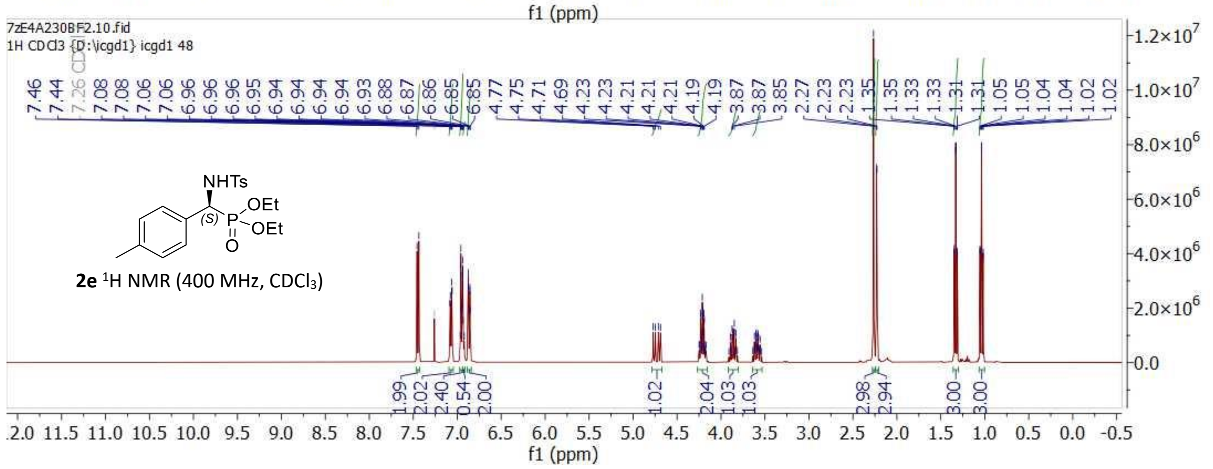
7zE4A230BF2.10.fid
1H CDCl3 {D:\icgd1} icgd1 48
NHTs
(S)
P
OEt
OEt
O
2e ^{1}H NMR (400 MHz, $CDCl_3$)
f1 (ppm)

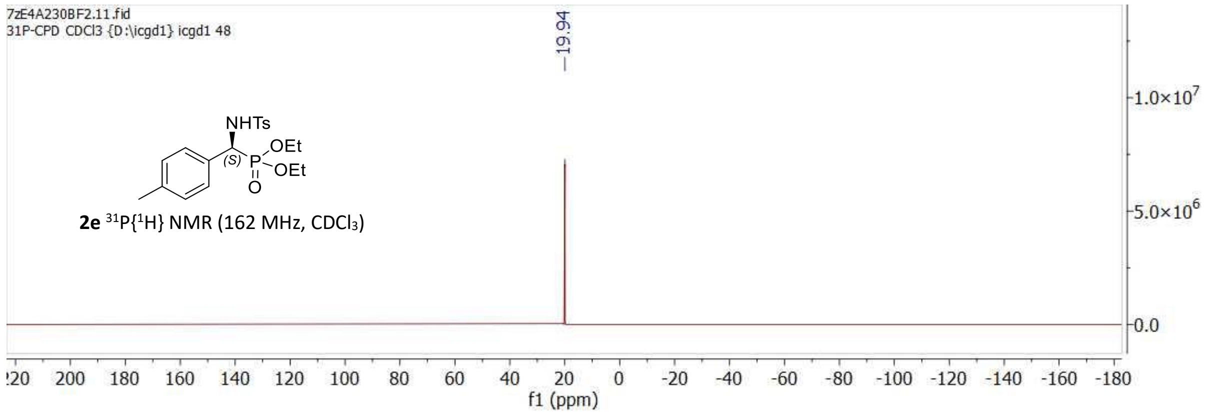
7zE4A230BF2.11.fid
31P-CPD CDCl3 {D:\icgd1} icgd1 48
19.94
NHTs
(S)
P
OEt
OEt
O
2e ^{31}P{^{1}H} NMR (162 MHz, $CDCl_3$)
f1 (ppm)

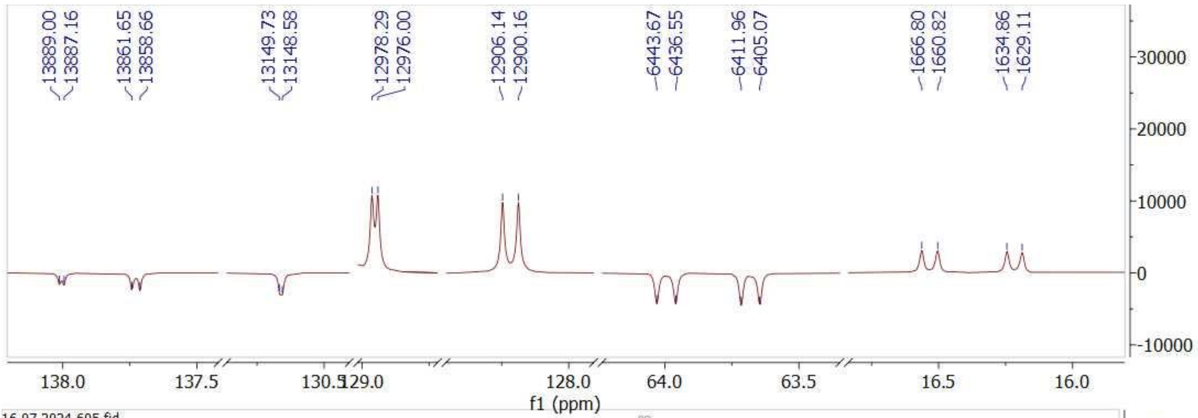

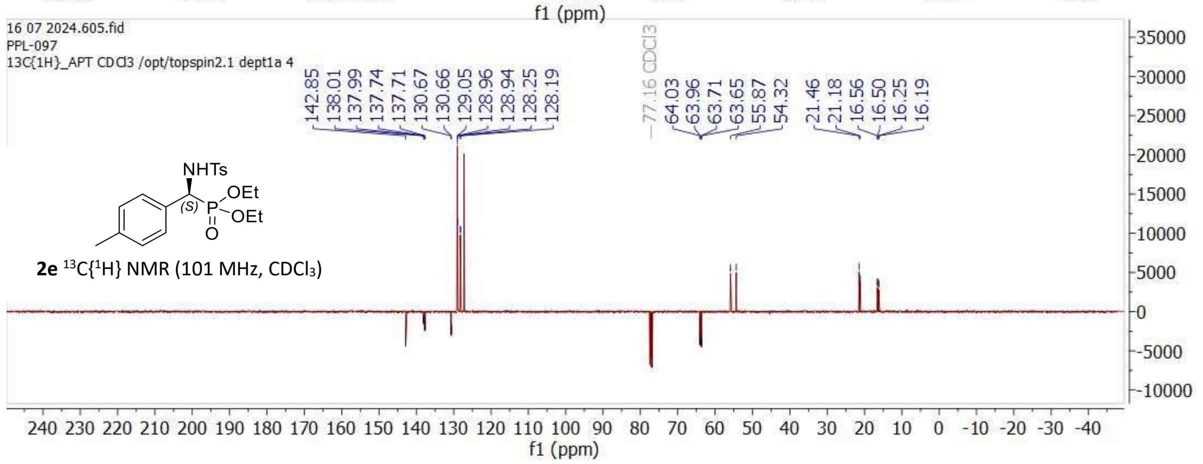
16 07 2024.605.fid
PPL-097
13C{1H}_APT CDCl3 /opt/topspin2.1 dept1a 4
NHTs
OEt
OEt
2e $^{13}C\{^{1}H\}$ NMR (101 MHz, $CDCl_3$)
f1 (ppm)

RC-073 3-Me

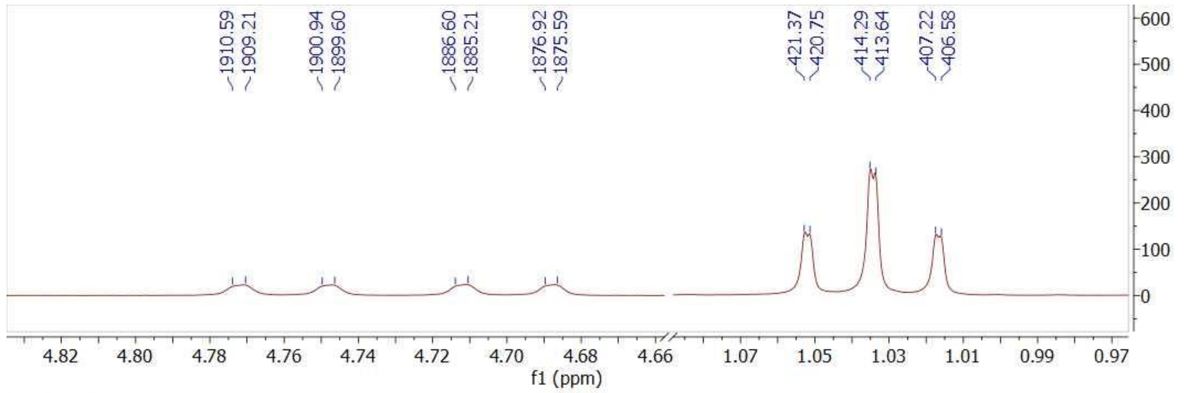

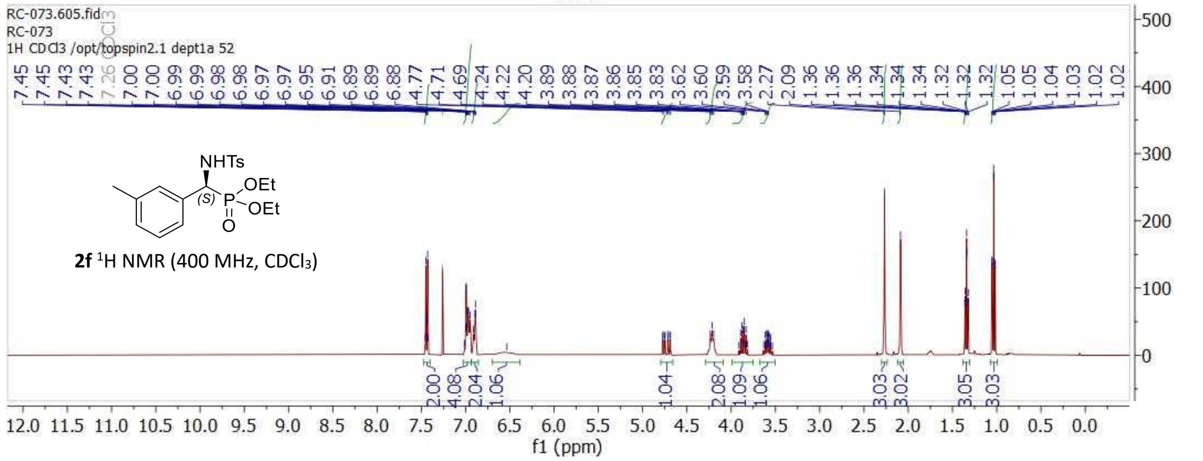
RC-073.605.fid
RC-073
1H CDCl3 /opt/topspin2.1 dept1a 52
NHTs
OEt
OEt
2f ^{1}H NMR (400 MHz, $CDCl_3$)
f1 (ppm)

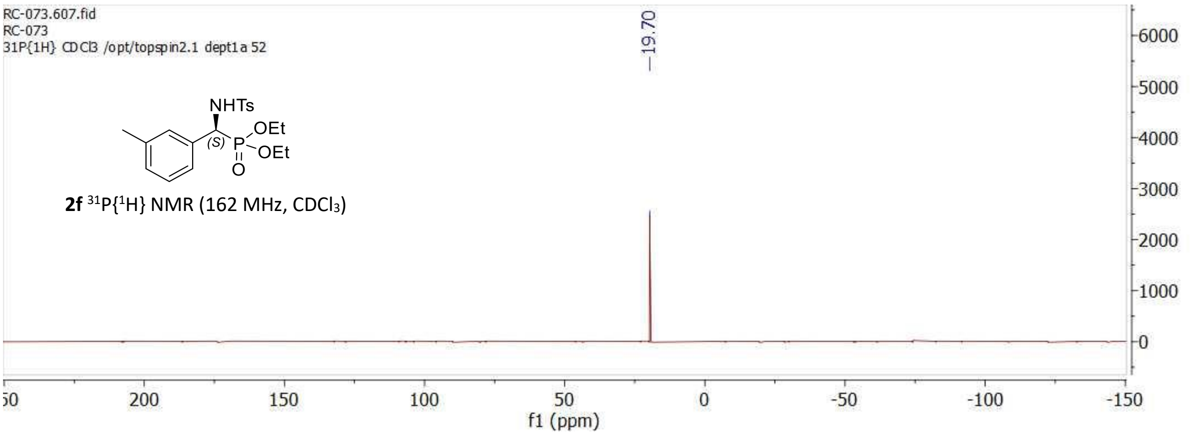
RC-073.607.fid
RC-073
31P{1H} CDCl3 /opt/topspin2.1 dept1a 52
19.70
NHTs
(S)
P
OEt
OEt
O
2f 31P{1H} NMR (162 MHz, CDCl3)
f1 (ppm)

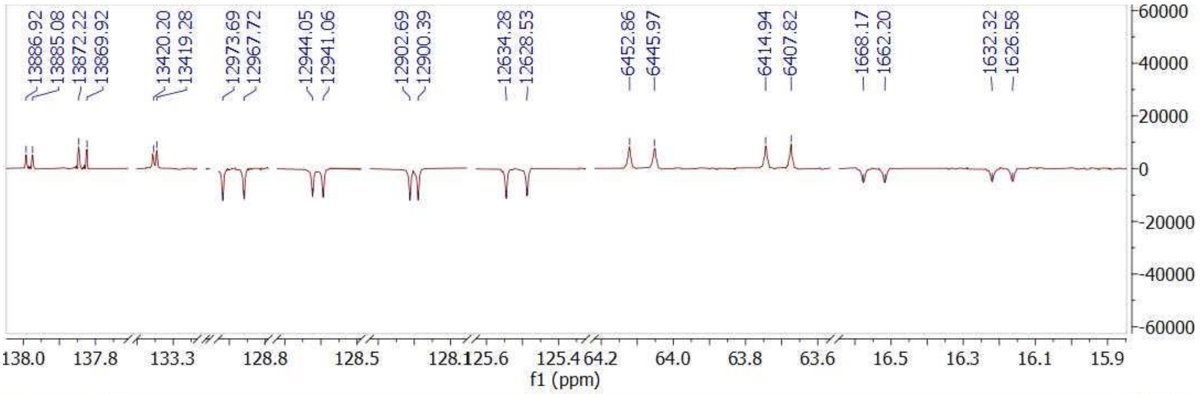
13886.92
13885.08
13872.22
13869.92
13420.20
13419.28
12973.69
12967.72
12944.05
12941.06
12902.69
12900.39
12634.28
12628.53
6452.86
6445.97
6414.94
6407.82
1668.17
1662.20
1632.32
1626.58
f1 (ppm)

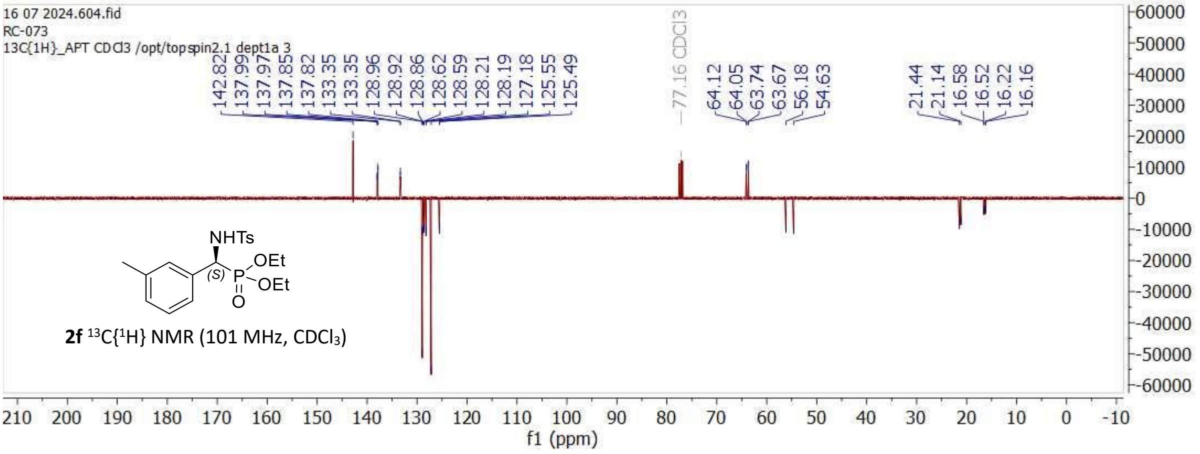
16 07 2024.604.fid
RC-073
13C{1H}_APT CDCl3 /opt/topspin2.1 dept1a 3
142.82
137.99
137.97
137.85
137.82
133.35
133.35
128.96
128.92
128.86
128.62
128.59
128.21
128.19
127.18
125.55
125.49
77.16 CDCl3
64.12
64.05
63.74
63.67
56.18
54.63
21.44
21.14
16.58
16.52
16.22
16.16
NHTs
(S)
P
OEt
OEt
O
2f 13C{1H} NMR (101 MHz, CDCl3)
f1 (ppm)

RC-071 4-F

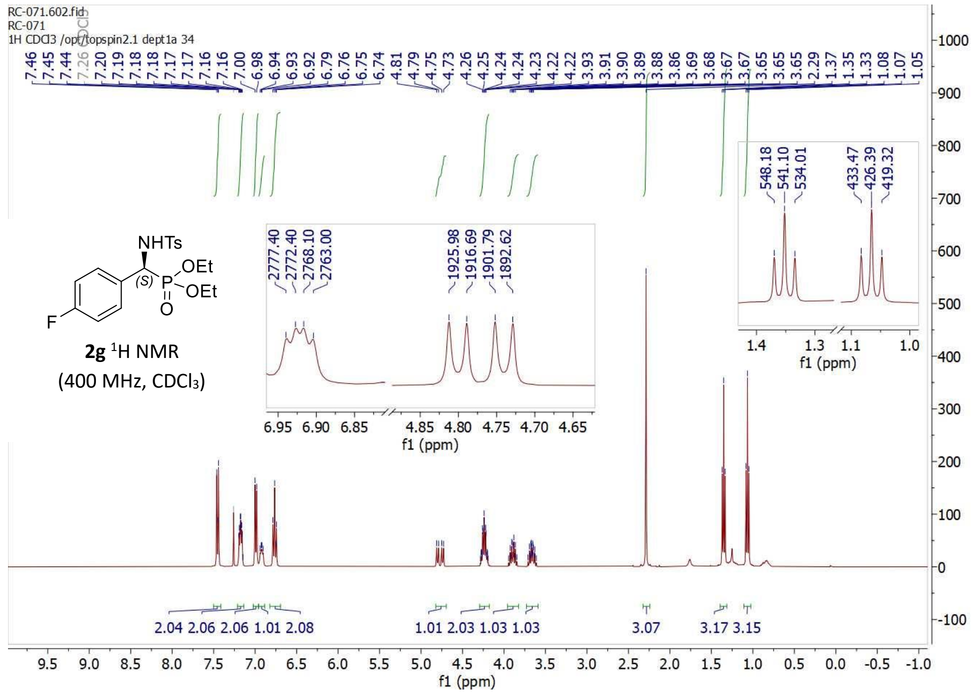

RC-071.602.fid
RC-071
1H CDCl3 /opt/topspin2.1 dept1a 34
NHTs
(S)
OEt
OEt
F
2g ¹H NMR
(400 MHz, CDCl₃)
f1 (ppm)

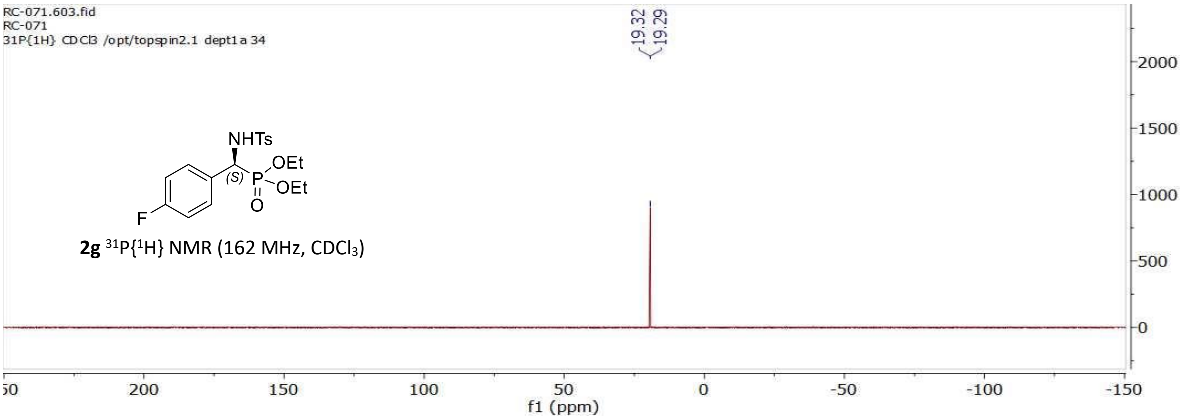

RC-071.603.fid
RC-071
31P{1H} CDCl3 /opt/topspin2.1 dept1a 34
19.32
19.29
NHTs
(S)
OEt
OEt
F
2g ³¹P{¹H} NMR (162 MHz, CDCl₃)
f1 (ppm)

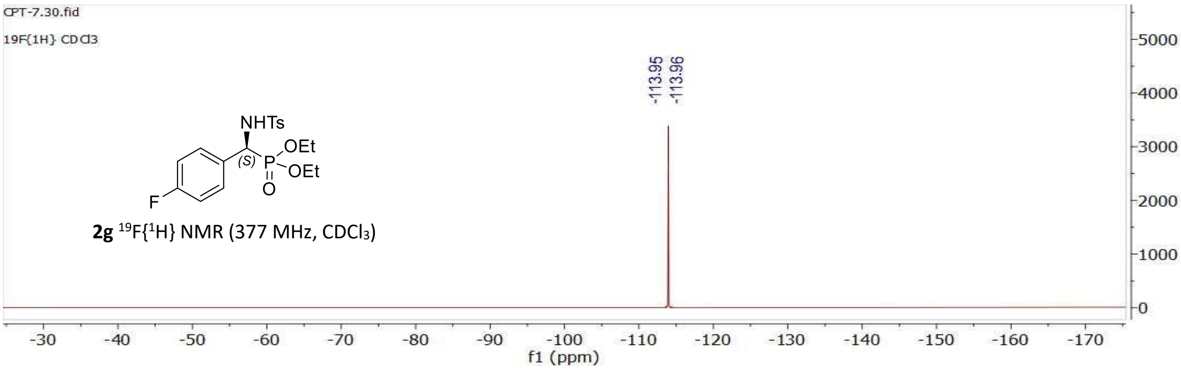

CPT-7.30.fid
19F{1H} CDCl3
-113.95
-113.96
NHTs
(S)
OEt
OEt
F
2g ¹⁹F{¹H} NMR (377 MHz, CDCl₃)
f1 (ppm)

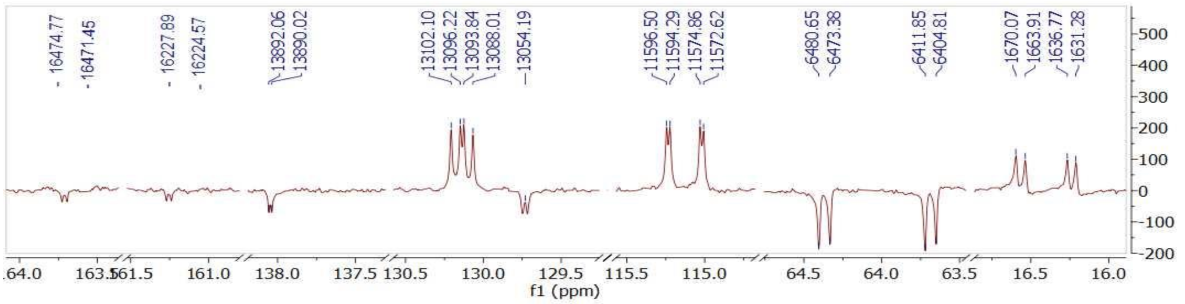
16474.77
16471.45
16227.89
16224.57
13892.06
13890.02
13102.10
13096.22
13093.84
13088.01
13054.19
11596.50
11594.29
11574.86
11572.62
6480.65
6473.38
6411.85
6404.81
1670.07
1663.91
1636.77
1631.28
f1 (ppm)

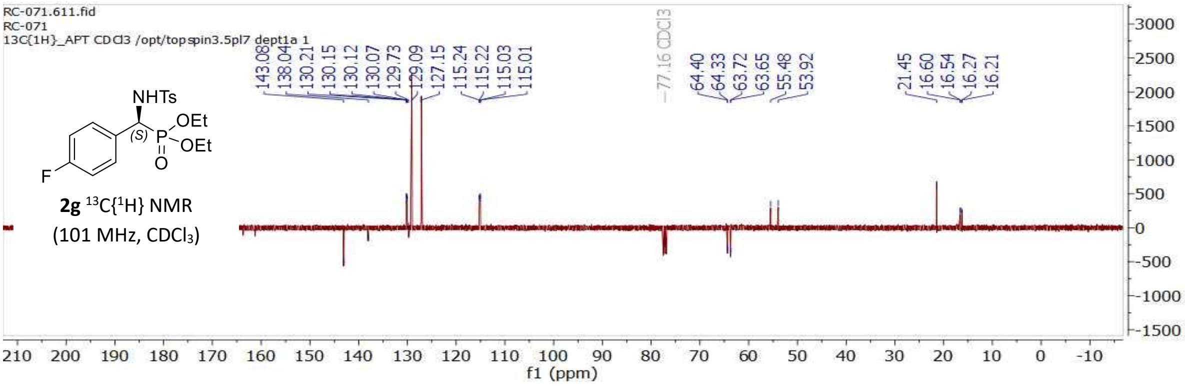
RC-071.611.fid
RC-071
13C{1H}_APT CDCl3 /opt/topspin3.5pl7 dept1a 1
143.08
138.04
130.21
130.15
130.12
130.07
129.73
129.09
127.15
115.24
115.22
115.03
115.01
77.16 CDCl3
64.40
64.33
63.72
63.65
55.48
53.92
21.45
16.60
16.54
16.27
16.21
NHTs
(S)
OEt
OEt
F
2g $^{13}C\{^1H\}$ NMR
(101 MHz, $CDCl_3$)
f1 (ppm)

RC-049 4-Br

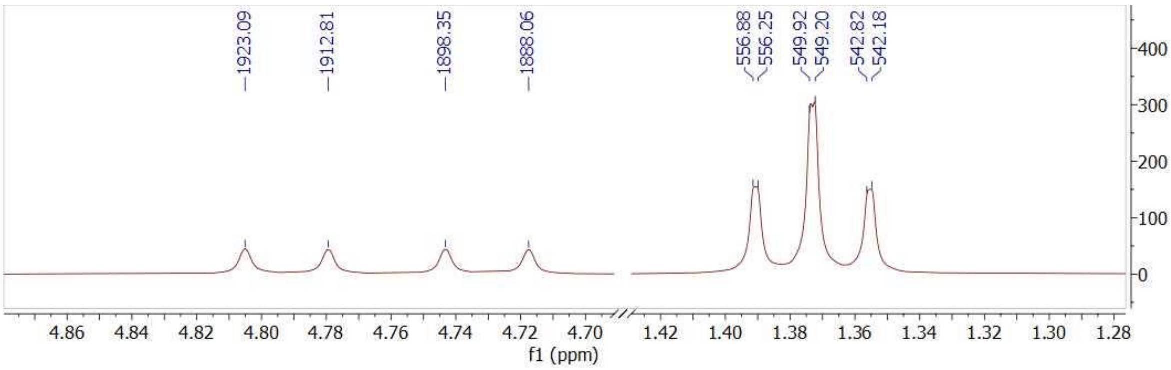

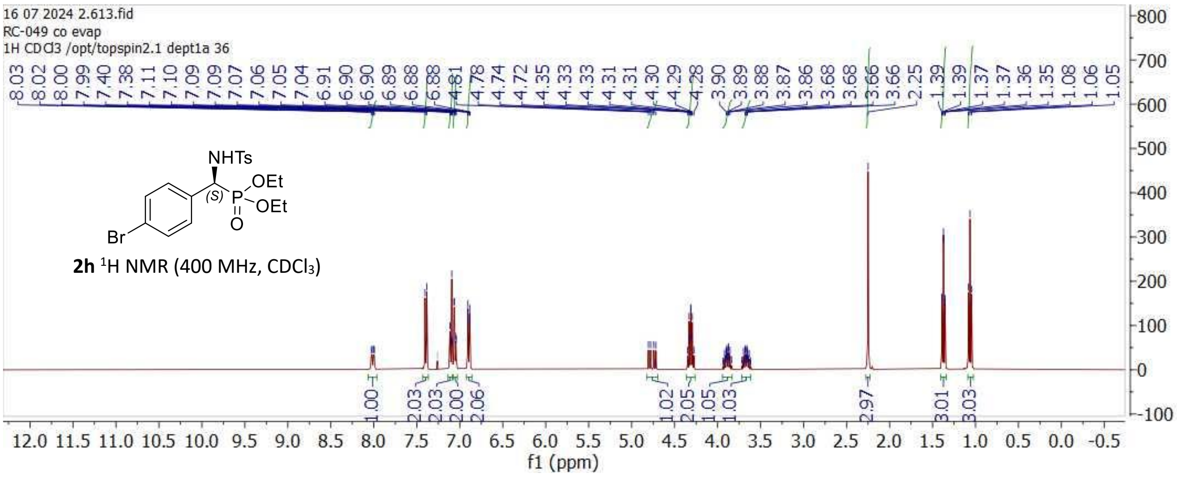

2h ^{1}H NMR (400 MHz, $CDCl_3$)

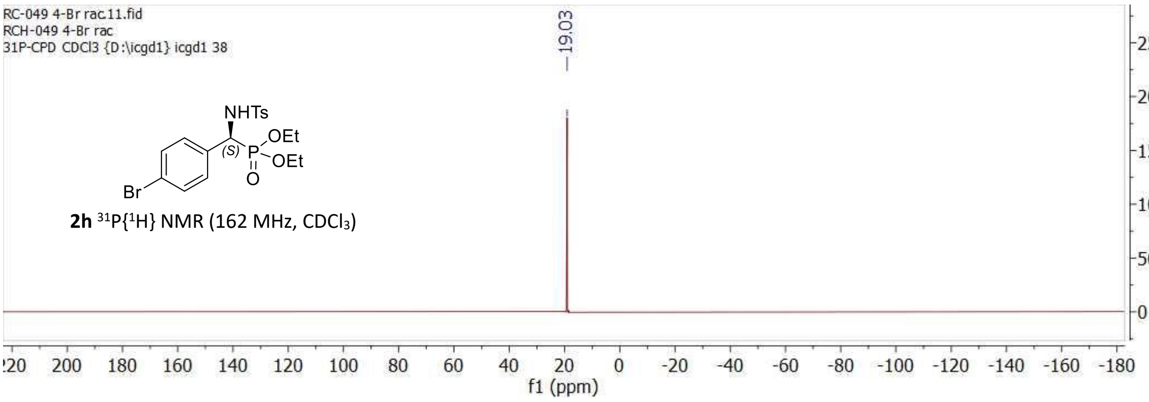

2h ^{31}P{^{1}H} NMR (162 MHz, $CDCl_3$)

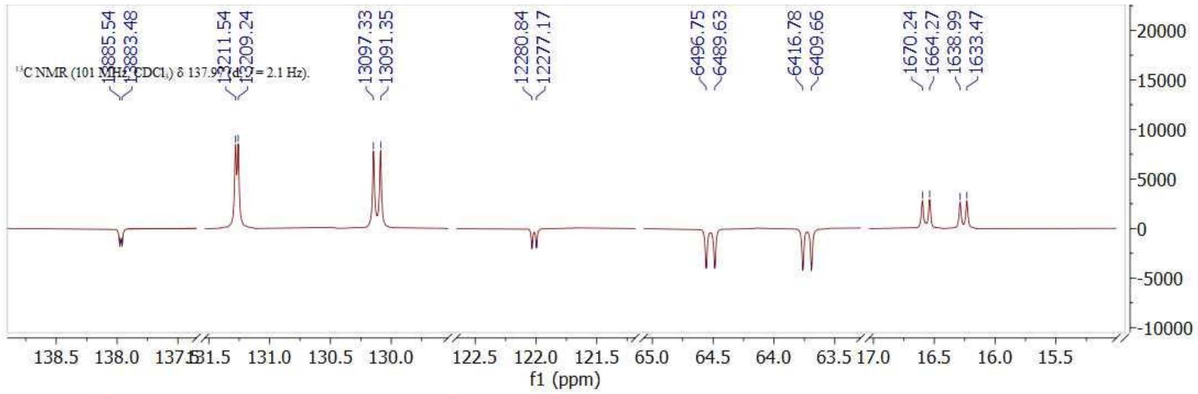

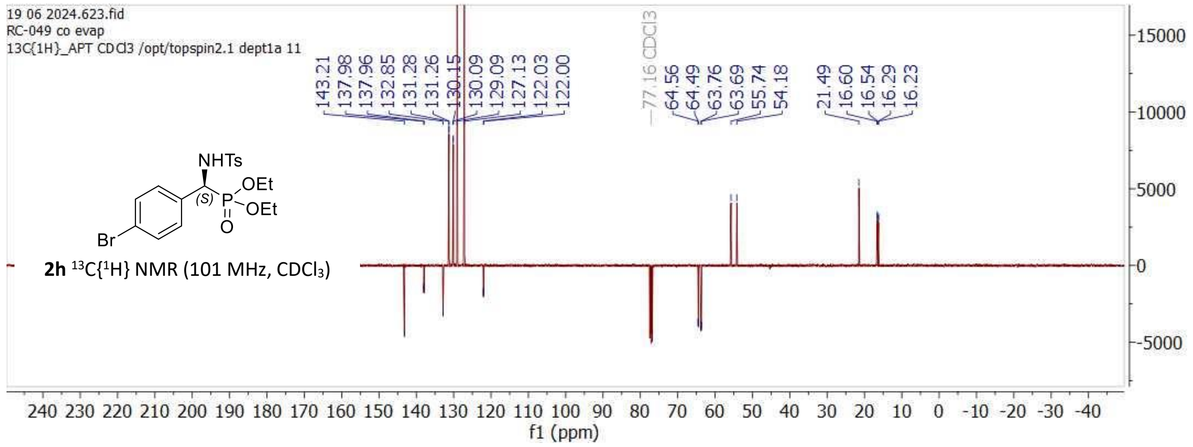

2h $^{13}C\{^{1}H\}$ NMR (101 MHz, $CDCl_3$)

RC-052 3-F

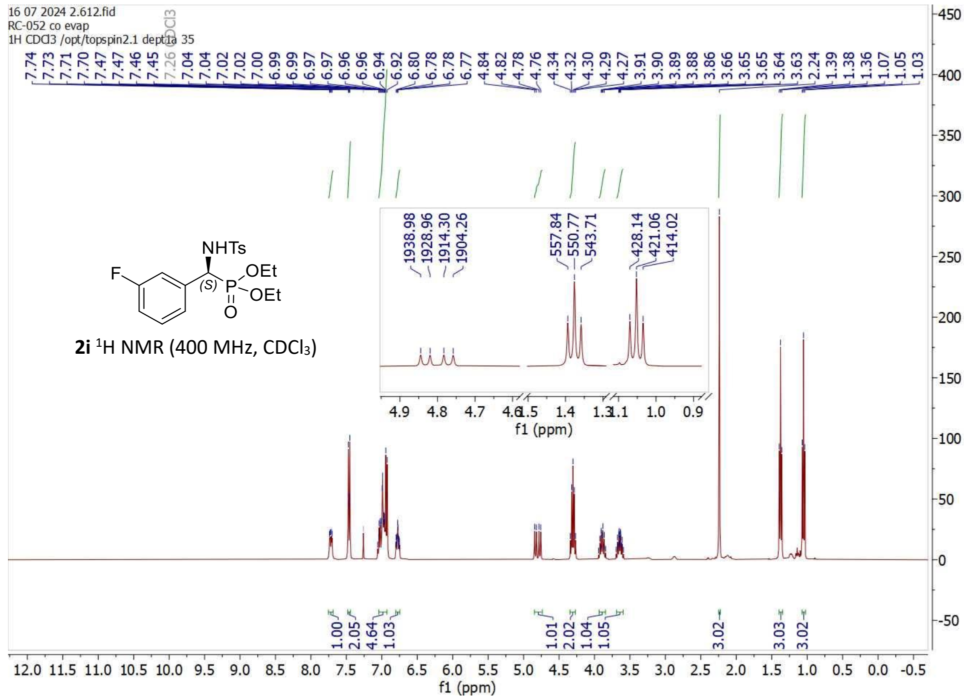
16 07 2024 2.612.fid
RC-052 co evap
1H CDCl3 /opt/topspin2.1 dept1a 35
7.74 7.73 7.71 7.70 7.47 7.47 7.46 7.45 7.26 CDCl3 7.04 7.04 7.02 7.02 7.00 6.99 6.99 6.97 6.97 6.96 6.96 6.94 6.92 6.80 6.78 6.78 6.77 4.84 4.82 4.78 4.76 4.34 4.32 4.30 4.29 4.27 3.91 3.90 3.89 3.88 3.86 3.66 3.65 3.65 3.64 3.63 2.24 1.39 1.38 1.36 1.07 1.05 1.03
1938.98 1928.96 1914.30 1904.26
557.84 550.77 543.71
428.14 421.06 414.02
NHTs
F
(S)
P
OEt
OEt
O
2i ¹H NMR (400 MHz, CDCl₃)
1.00 2.05 4.64 1.03 1.01 2.02 1.04 1.05 3.02 3.03 3.02
f1 (ppm)

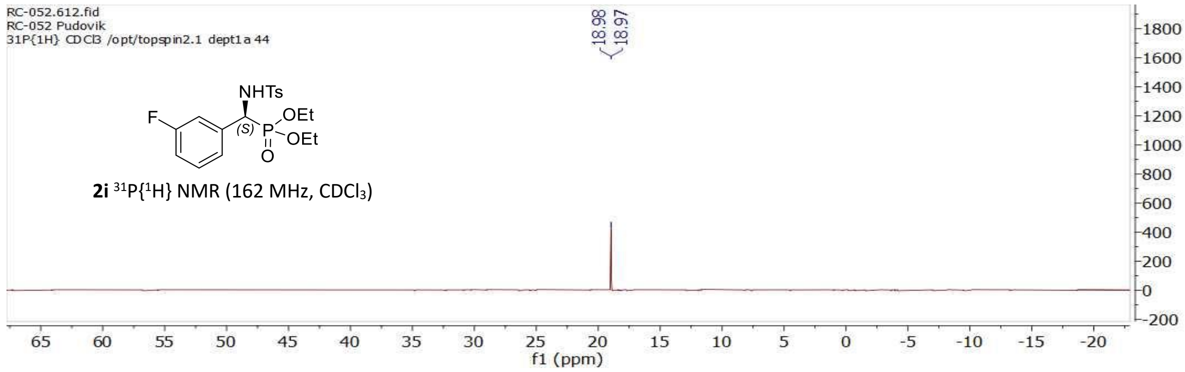
RC-052.612.fid
RC-052 Pudovik
31P{1H} CDCl3 /opt/topspin2.1 dept1a 44
18.98
18.97
NHTs
F
(S)
P
OEt
OEt
O
2i ³¹P{¹H} NMR (162 MHz, CDCl₃)
f1 (ppm)

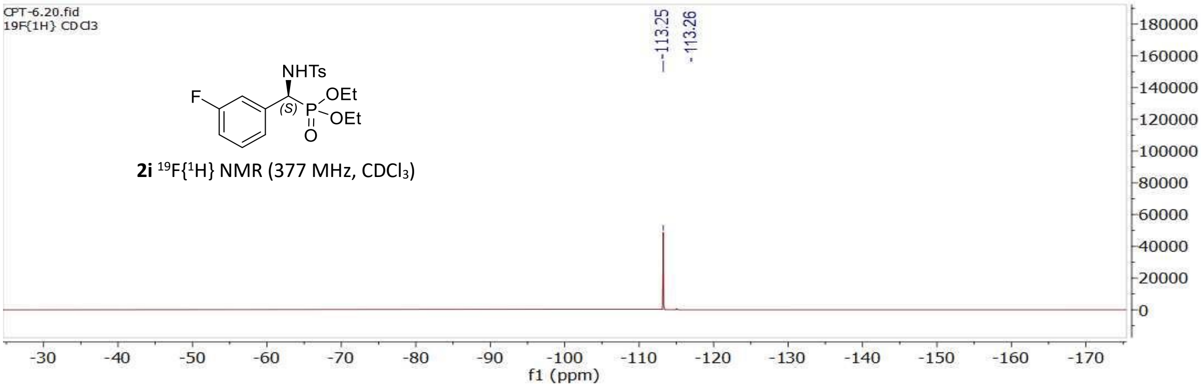
CPT-6.20.fid
19F{1H} CDCl3
NHTs
F
(S)
P
OEt
OEt
O
2i 19F{1H} NMR (377 MHz, CDCl3)
-113.25
-113.26
f1 (ppm)

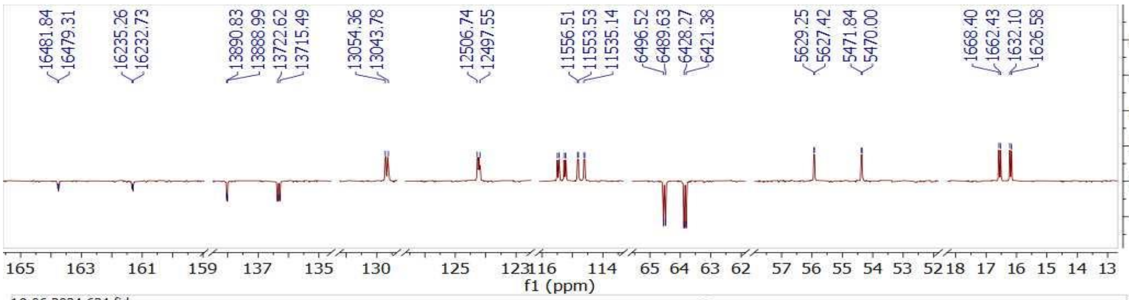
16481.84
16479.31
16235.26
16232.73
13890.83
13888.99
13722.62
13715.49
13054.36
13043.78
12506.74
12497.55
11556.51
11553.53
11535.14
6496.52
6489.63
6428.27
6421.38
5629.25
5627.42
5471.84
5470.00
1668.40
1662.43
1632.10
1626.58
f1 (ppm)

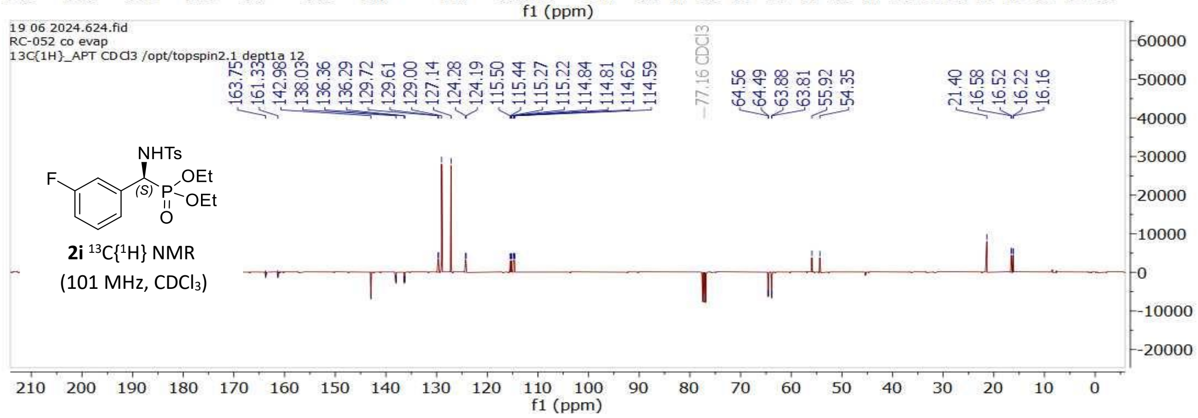
19 06 2024.624.fid
RC-052 co evap
13C{1H}_APT CDCl3 /opt/topspin2.1 dept1a 12
163.75
161.33
142.98
138.03
136.36
136.29
129.72
129.61
129.00
127.14
124.28
124.19
115.50
115.44
115.27
115.22
114.84
114.81
114.62
114.59
77.16 CDCl3
64.56
64.49
63.88
63.81
55.92
54.35
21.40
16.58
16.52
16.22
16.16
NHTs
F
(S)
P
OEt
OEt
O
2i 13C{1H} NMR
(101 MHz, CDCl3)
f1 (ppm)

RC-072 2-F

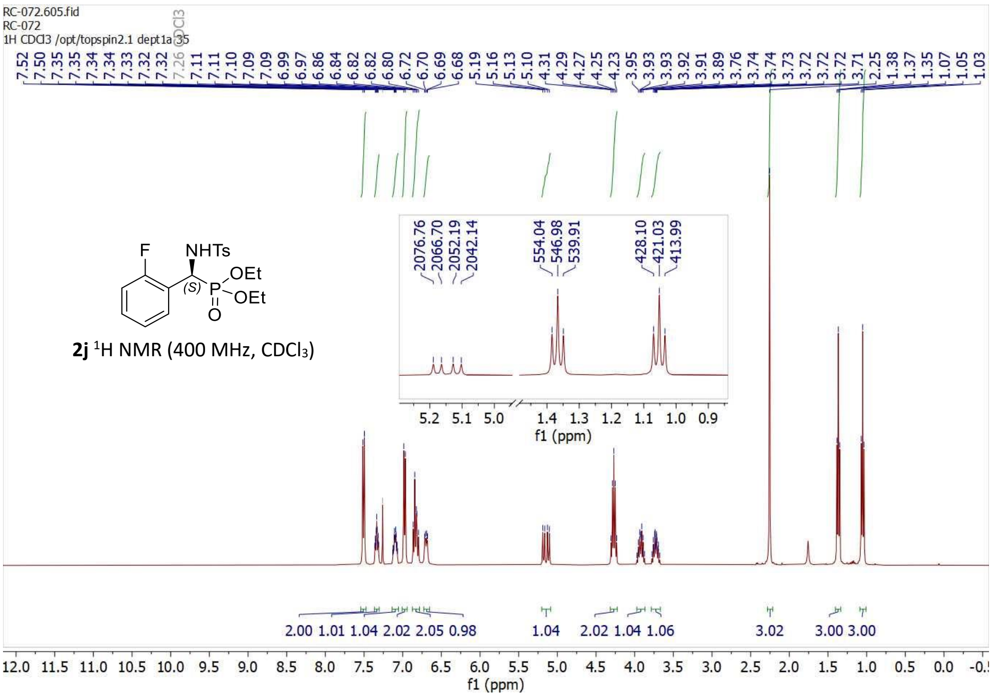
RC-072.605.fid
RC-072
1H CDCl3 /opt/topspin2.1 dept1a 35
7.52 7.50 7.35 7.35 7.34 7.33 7.32 7.32 7.26 CDCl3 7.11 7.11 7.10 7.09 7.09 6.99 6.97 6.86 6.84 6.82 6.82 6.80 6.72 6.70 6.69 6.68 5.19 5.16 5.13 5.10 4.31 4.29 4.27 4.25 4.23 3.95 3.93 3.93 3.92 3.91 3.89 3.76 3.74 3.74 3.73 3.72 3.72 3.72 3.71 2.25 1.38 1.37 1.35 1.07 1.05 1.03
F
NHTs
(S)
P
OEt
OEt
O
2j 1H NMR (400 MHz, CDCl3)
2076.76 2066.70 2052.19 2042.14
554.04 546.98 539.91
428.10 421.03 413.99
5.2 5.1 5.0 1.4 1.3 1.2 1.1 1.0 0.9
f1 (ppm)
2.00 1.01 1.04 2.02 2.05 0.98 1.04 2.02 1.04 1.06 3.02 3.00 3.00
12.0 11.5 11.0 10.5 10.0 9.5 9.0 8.5 8.0 7.5 7.0 6.5 6.0 5.5 5.0 4.5 4.0 3.5 3.0 2.5 2.0 1.5 1.0 0.5 0.0
f1 (ppm)

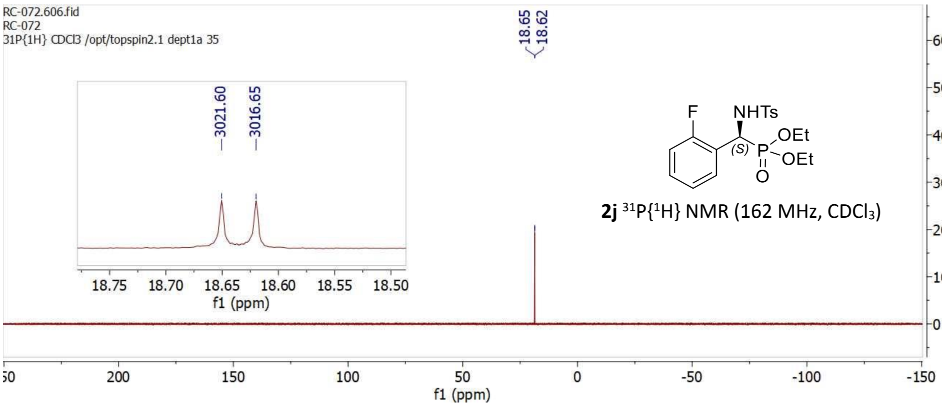
RC-072.606.fid
RC-072
31P{1H} CDCl3 /opt/topspin2.1 dept1a 35
18.65
18.62
3021.60
3016.65
18.75 18.70 18.65 18.60 18.55 18.50
f1 (ppm)
F
NHTs
(S)
P
OEt
OEt
O
2j 31P{1H} NMR (162 MHz, CDCl3)
6000 5000 4000 3000 2000 1000 0
200 150 100 50 0 -50 -100 -150
f1 (ppm)

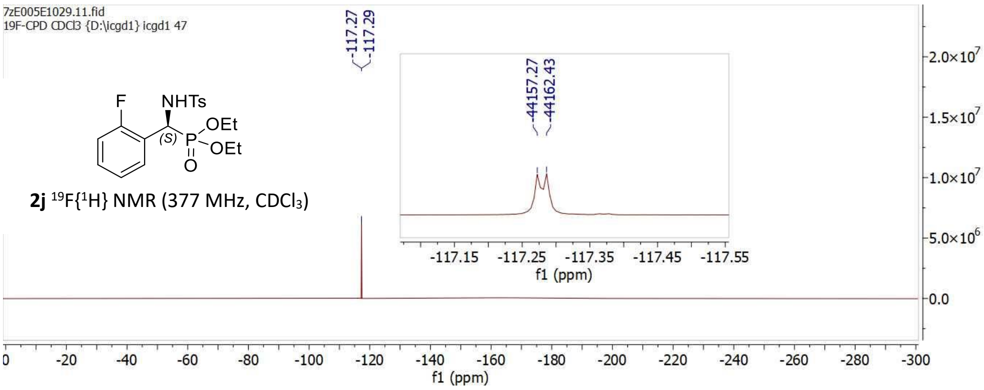
7zE005E1029.11.fid
19F-CPD CDCl3 {D:\icgd1} icgd1 47
-117.27
-117.29
F
NHTs
(S)
P
OEt
OEt
O
2j 19F{1H} NMR (377 MHz, CDCl3)
-44157.27
-44162.43
-117.15 -117.25 -117.35 -117.45 -117.55
f1 (ppm)
2.0×10^7 1.5×10^7 1.0×10^7 5.0×10^6 0.0
-20 -40 -60 -80 -100 -120 -140 -160 -180 -200 -220 -240 -260 -280 -300
f1 (ppm)

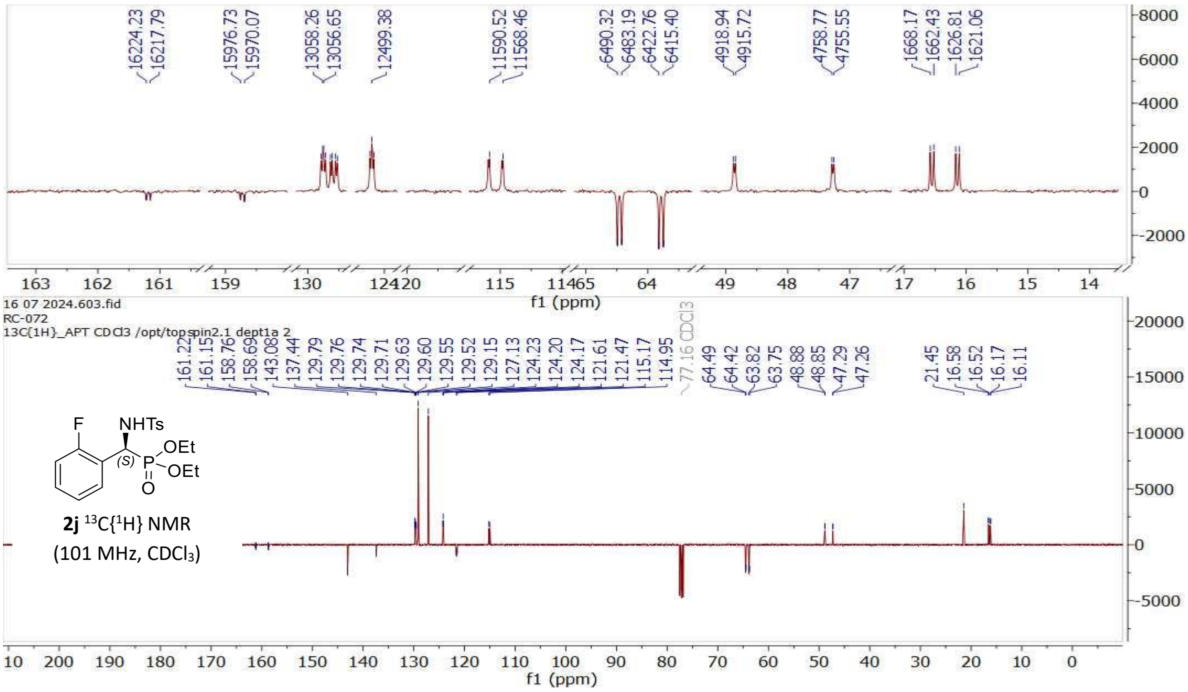
16 07 2024.603.fid
RC-072
13C{1H}_APT CDCl3 /opt/topspin2.1 dept1a 2
NHTs
OEt
OEt
2j 13C{1H} NMR
(101 MHz, CDCl3)
f1 (ppm)

RC-063 4-CN

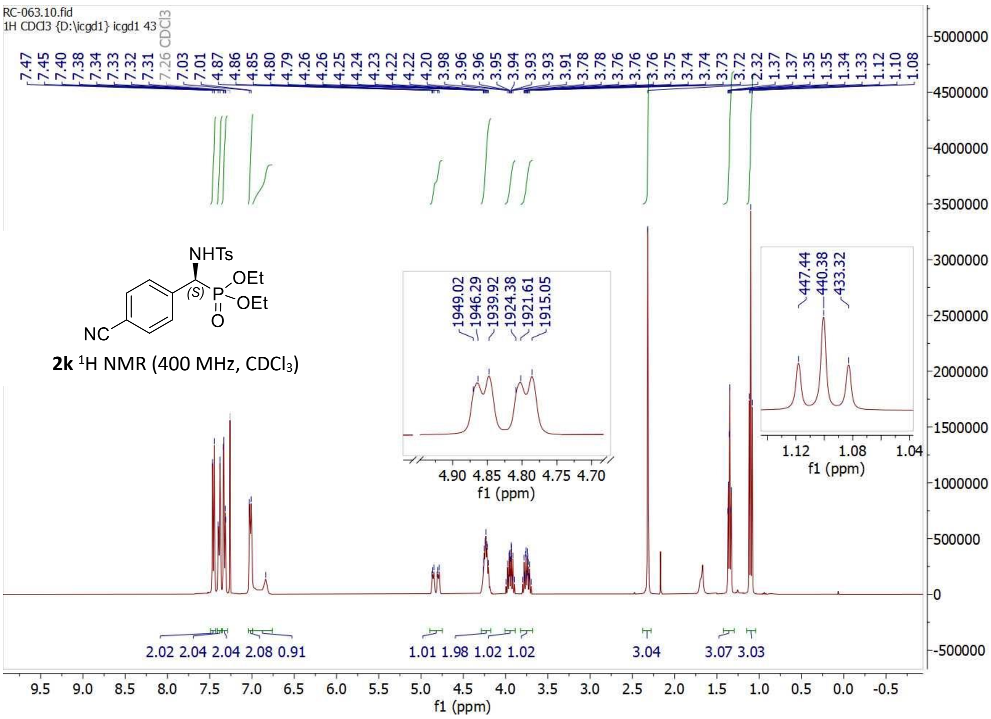
RC-063.10.fid
1H CDCl3 {D:\icgd1} icgd1 43
NHTs
OEt
OEt
NC
2k 1H NMR (400 MHz, CDCl3)
f1 (ppm)

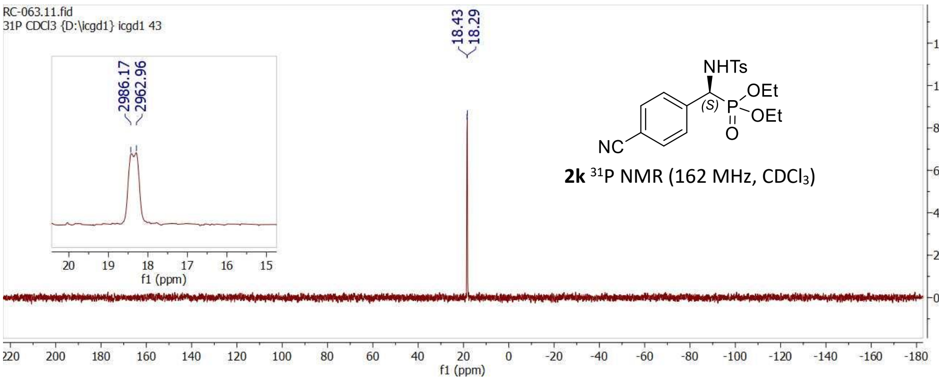
RC-063.11.fid
31P CDCl3 {D:\icgd1} icgd1 43
NHTs
OEt
OEt
NC
2k 31P NMR (162 MHz, CDCl3)
f1 (ppm)

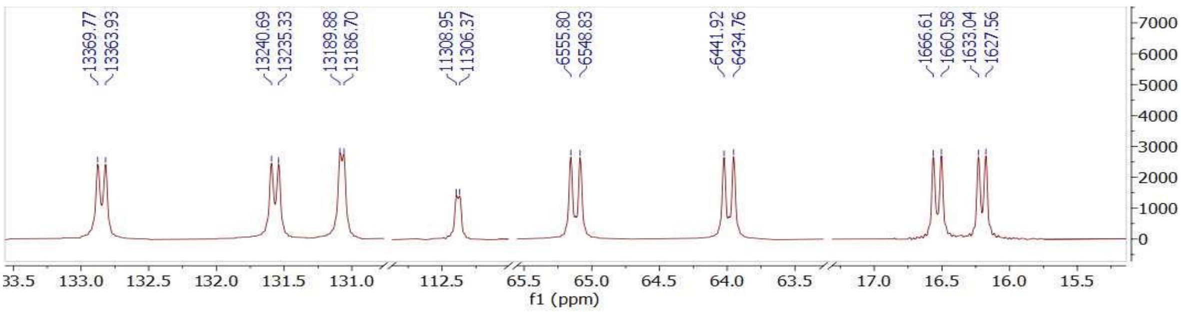
13369.77
13363.93
13240.69
13235.33
13189.88
13186.70
11308.95
11306.37
6555.80
6548.83
6441.92
6434.76
1666.61
1660.58
1633.04
1627.56
f1 (ppm)

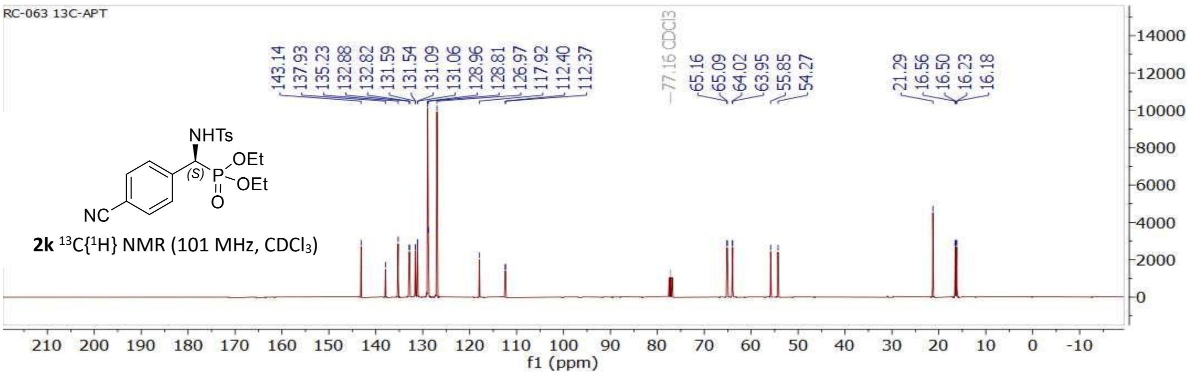
RC-063 13C-APT
143.14
137.93
135.23
132.88
132.82
131.59
131.54
131.09
131.06
128.96
128.81
126.97
117.92
112.40
112.37
77.16 CDCl3
65.16
65.09
64.02
63.95
55.85
54.27
21.29
16.56
16.50
16.23
16.18
NHTs
OEt
OEt
(S)
P
O
NC
2k $^{13}C\{^1H\}$ NMR (101 MHz, $CDCl_3$)
f1 (ppm)

RC-064 3-CN

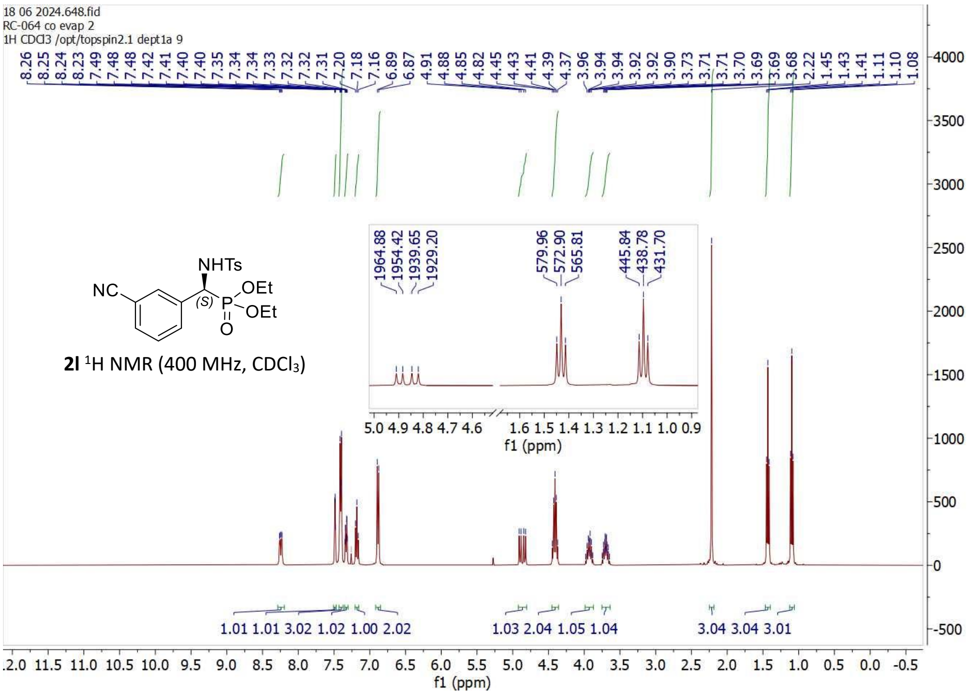
18 06 2024.648.fid
RC-064 co evap 2
1H CDCl3 /opt/topspin2.1 dept1a 9
8.26
8.25
8.24
8.23
7.49
7.48
7.48
7.42
7.41
7.40
7.40
7.35
7.34
7.34
7.33
7.32
7.32
7.31
7.20
7.18
7.16
6.89
6.87
4.91
4.88
4.85
4.82
4.45
4.43
4.41
4.39
4.37
3.96
3.94
3.94
3.92
3.92
3.90
3.73
3.71
3.70
3.69
3.69
3.68
2.22
1.45
1.43
1.41
1.11
1.10
1.08
1964.88
1954.42
1939.65
1929.20
579.96
572.90
565.81
445.84
438.78
431.70
NC
NHTs
(S)
P
OEt
OEt
O
2l ^1H NMR (400 MHz, $CDCl_3$)
1.01 1.01 3.02 1.02 1.00 2.02
1.03 2.04 1.05 1.04
3.04 3.04 3.01
f1 (ppm)

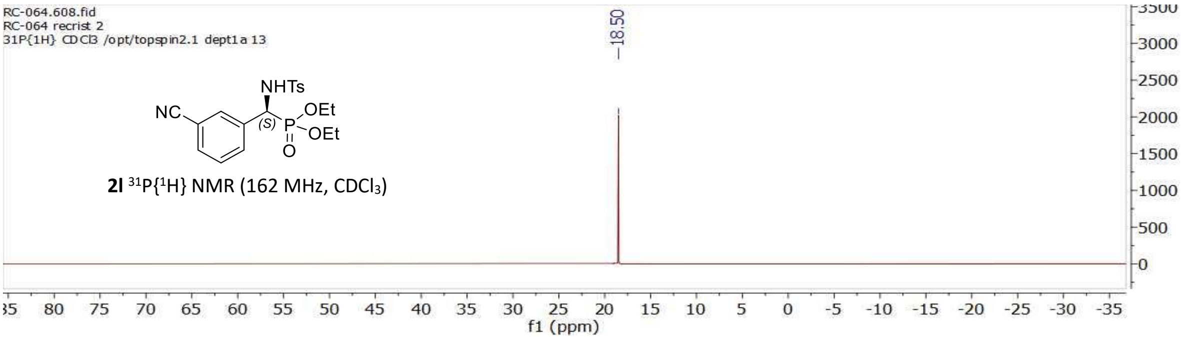
RC-064.608.fid
RC-064 recrist 2
31P{1H} CDCl3 /opt/topspin2.1 dept1a 13
NHTs
NC
(S)
OEt
OEt
2l 31P{1H} NMR (162 MHz, CDCl3)
f1 (ppm)

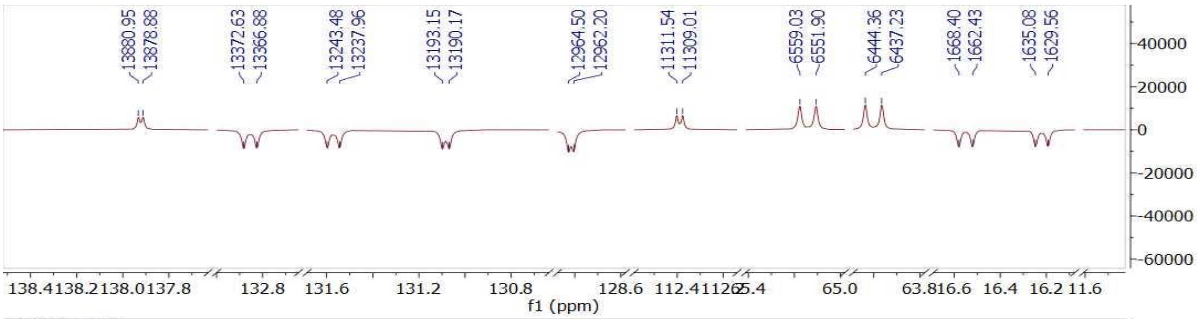
f1 (ppm)

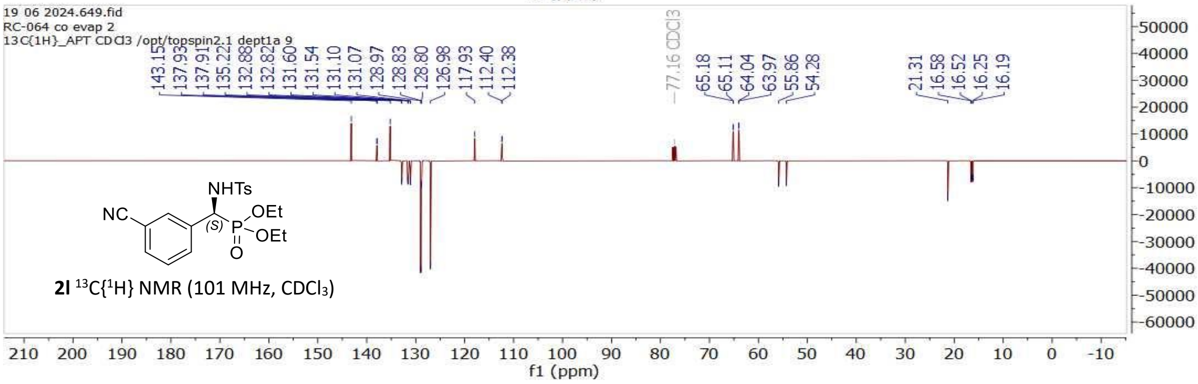
19 06 2024.649.fid
RC-064 co evap 2
13C{1H}_APT CDCl3 /opt/topspin2.1 dept1a 9
NHTs
NC
(S)
OEt
OEt
2l 13C{1H} NMR (101 MHz, CDCl3)
f1 (ppm)

RC-053 4-NO2

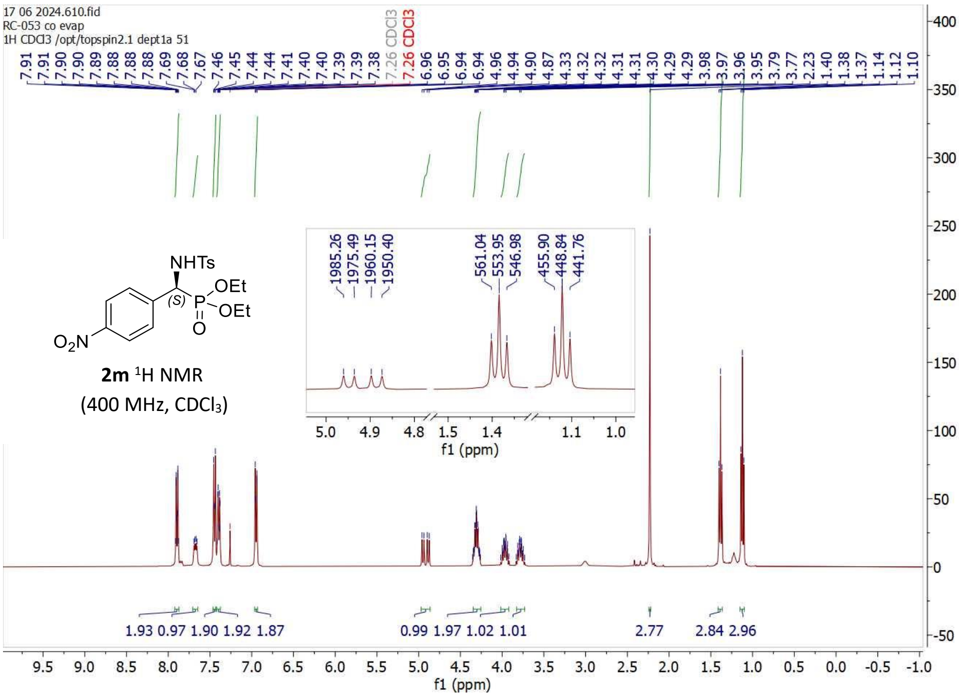
17 06 2024.610.fid
RC-053 co evap
1H CDCl3 /opt/topspin2.1 dept1a 51
NHTs
(S)
OEt
OEt
O2N
2m 1H NMR
(400 MHz, CDCl3)
f1 (ppm)

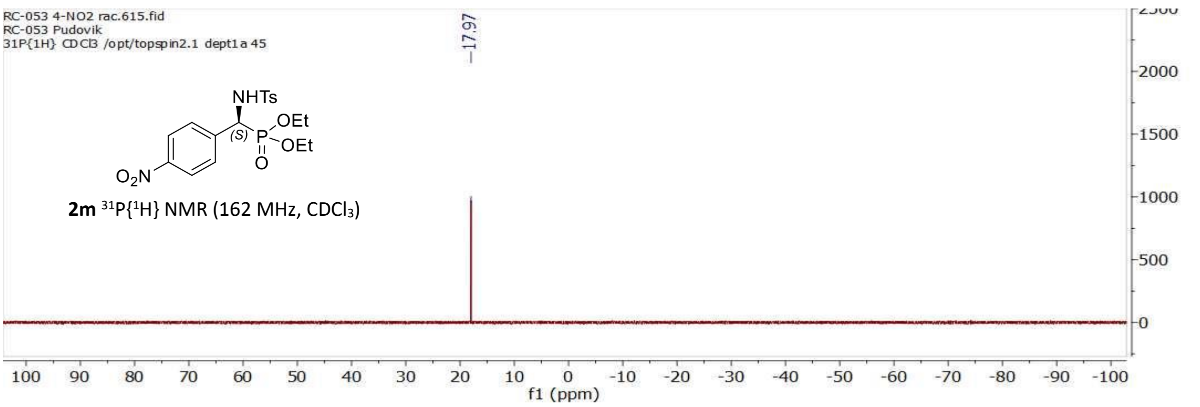
RC-053 4-NO2 rac.615.fid
RC-053 Pudovik
31P{1H} CDCl3 /opt/topspin2.1 dept1a 45
17.97
NHTs
(S)
P
OEt
OEt
O
O_2N
2m $^{31}P\{^1H\}$ NMR (162 MHz, $CDCl_3$)
f1 (ppm)

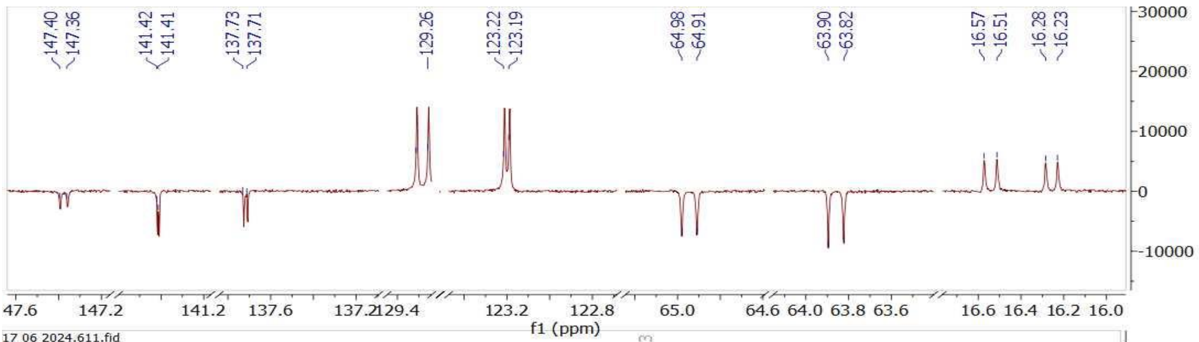
147.40
147.36
141.42
141.41
137.73
137.71
129.26
123.22
123.19
64.98
64.91
63.90
63.82
16.57
16.51
16.28
16.23
f1 (ppm)

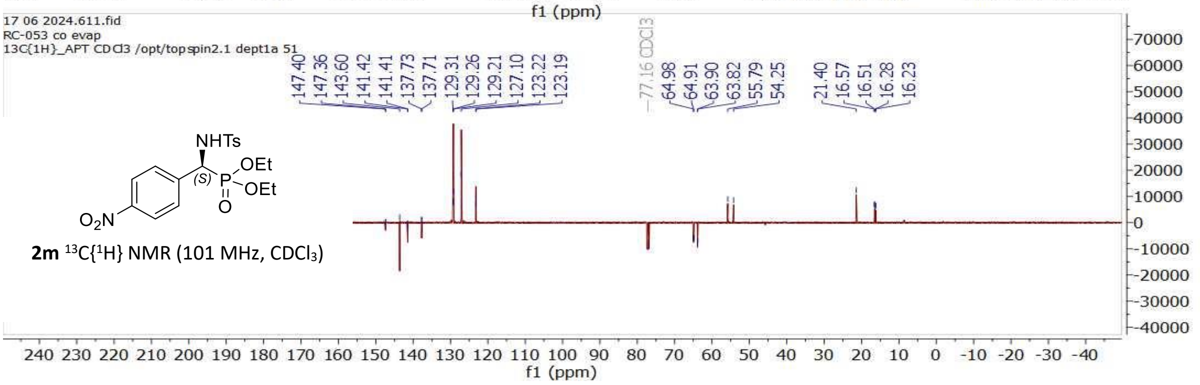
17 06 2024.611.fid
RC-053 co evap
13C{1H}_APT CDCl3 /opt/topspin2.1 dept1a 51
147.40
147.36
143.60
141.42
141.41
137.73
137.71
129.31
129.26
129.21
127.10
123.22
123.19
77.16 CDCl3
64.98
64.91
63.90
63.82
55.79
54.25
21.40
16.57
16.51
16.28
16.23
NHTs
(S)
P
OEt
OEt
O
O_2N
2m $^{13}C\{^1H\}$ NMR (101 MHz, $CDCl_3$)
f1 (ppm)

RC-047 3-NO2

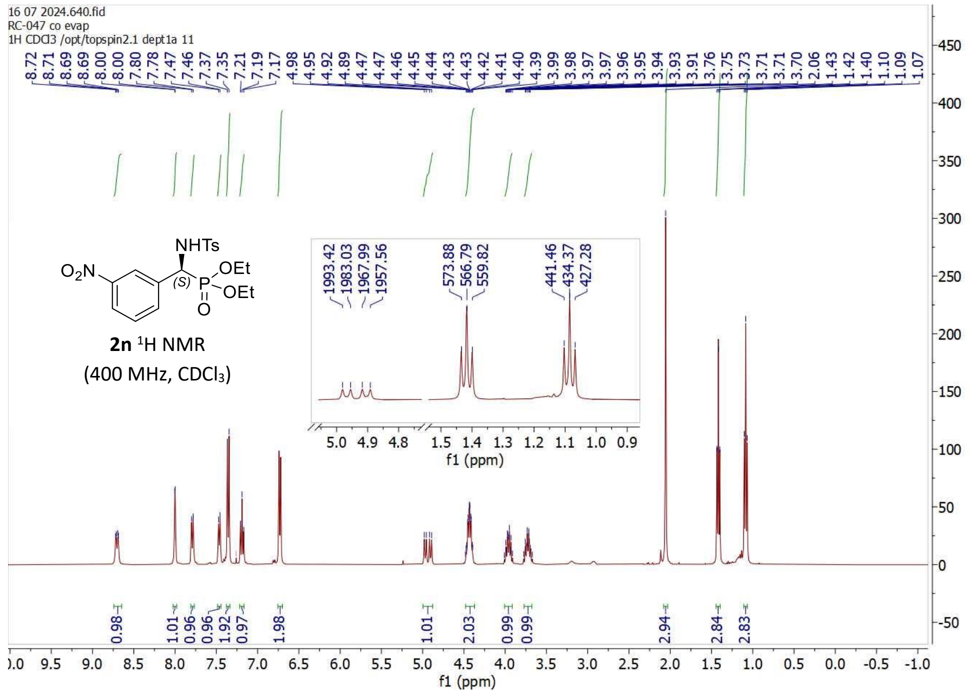
16 07 2024.640.fid
RC-047 co evap
1H CDCl3 /opt/topspin2.1 dept1a 11
NHTs
O_2N
(S)
P
OEt
OEt
O
2n ^1H NMR
(400 MHz, $CDCl_3$)
f1 (ppm)

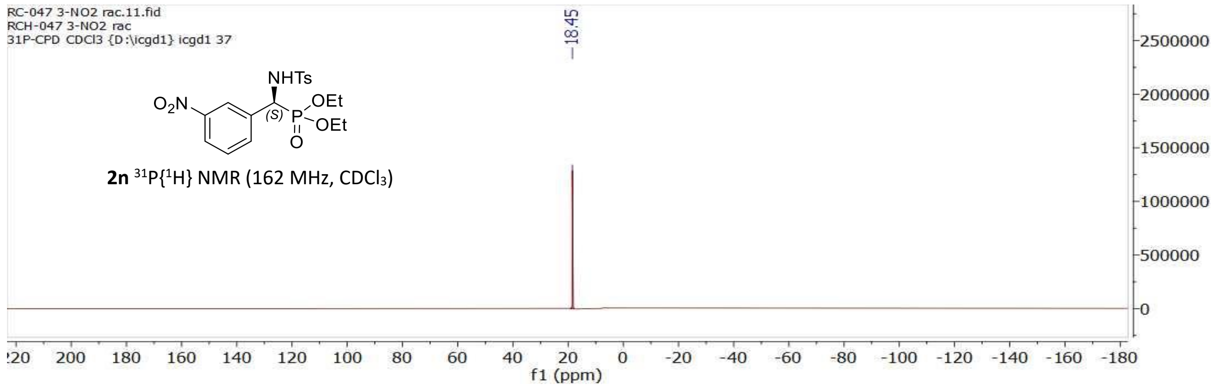
RC-047 3-NO2 rac.11.fid
RCH-047 3-NO2 rac
31P-CPD CDCl3 {D:\icgd1} icgd1 37
18.45
NHTs
O_2N
(S)
P
OEt
OEt
O
2n $^{31}P\{^1H\}$ NMR (162 MHz, $CDCl_3$)
f1 (ppm)

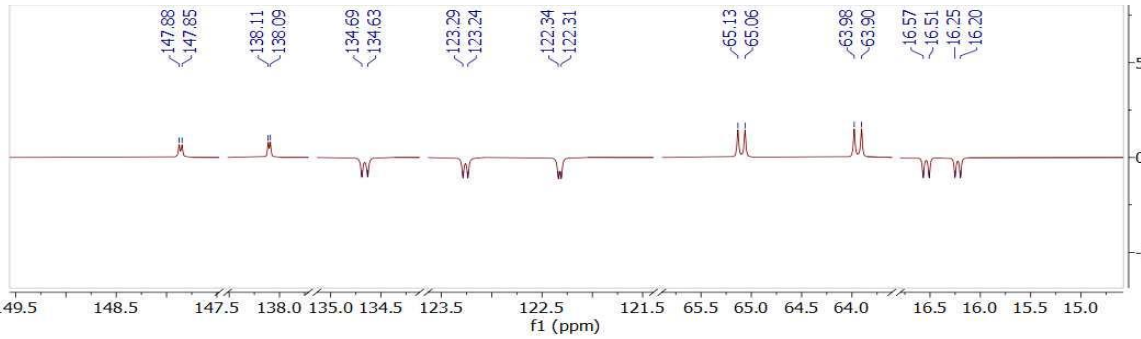
f1 (ppm)

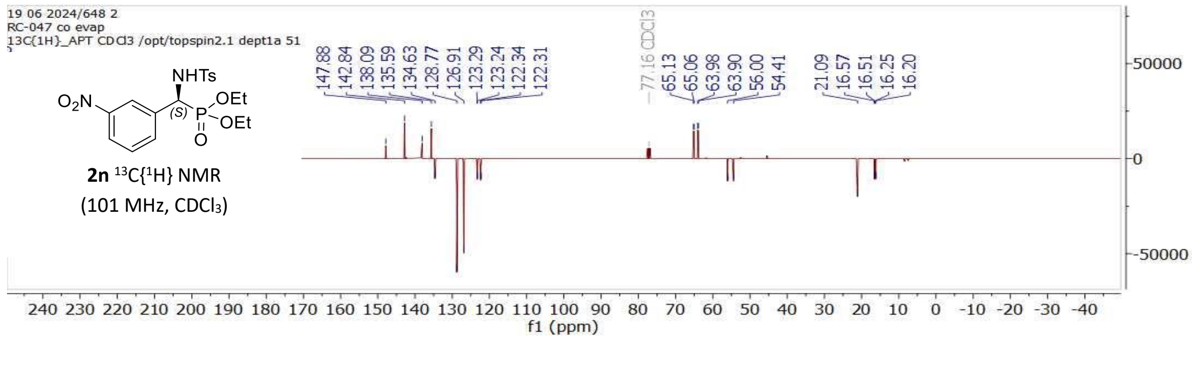
19 06 2024/648 2
RC-047 co evap
13C{1H}_APT CDCl3 /opt/topspin2.1 dept1a 51
77.16 CDCl3
NHTs
O_2N
(S)
P
OEt
OEt
O
2n $^{13}C\{^1H\}$ NMR
(101 MHz, $CDCl_3$)
f1 (ppm)

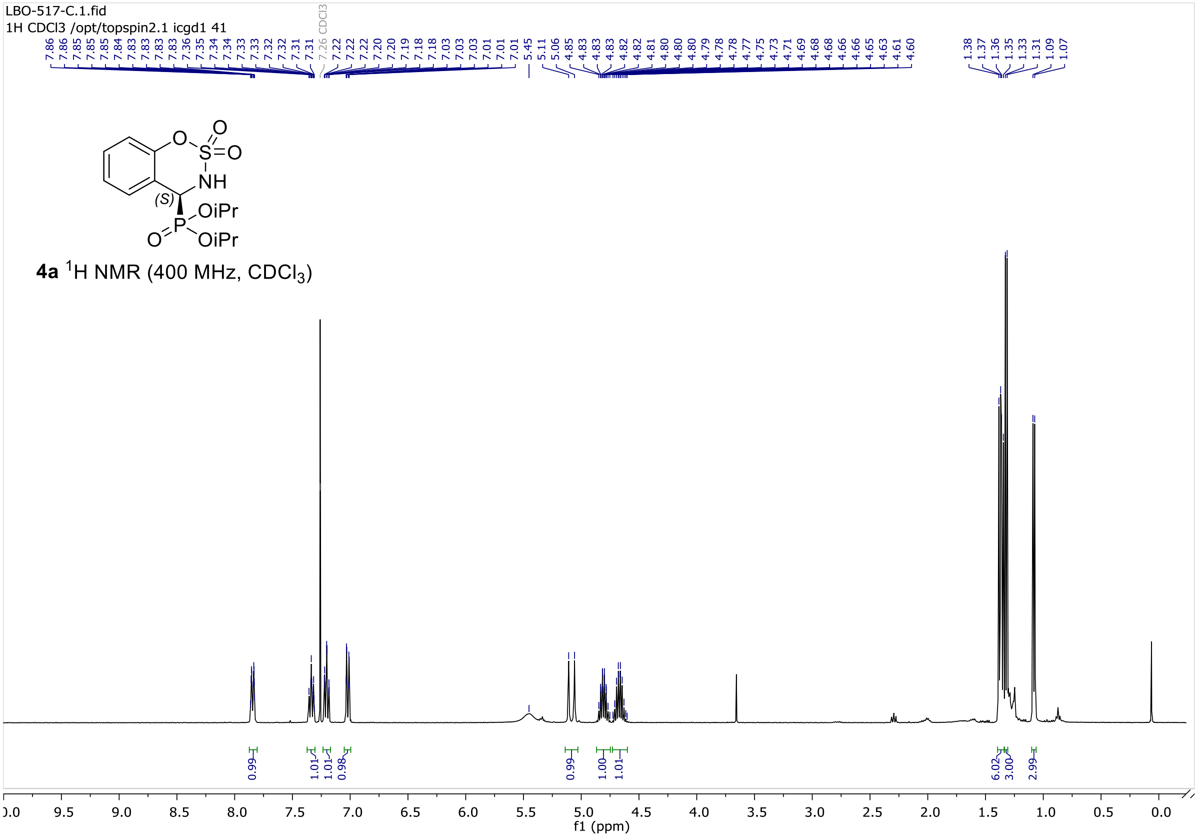
LBO-517-C.1.fid
1H CDCl3 /opt/topspin2.1 icgd1 41
7.86 7.86 7.85 7.85 7.84 7.83 7.83 7.83 7.83 7.36 7.35 7.34 7.34 7.33 7.33 7.32 7.32 7.31 7.31 7.26 CDCl3 7.22 7.22 7.22 7.22 7.20 7.20 7.19 7.18 7.18 7.03 7.03 7.03 7.01 7.01 7.01
5.45 5.11 5.06 4.85 4.83 4.83 4.83 4.82 4.81 4.80 4.80 4.79 4.78 4.78 4.77 4.75 4.73 4.71 4.69 4.68 4.68 4.66 4.66 4.65 4.63 4.61 4.60
1.38 1.37 1.36 1.35 1.33 1.31 1.09 1.07
4a ^{1}H NMR (400 MHz, $CDCl_3$)
0.99 1.01 1.01 0.98 0.99 1.00 1.01 6.02 3.00 2.99
f1 (ppm)

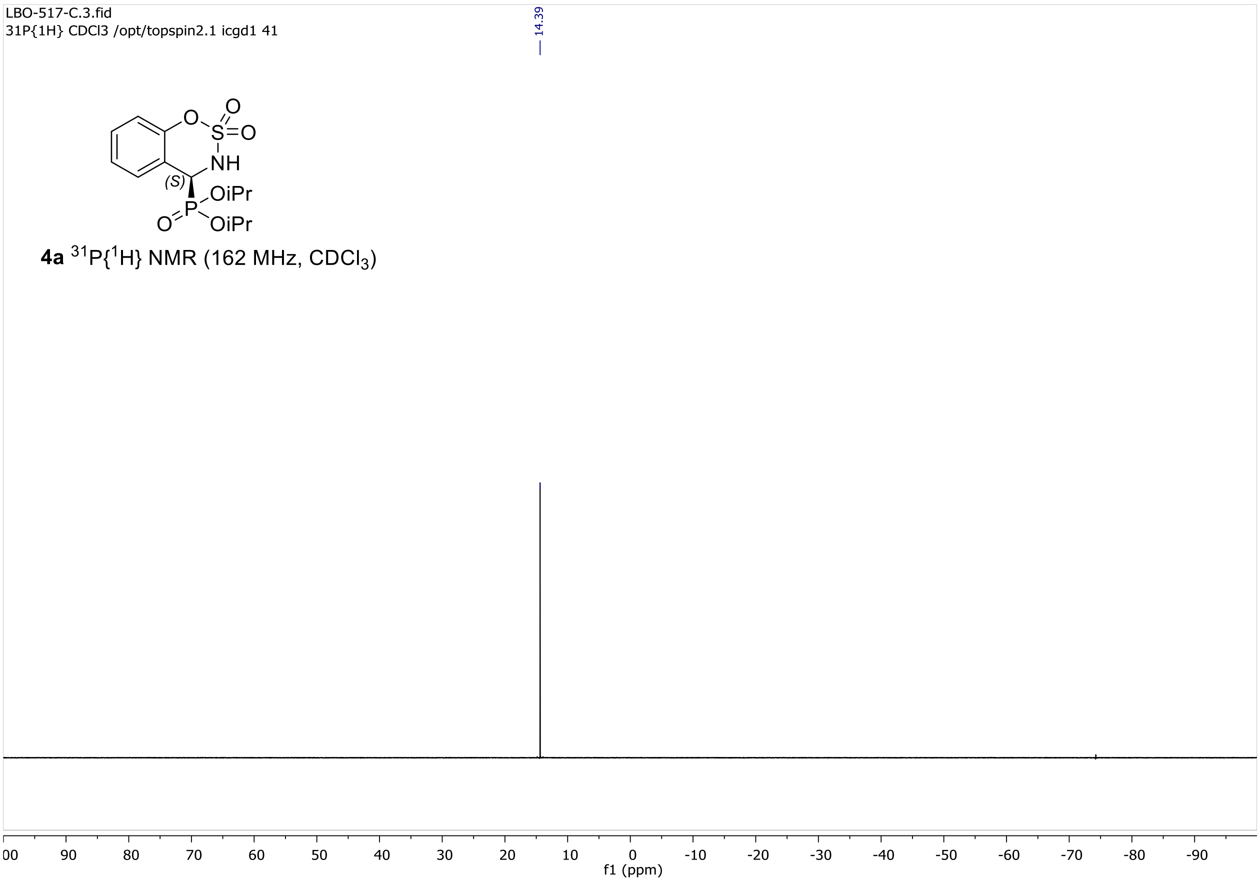
LBO-517-C.3.fid
31P{1H} CDCl3 /opt/topspin2.1 icgd1 41
14.39
4a $^{31}P\{^{1}H\}$ NMR (162 MHz, $CDCl_3$)
f1 (ppm)

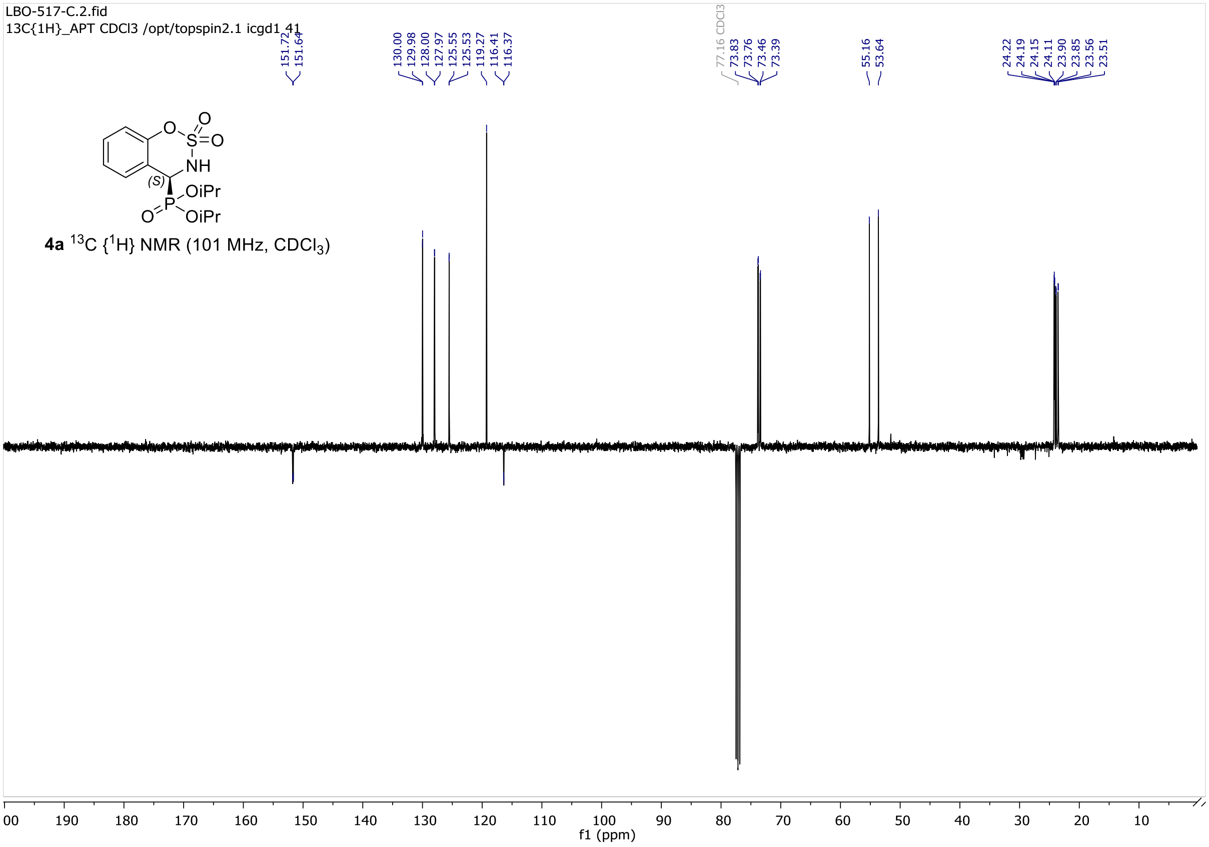
LBO-517-C.2.fid
13C{1H}_APT CDCl3 /opt/topspin2.1 icgd1 41
4a $^{13}C\{^1H\}$ NMR (101 MHz, $CDCl_3$)
f1 (ppm)

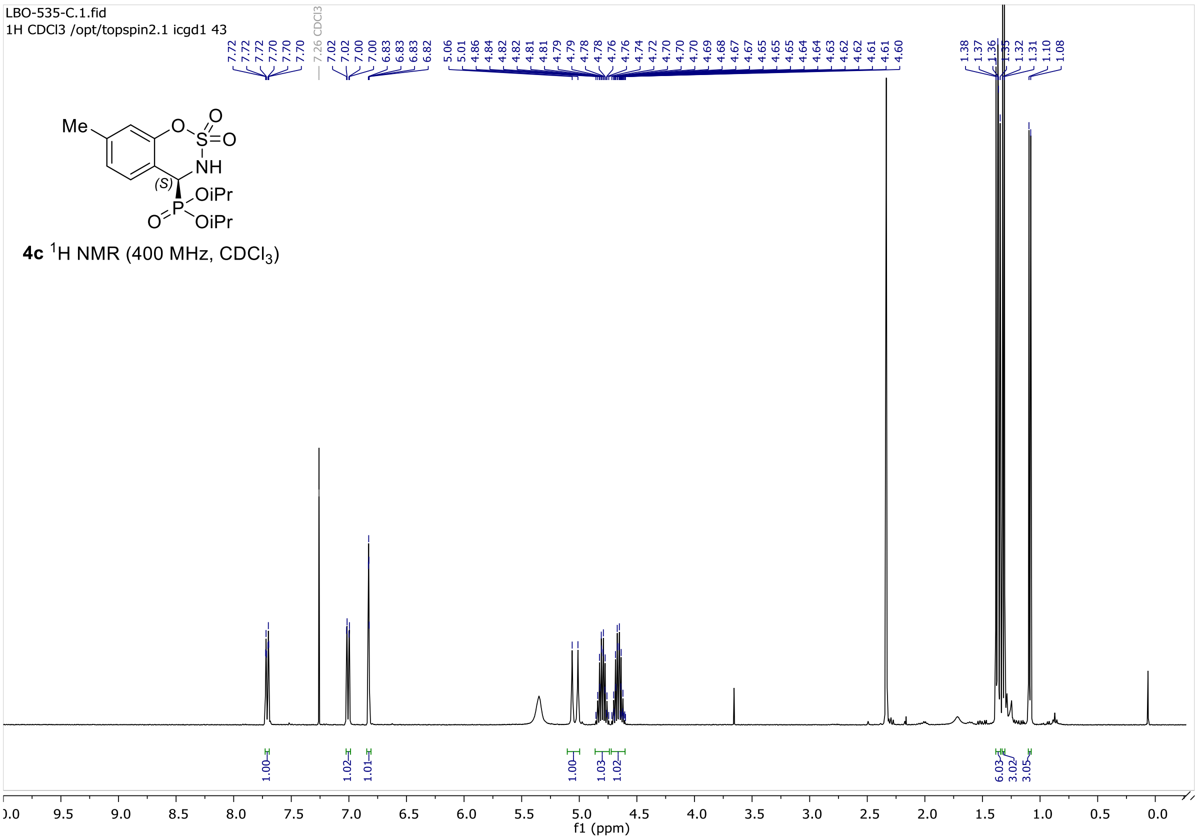
LBO-535-C.1.fid
1H CDCl3 /opt/topspin2.1 icgd1 43
4c ^1H NMR (400 MHz, $CDCl_3$)
f1 (ppm)

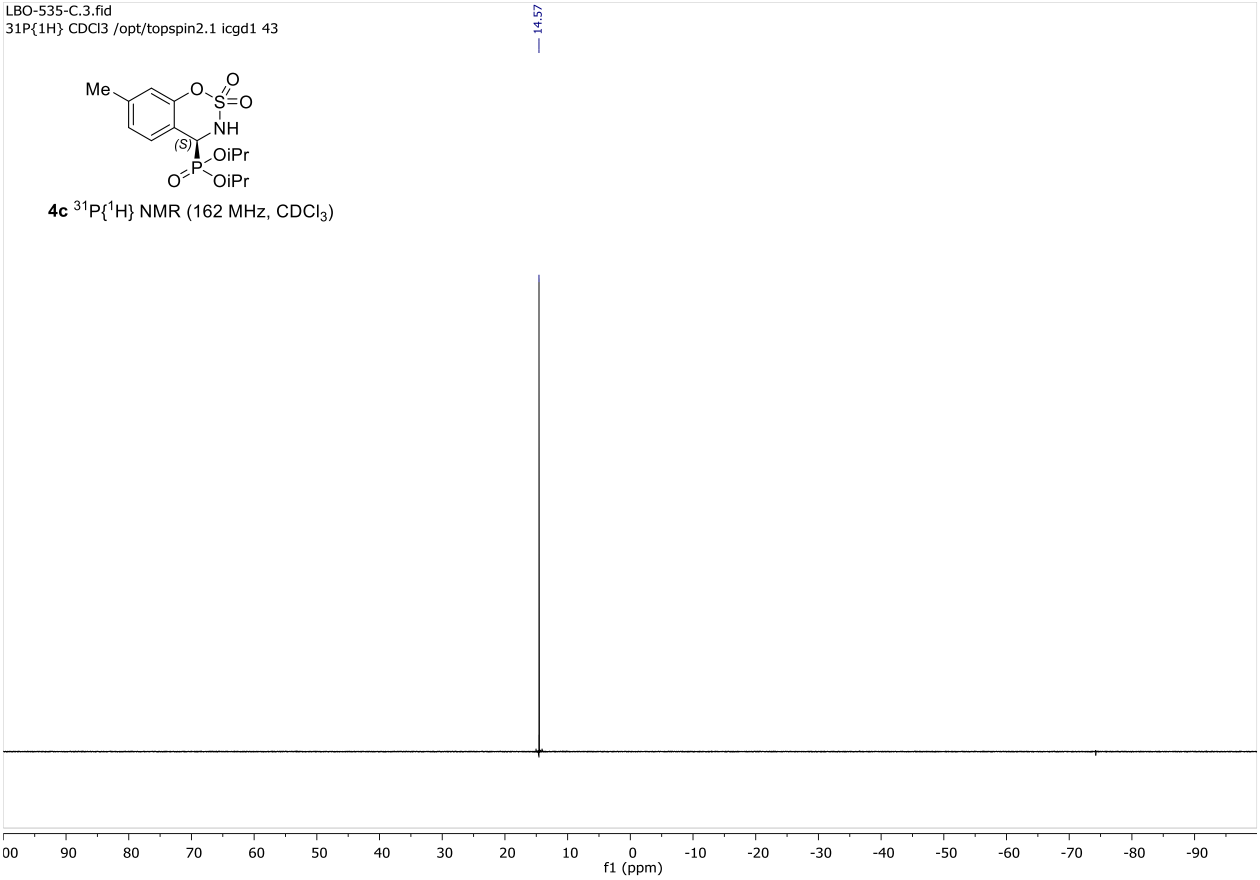

LBO-535-C.3.fid
31P{1H} CDCl3 /opt/topspin2.1 icgd1 43
14.57
Me
O
S
O
O
NH
(S)
OiPr
P
O
OiPr
4c 31P{1H} NMR (162 MHz, CDCl3)
f1 (ppm)

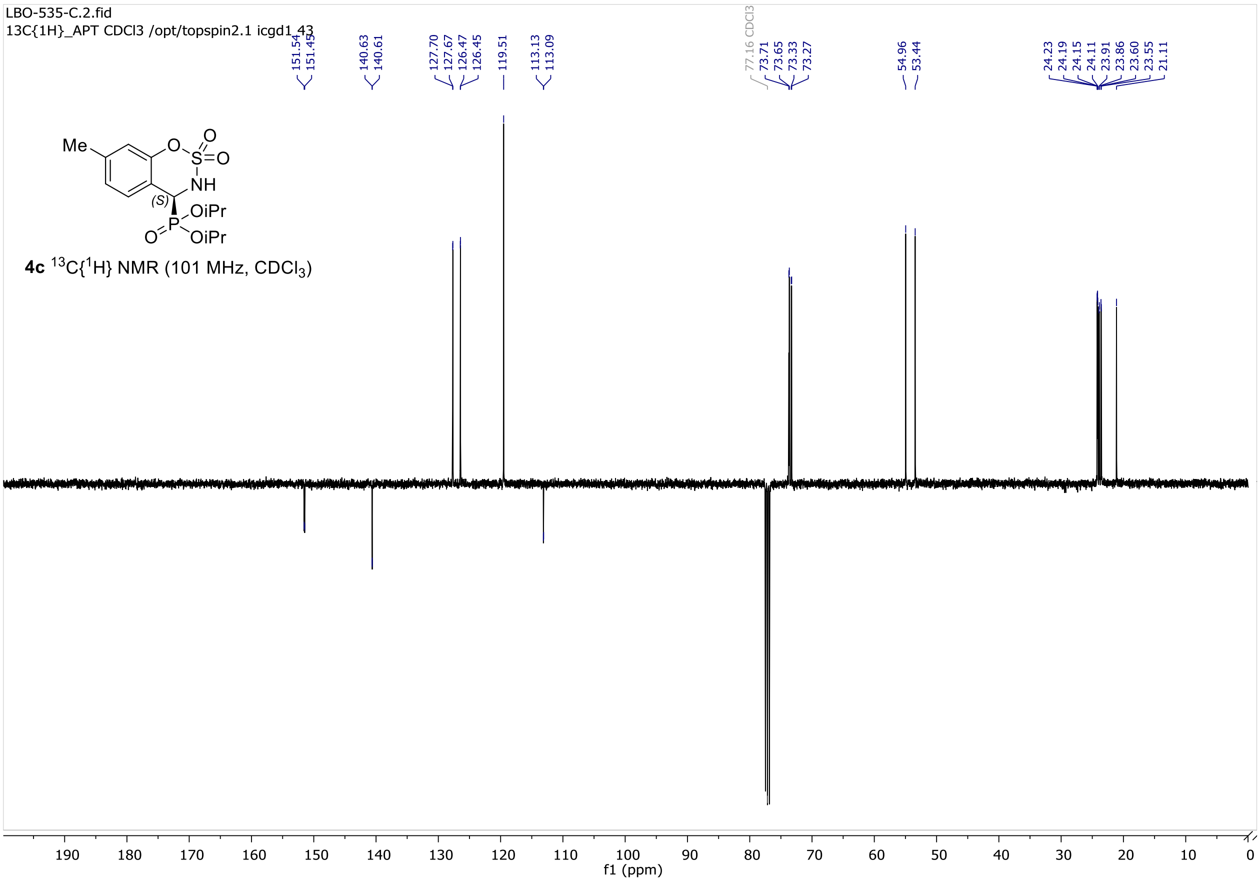

LBO-535-C.2.fid
13C{1H}_APT CDCl3 /opt/topspin2.1 icgd1 43
151.54
151.45
140.63
140.61
127.70
127.67
126.47
126.45
119.51
113.13
113.09
77.16 CDCl3
73.71
73.65
73.33
73.27
54.96
53.44
24.23
24.19
24.15
24.11
23.91
23.86
23.60
23.55
21.11
Me
O
S
O
O
NH
(S)
OiPr
P
O
OiPr
4c 13C{1H} NMR (101 MHz, CDCl3)
f1 (ppm)

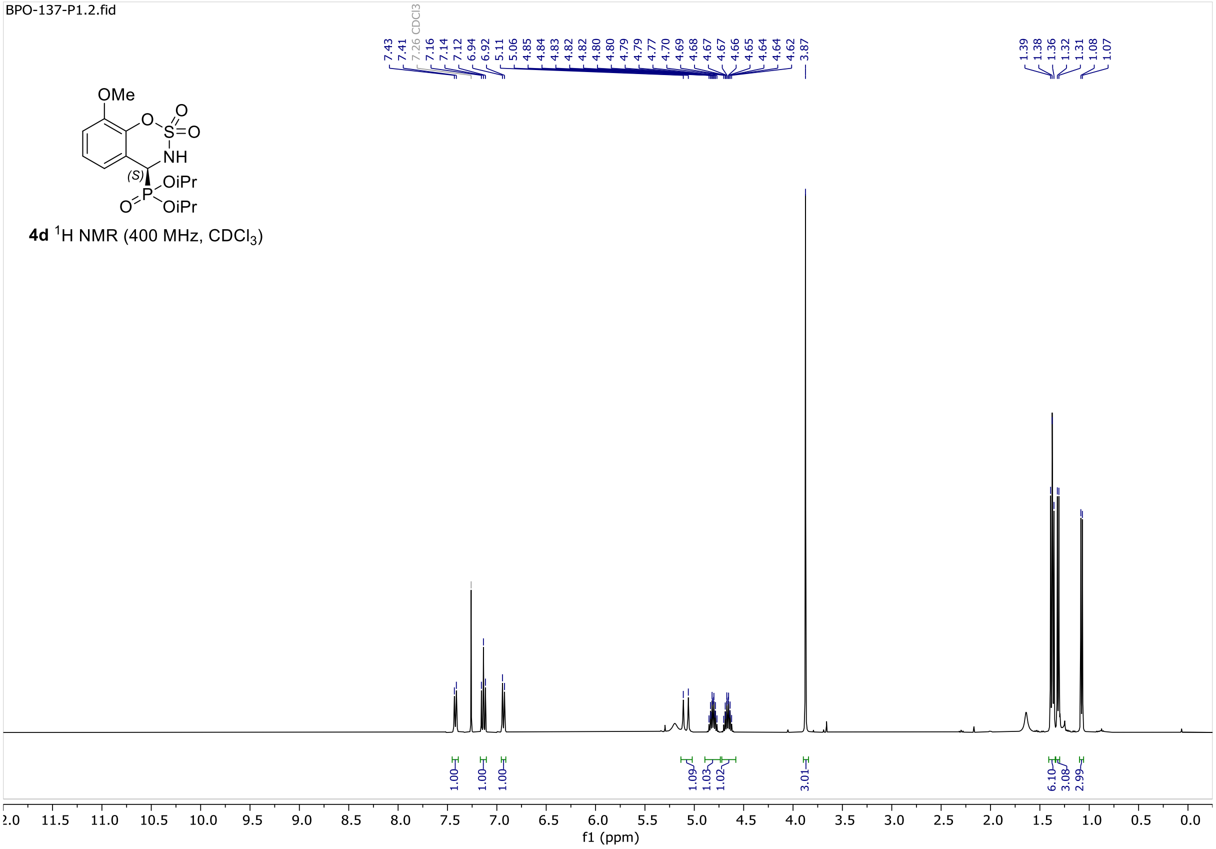
BPO-137-P1.2.fid
7.43
7.41
7.26 CDCl3
7.16
7.14
7.12
6.94
6.92
5.11
5.06
4.85
4.84
4.83
4.82
4.82
4.80
4.80
4.79
4.79
4.77
4.70
4.69
4.68
4.67
4.67
4.66
4.65
4.64
4.64
4.62
3.87
1.39
1.38
1.36
1.32
1.31
1.08
1.07
OMe
O
S
O
O
NH
(S)
OiPr
O
P
OiPr
4d ¹H NMR (400 MHz, CDCl3)
1.00
1.00
1.00
1.09
1.03
1.02
3.01
6.10
3.08
2.99
f1 (ppm)

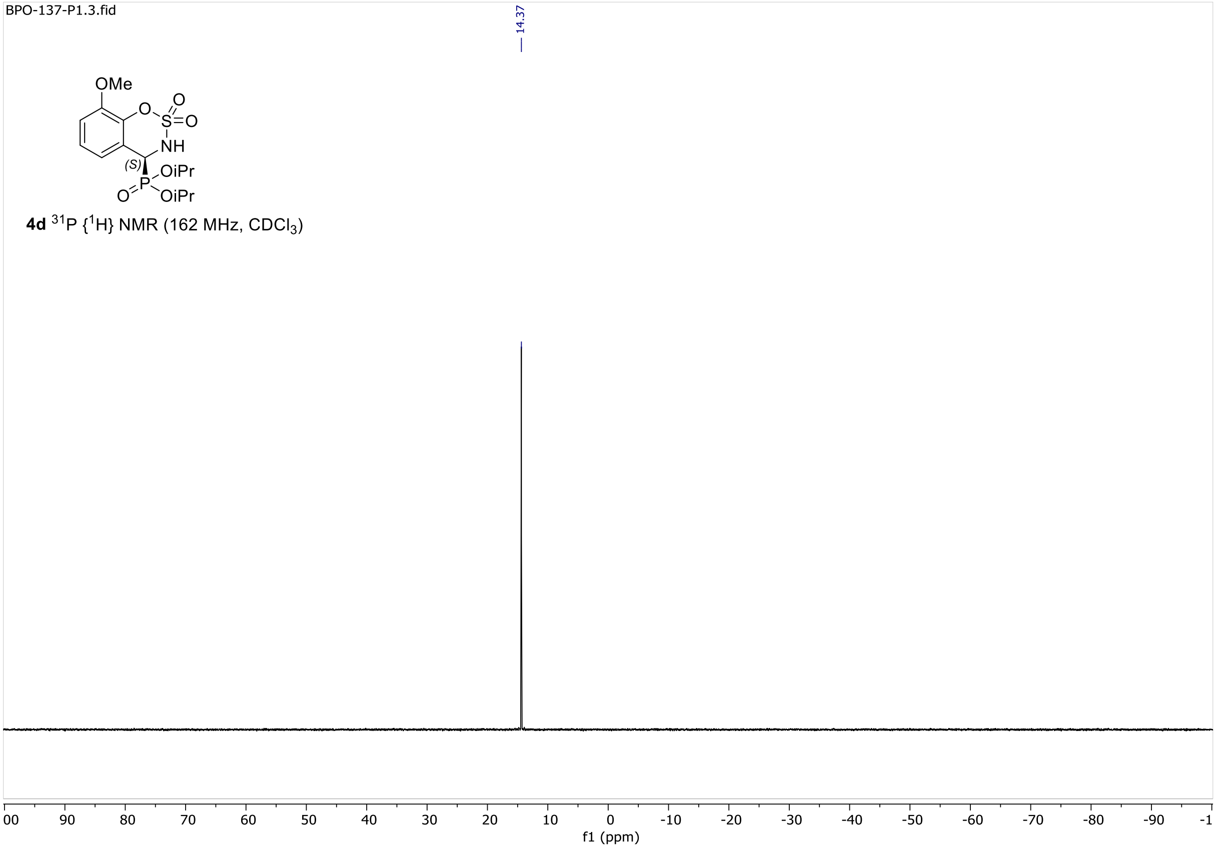
BPO-137-P1.3.fid
14.37
OMe
O
S
O
O
NH
(S)
OiPr
O
P
OiPr
4d ³¹P {¹H} NMR (162 MHz, CDCl3)
f1 (ppm)

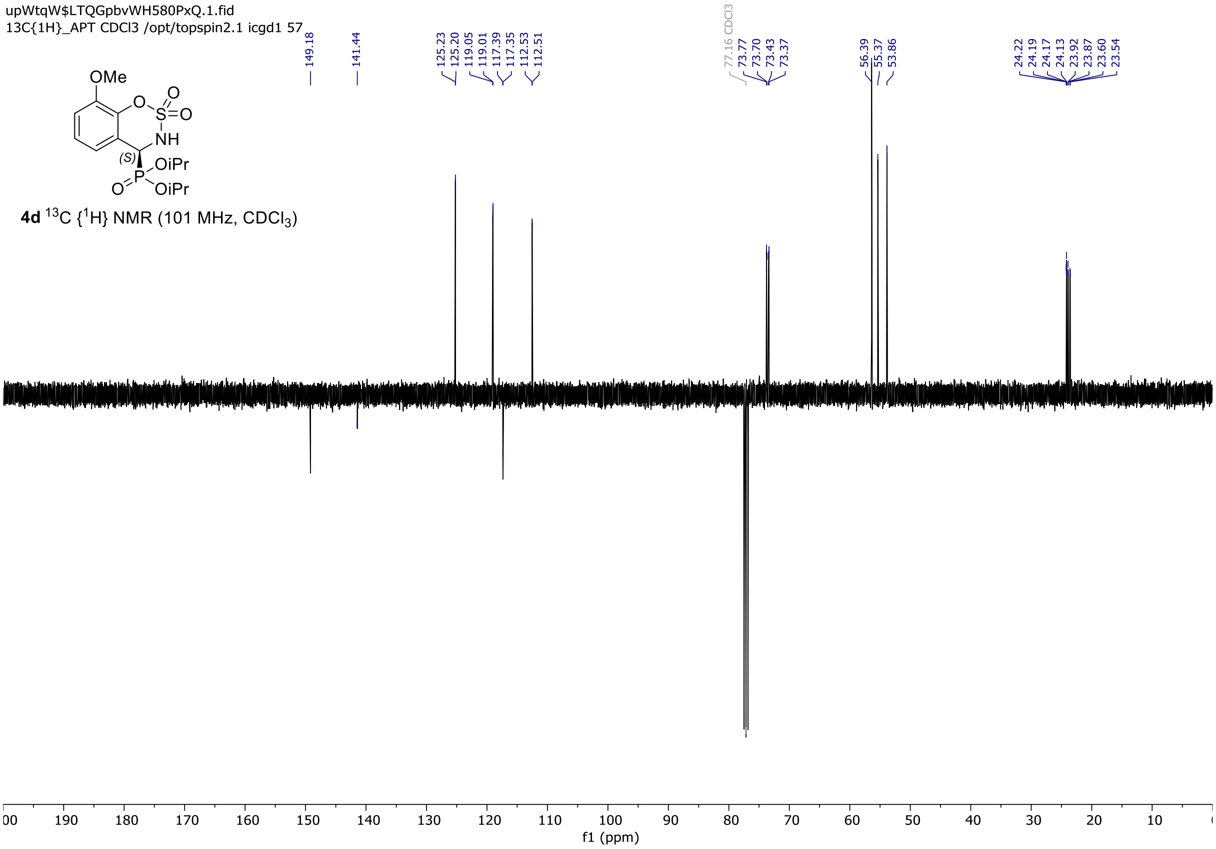

upWtqW$LTQGpbvWH580PxQ.1.fid
13C{1H}_APT CDCl3 /opt/topspin2.1 icgd1 57
OMe
NH
(S)
OiPr
OiPr
4d 13C {1H} NMR (101 MHz, CDCl3)
149.18
141.44
125.23
125.20
119.05
119.01
117.39
117.35
112.53
112.51
77.16 CDCl3
73.77
73.70
73.43
73.37
56.39
55.37
53.86
24.22
24.19
24.17
24.13
23.92
23.87
23.60
23.54
f1 (ppm)

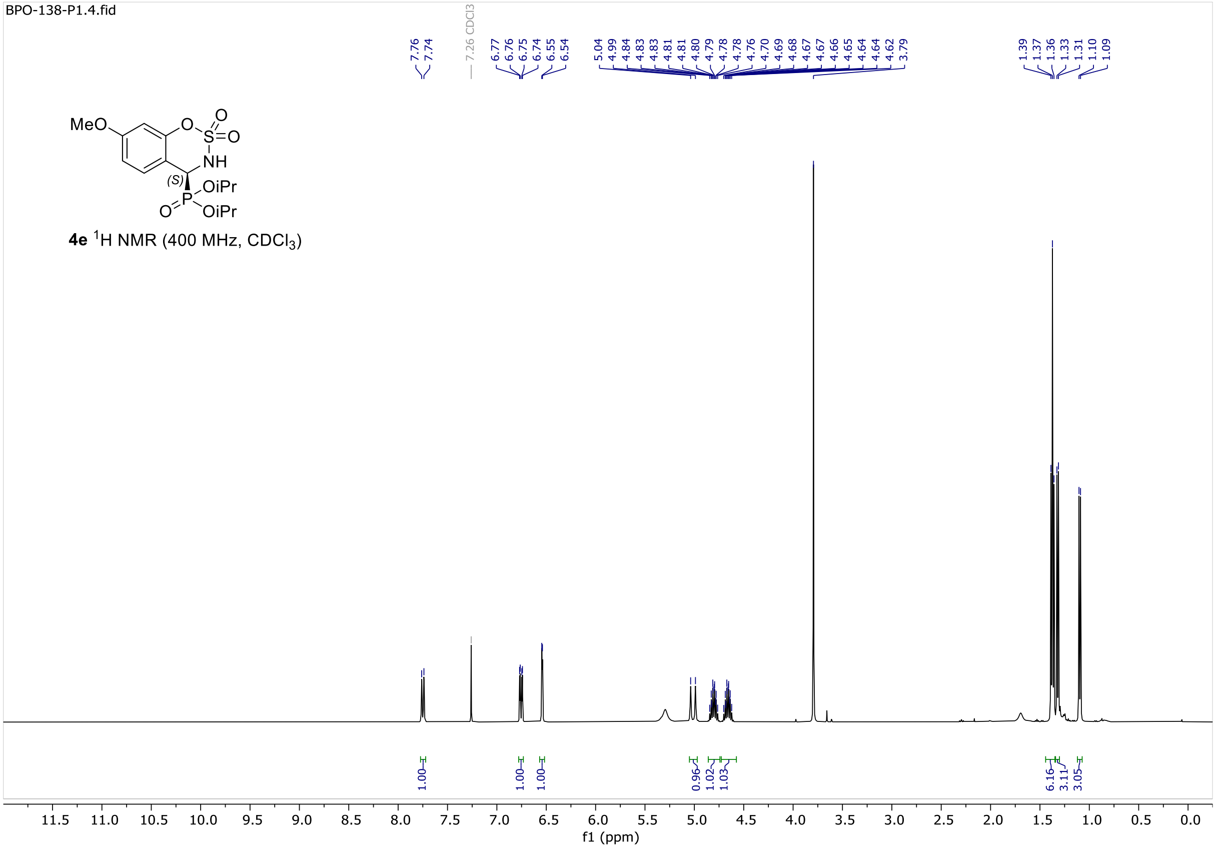

BPO-138-P1.4.fid
MeO
NH
(S)
OiPr
OiPr
4e 1H NMR (400 MHz, CDCl3)
7.76
7.74
7.26 CDCl3
6.77
6.76
6.75
6.74
6.55
6.54
5.04
4.99
4.84
4.83
4.83
4.81
4.80
4.79
4.78
4.76
4.70
4.69
4.68
4.67
4.67
4.66
4.65
4.64
4.64
4.62
3.79
1.39
1.37
1.36
1.33
1.31
1.10
1.09
1.00
1.00
1.00
0.96
1.02
1.03
6.16
3.11
3.05
f1 (ppm)

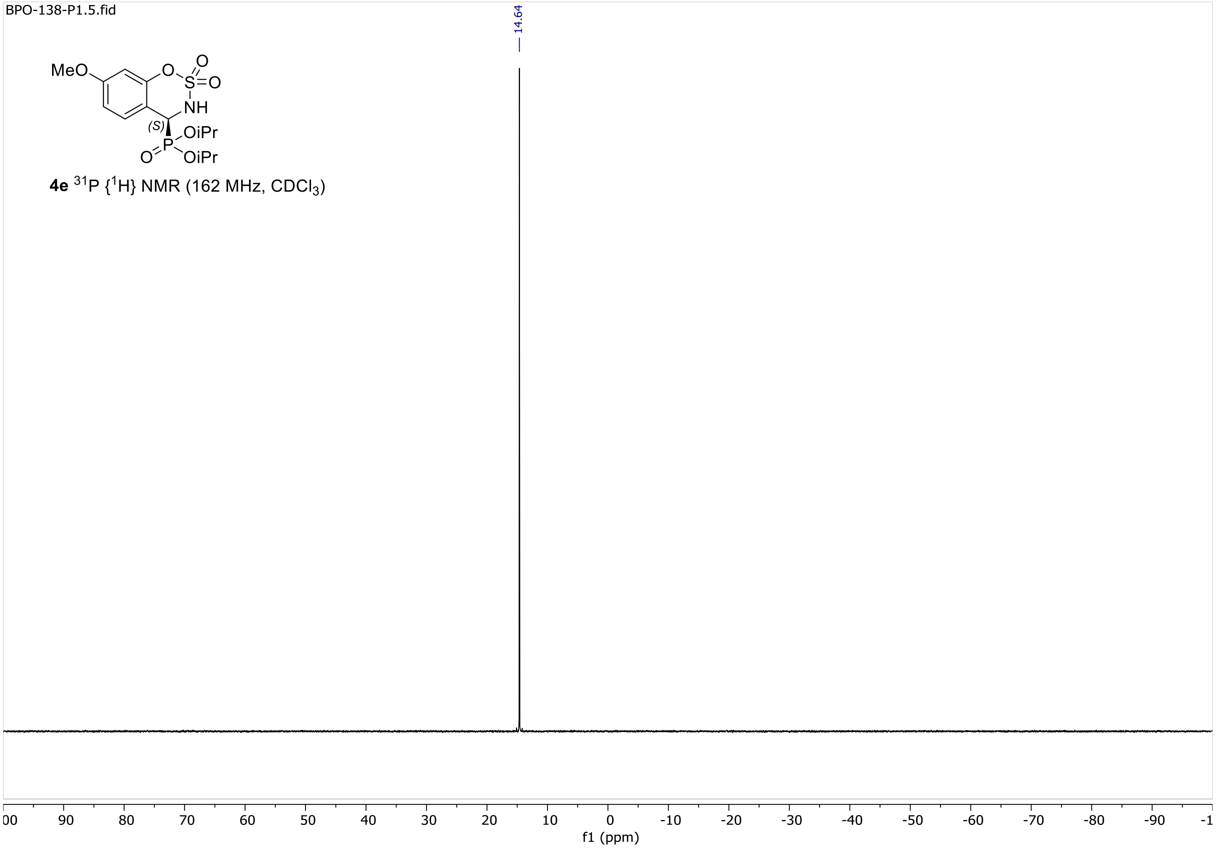

BPO-138-P1.5.fid
14.64
MeO
O
S
O
O
NH
(S)
OiPr
P
O
OiPr
4e 31P {1H} NMR (162 MHz, CDCl3)
f1 (ppm)

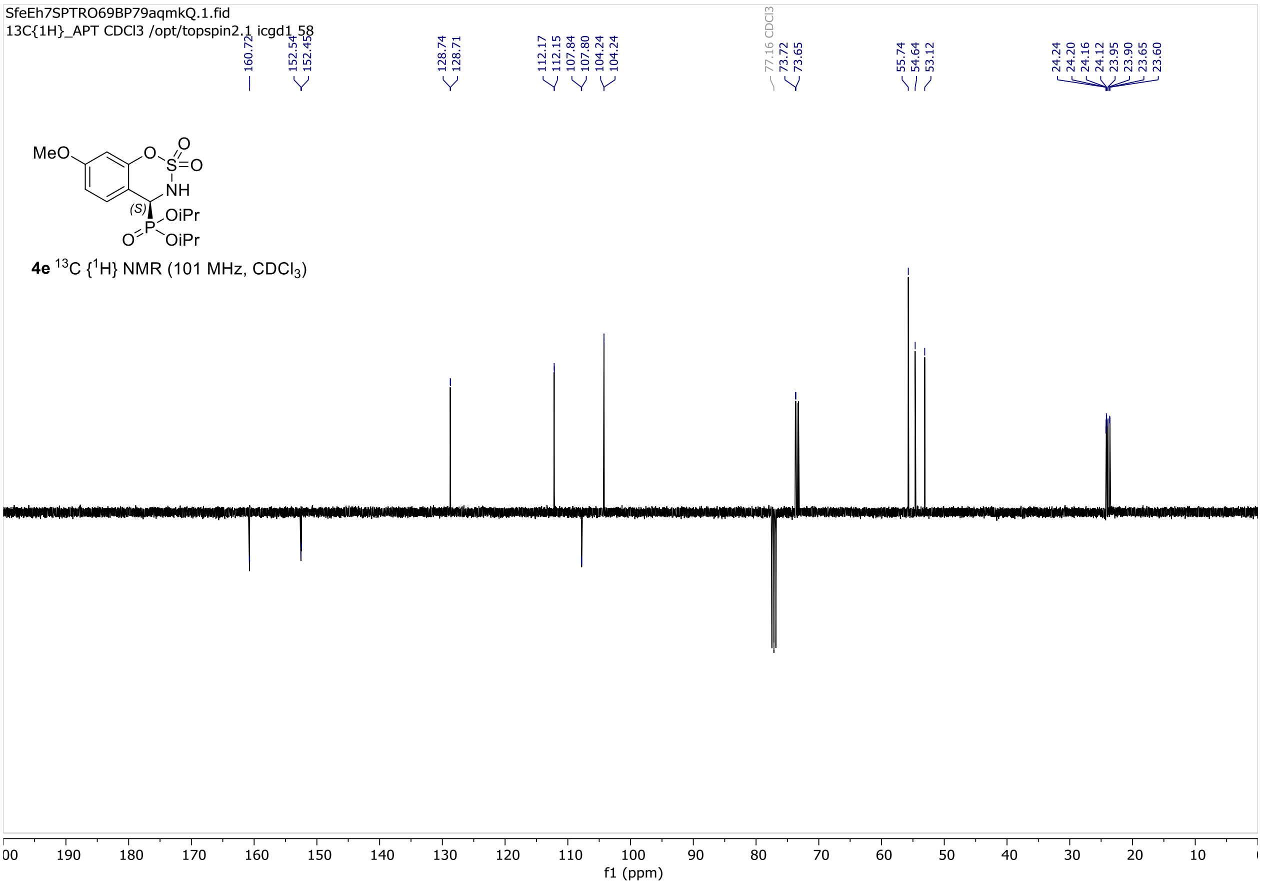

SfeEh7SPTRO69BP79aqmkQ.1.fid
13C{1H}_APT CDCl3 /opt/topspin2.1 icgd1 58
160.72
152.54
152.45
128.74
128.71
112.17
112.15
107.84
107.80
104.24
104.24
77.16 CDCl3
73.72
73.65
55.74
54.64
53.12
24.24
24.20
24.16
24.12
23.95
23.90
23.65
23.60
MeO
NH
(S)
OiPr
OiPr
4e 13C {1H} NMR (101 MHz, CDCl3)
f1 (ppm)

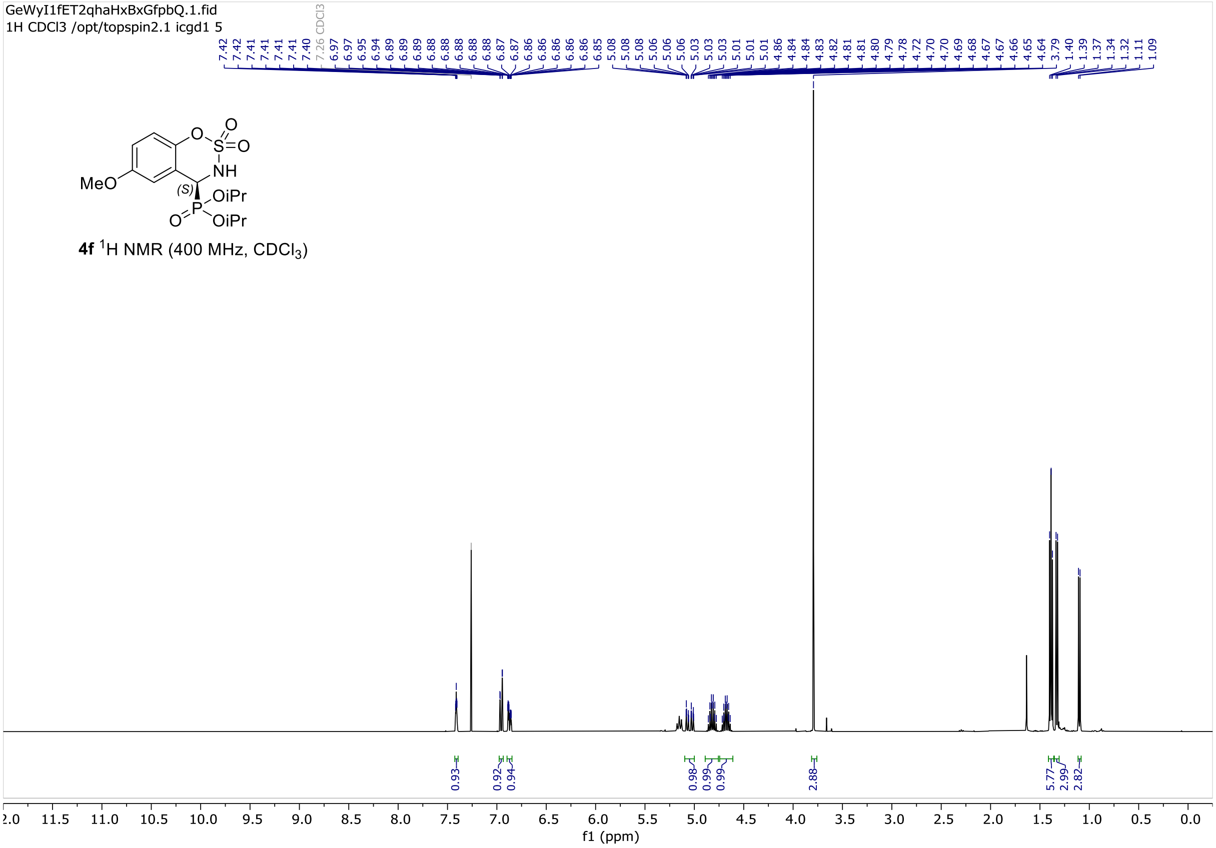

4f ^{1}H NMR (400 MHz, $CDCl_3$)

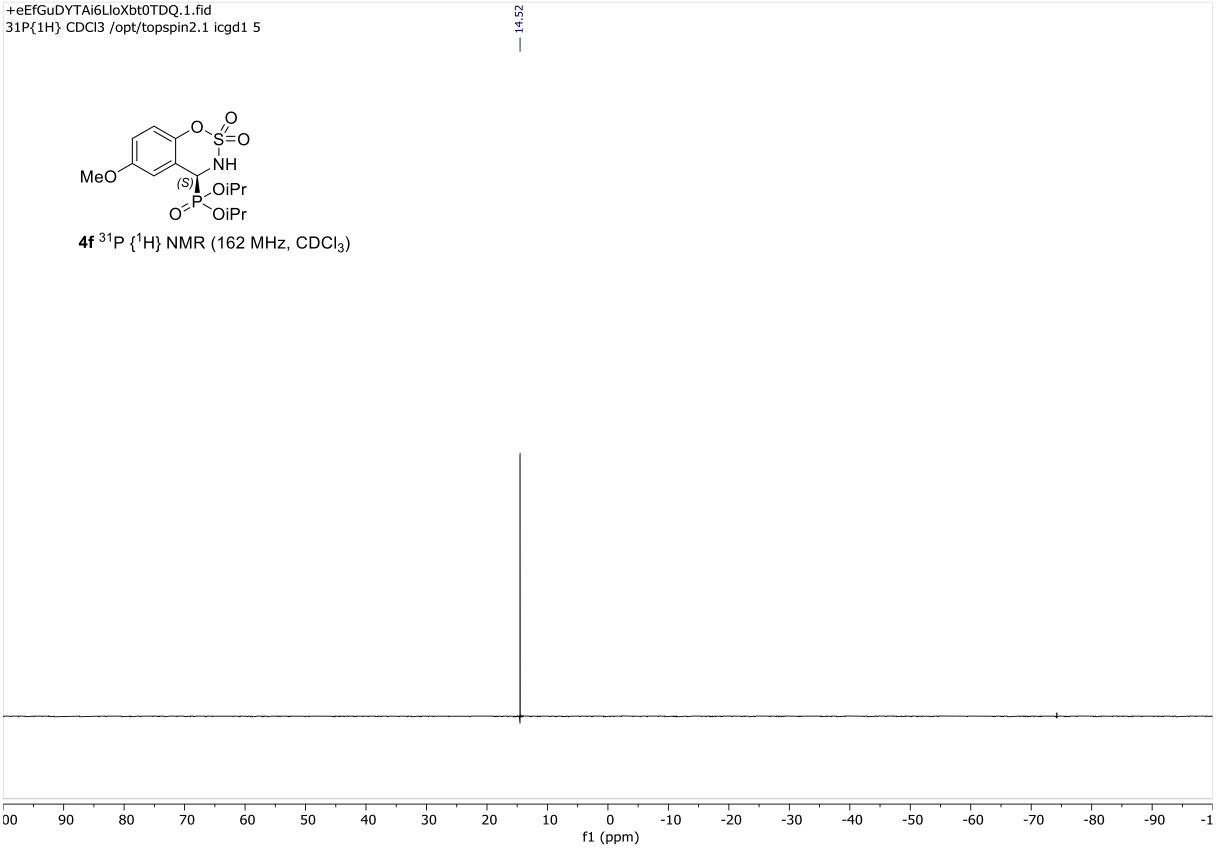

4f $^{31}P\{^{1}H\}$ NMR (162 MHz, $CDCl_3$)

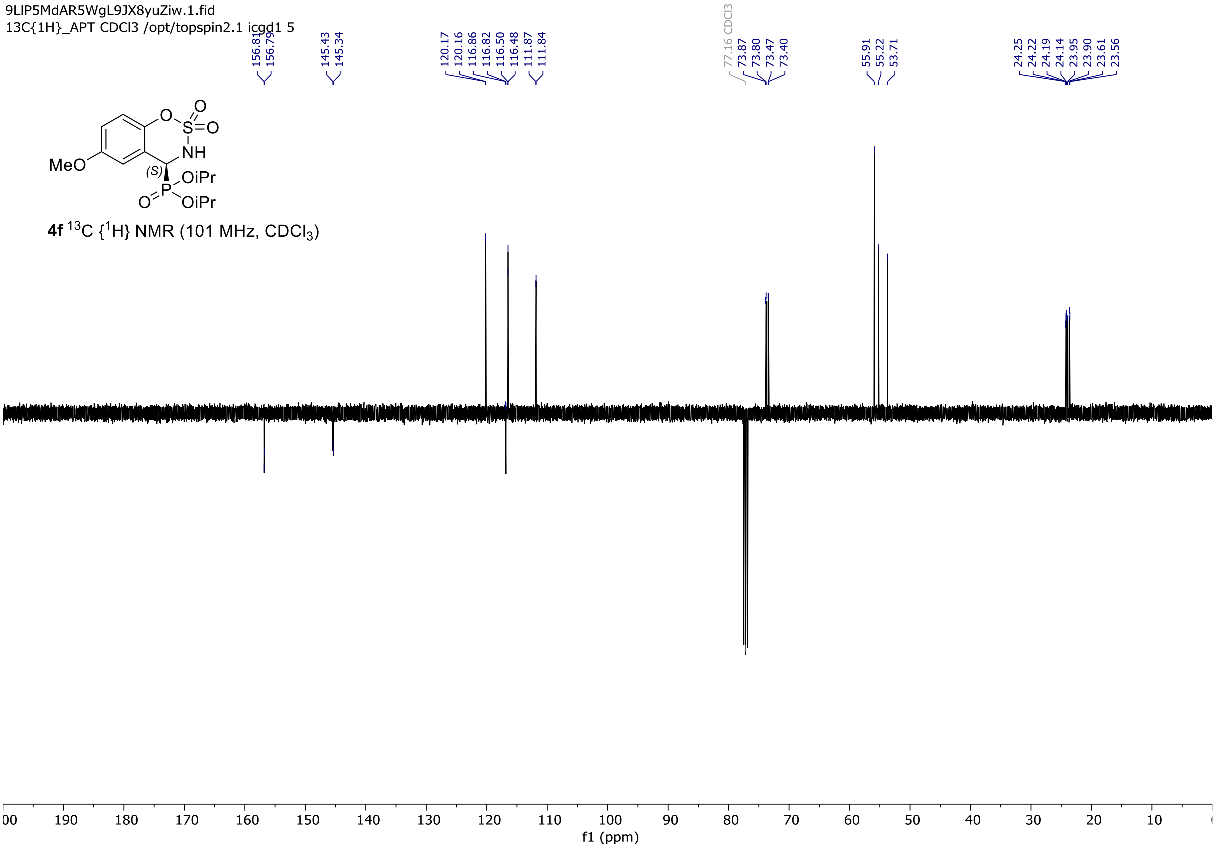
9LlP5MdAR5WgL9JX8yuZiw.1.fid
13C{1H}_APT CDCl3 /opt/topspin2.1 icgd1 5
156.81
156.79
145.43
145.34
120.17
120.16
116.86
116.82
116.50
116.48
111.87
111.84
77.16 CDCl3
73.87
73.80
73.47
73.40
55.91
55.22
53.71
24.25
24.22
24.19
24.14
23.95
23.90
23.61
23.56
MeO
(S)
OiPr
OiPr
NH
4f 13C {1H} NMR (101 MHz, CDCl3)
f1 (ppm)

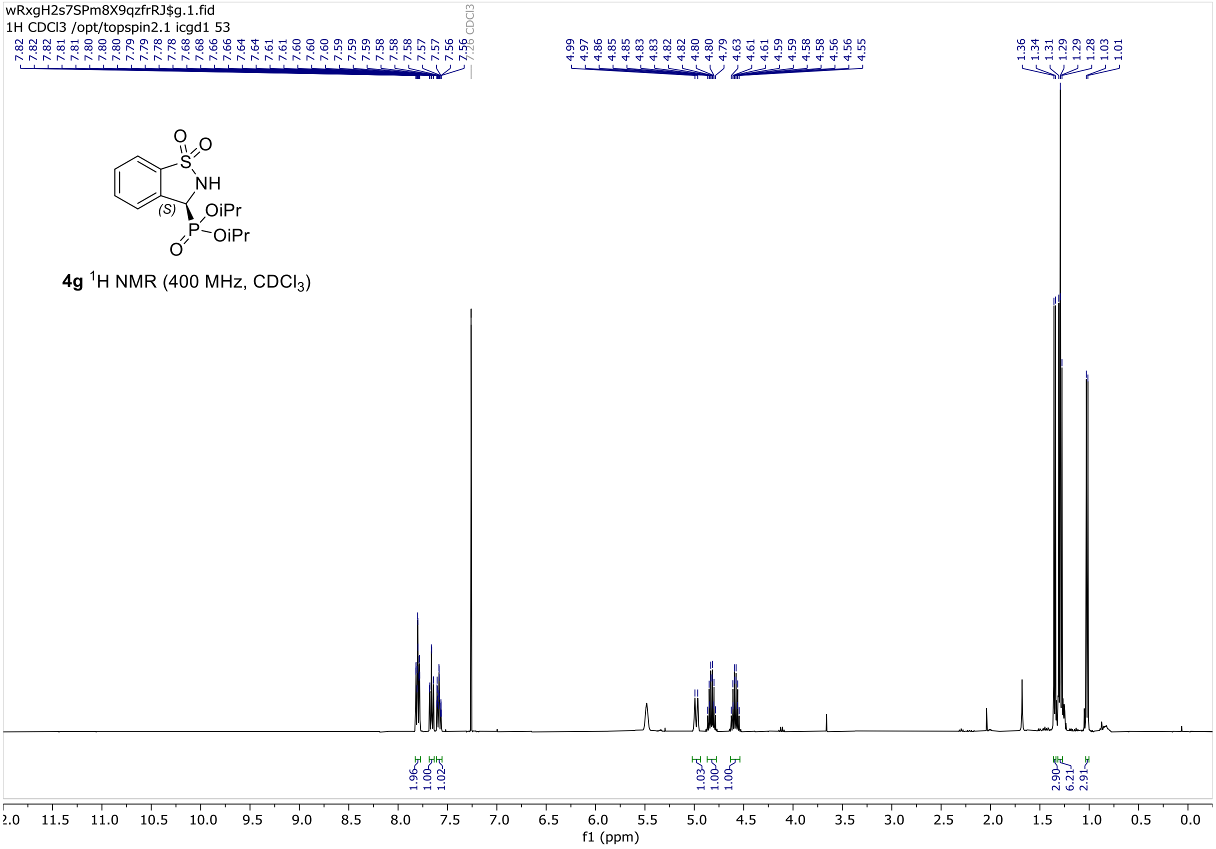
wRxgH2s7SPm8X9qzfrRJ$g.1.fid
1H CDCl3 /opt/topspin2.1 icgd1 53
7.82
7.82
7.81
7.80
7.80
7.80
7.79
7.78
7.68
7.66
7.66
7.64
7.64
7.61
7.61
7.60
7.60
7.60
7.59
7.59
7.59
7.58
7.58
7.58
7.57
7.56
7.56
7.26 CDCl3
4.99
4.97
4.86
4.85
4.85
4.83
4.83
4.82
4.82
4.80
4.80
4.79
4.63
4.61
4.61
4.59
4.59
4.58
4.58
4.56
4.56
4.55
1.36
1.34
1.31
1.29
1.29
1.28
1.03
1.01
NH
(S)
OiPr
OiPr
4g 1H NMR (400 MHz, CDCl3)
1.96
1.00
1.02
1.03
1.00
1.00
2.90
6.21
2.91
f1 (ppm)

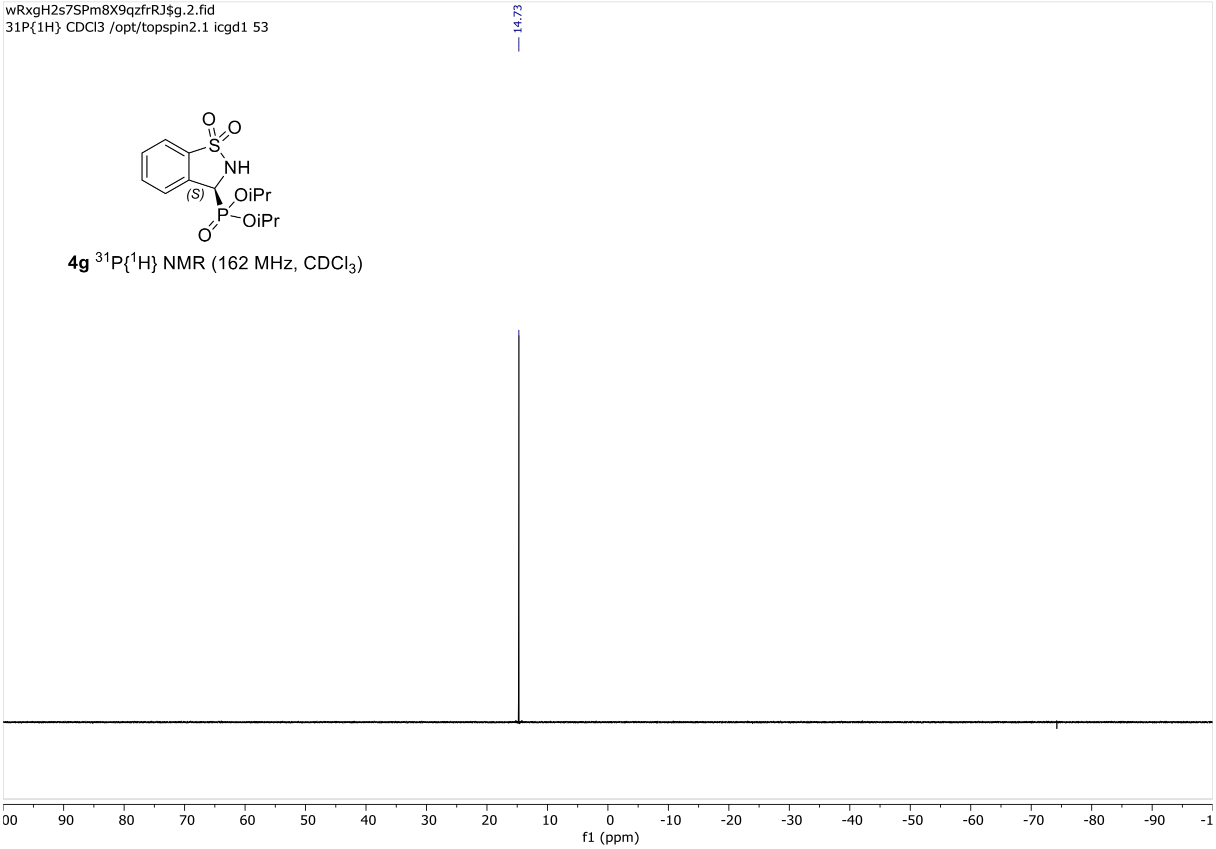
wRxgH2s7SPm8X9qzfrRJ$g.2.fid
31P{1H} CDCl3 /opt/topspin2.1 icgd1 53
14.73
4g ³¹P{¹H} NMR (162 MHz, CDCl3)
f1 (ppm)

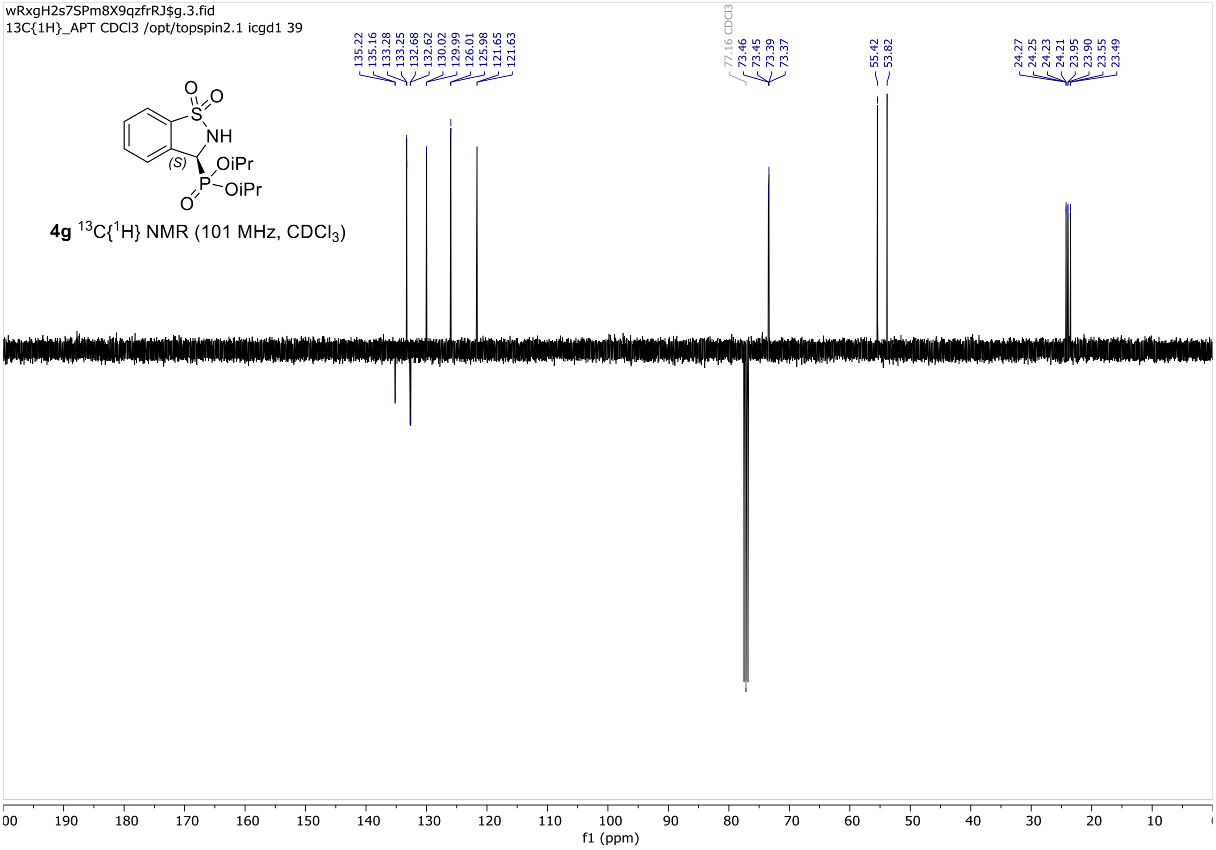
wRxgH2s7SPm8X9qzfrRJ$g.3.fid
13C{1H}_APT CDCl3 /opt/topspin2.1 icgd1 39
135.22
135.16
133.28
133.25
132.68
132.62
130.02
129.99
126.01
125.98
121.65
121.63
77.16 CDCl3
73.46
73.45
73.39
73.37
55.42
53.82
24.27
24.25
24.23
24.21
23.95
23.90
23.55
23.49
4g ¹³C{¹H} NMR (101 MHz, CDCl3)
f1 (ppm)

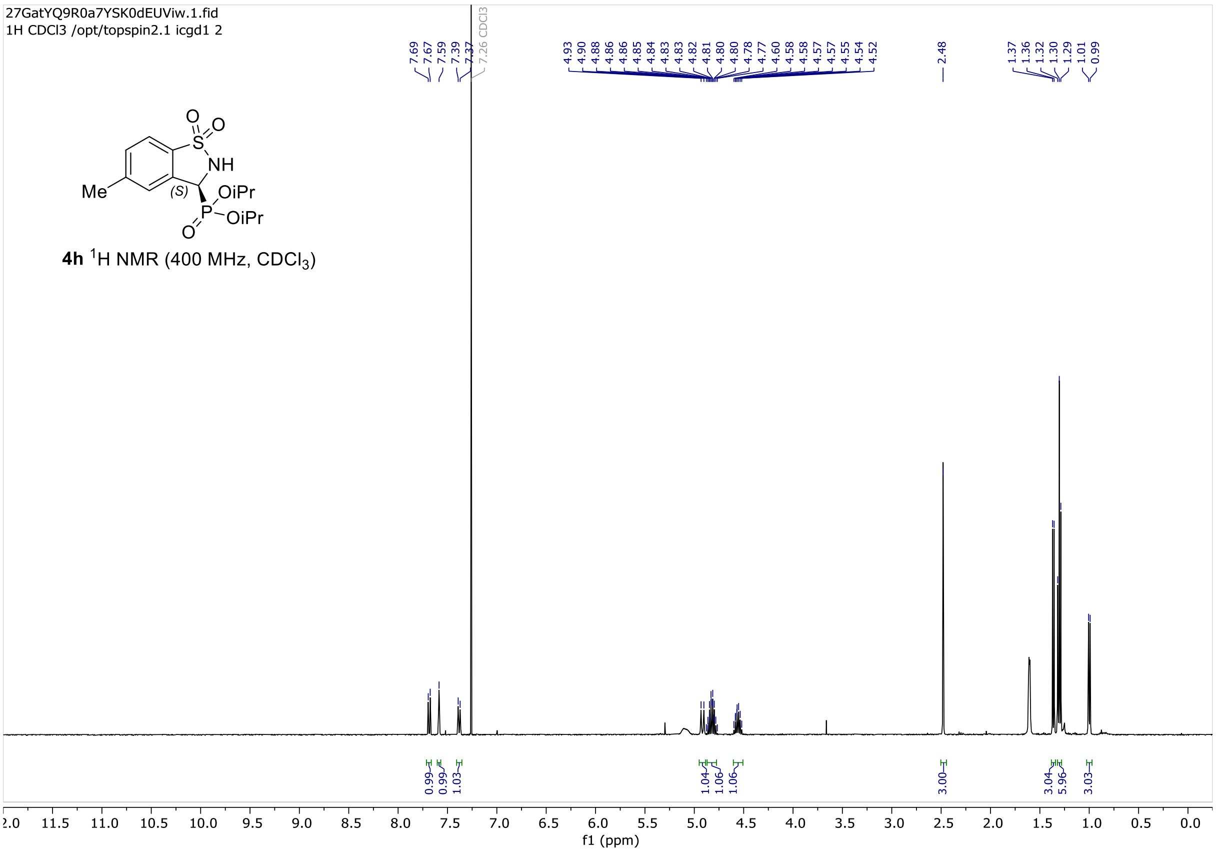
27GatYQ9R0a7YSK0dEUViw.1.fid
1H CDCl3 /opt/topspin2.1 icgd1 2
4h 1H NMR (400 MHz, CDCl3)
7.69 7.67 7.59 7.39 7.37 7.26 CDCl3
4.93 4.90 4.88 4.86 4.86 4.85 4.84 4.83 4.83 4.82 4.81 4.80 4.80 4.78 4.77 4.60 4.58 4.58 4.57 4.57 4.55 4.54 4.52
2.48
1.37 1.36 1.32 1.30 1.29 1.01 0.99
0.99 0.99 1.03 1.04 1.06 1.06 3.00 3.04 5.96 3.03
f1 (ppm)

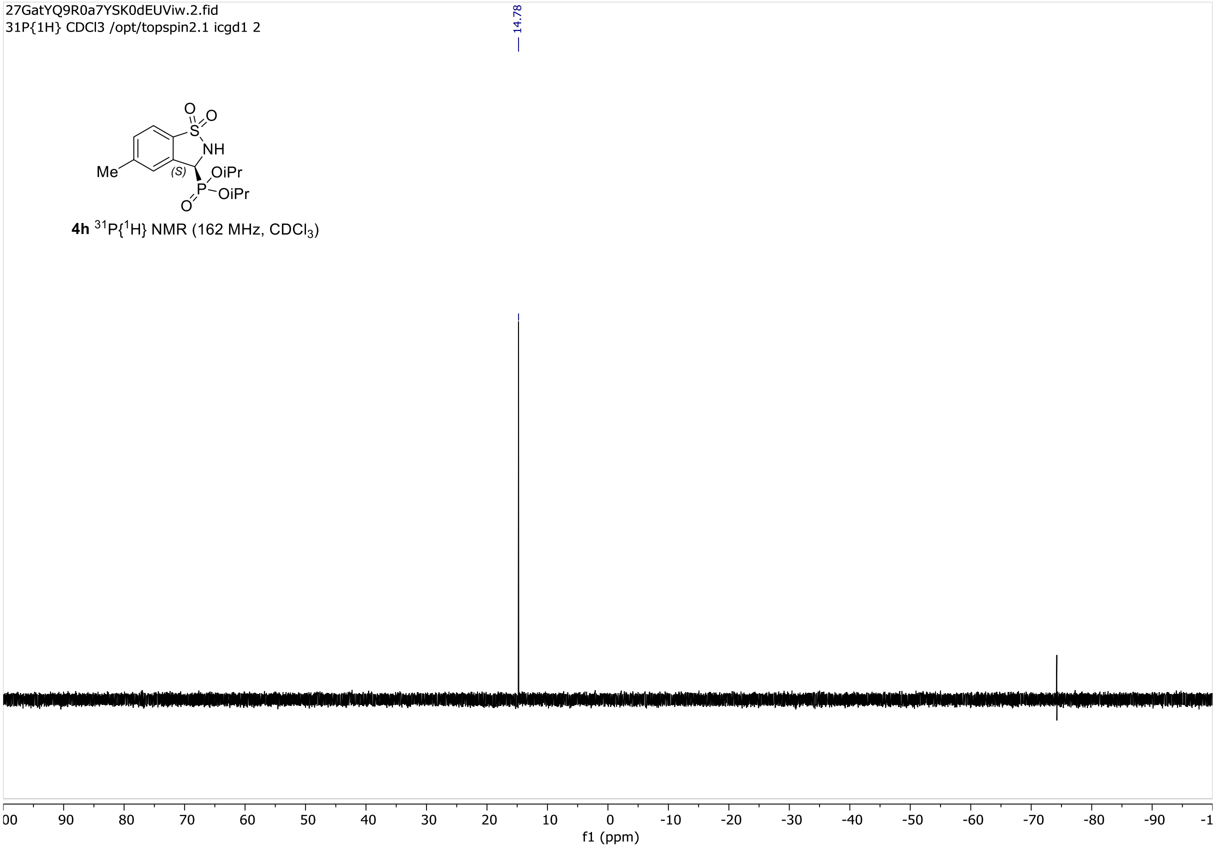
27GatYQ9R0a7YSK0dEUViw.2.fid
31P{1H} CDCl3 /opt/topspin2.1 icgd1 2
4h 31P{1H} NMR (162 MHz, CDCl3)
14.78
f1 (ppm)

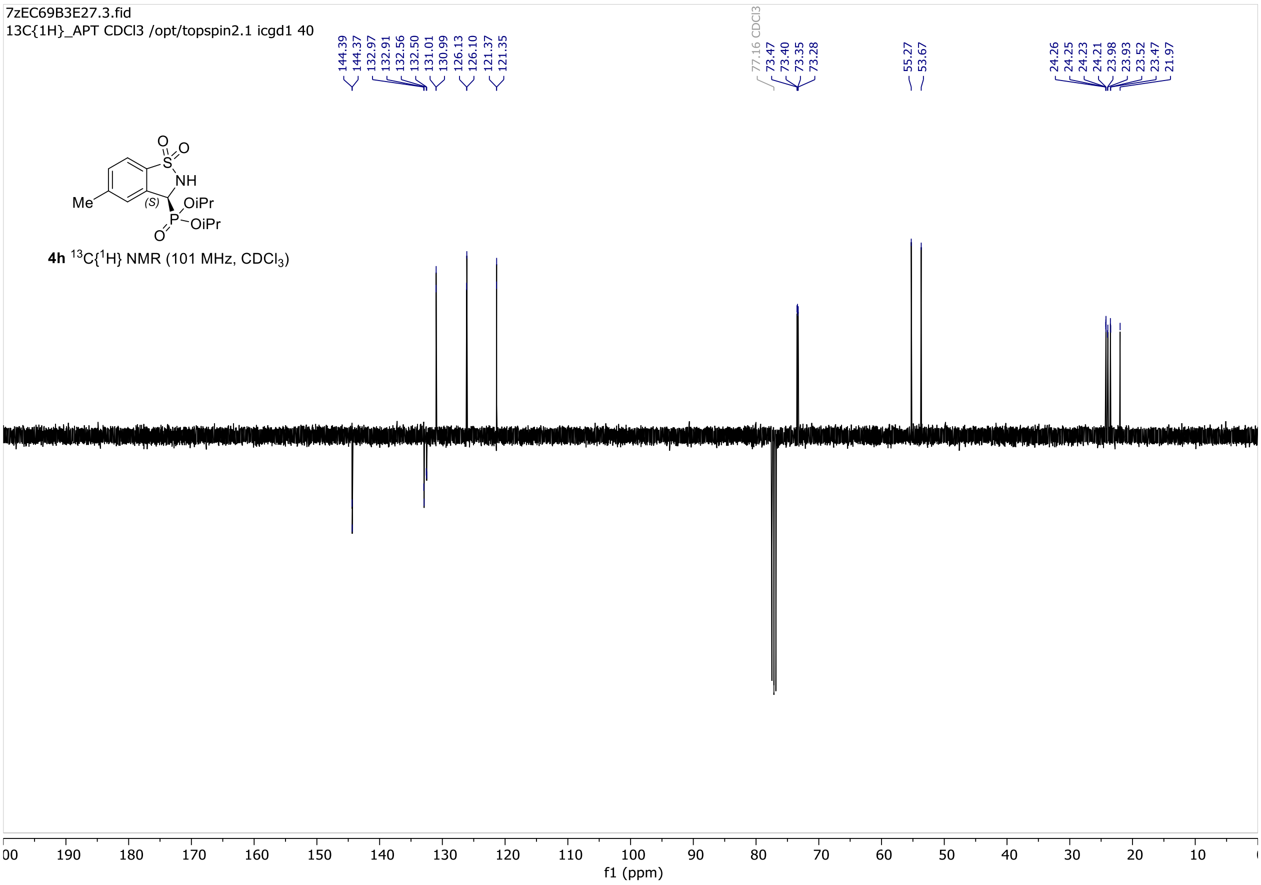
7zEC69B3E27.3.fid
13C{1H}_APT CDCl3 /opt/topspin2.1 icgd1 40
144.39
144.37
132.97
132.91
132.56
132.50
131.01
130.99
126.13
126.10
121.37
121.35
77.16 CDCl3
73.47
73.40
73.35
73.28
55.27
53.67
24.26
24.25
24.23
24.21
23.98
23.93
23.52
23.47
21.97
O
O
S
NH
Me
(S)
OiPr
P
OiPr
O
4h $^{13}C\{^{1}H\}$ NMR (101 MHz, $CDCl_3$)
f1 (ppm)

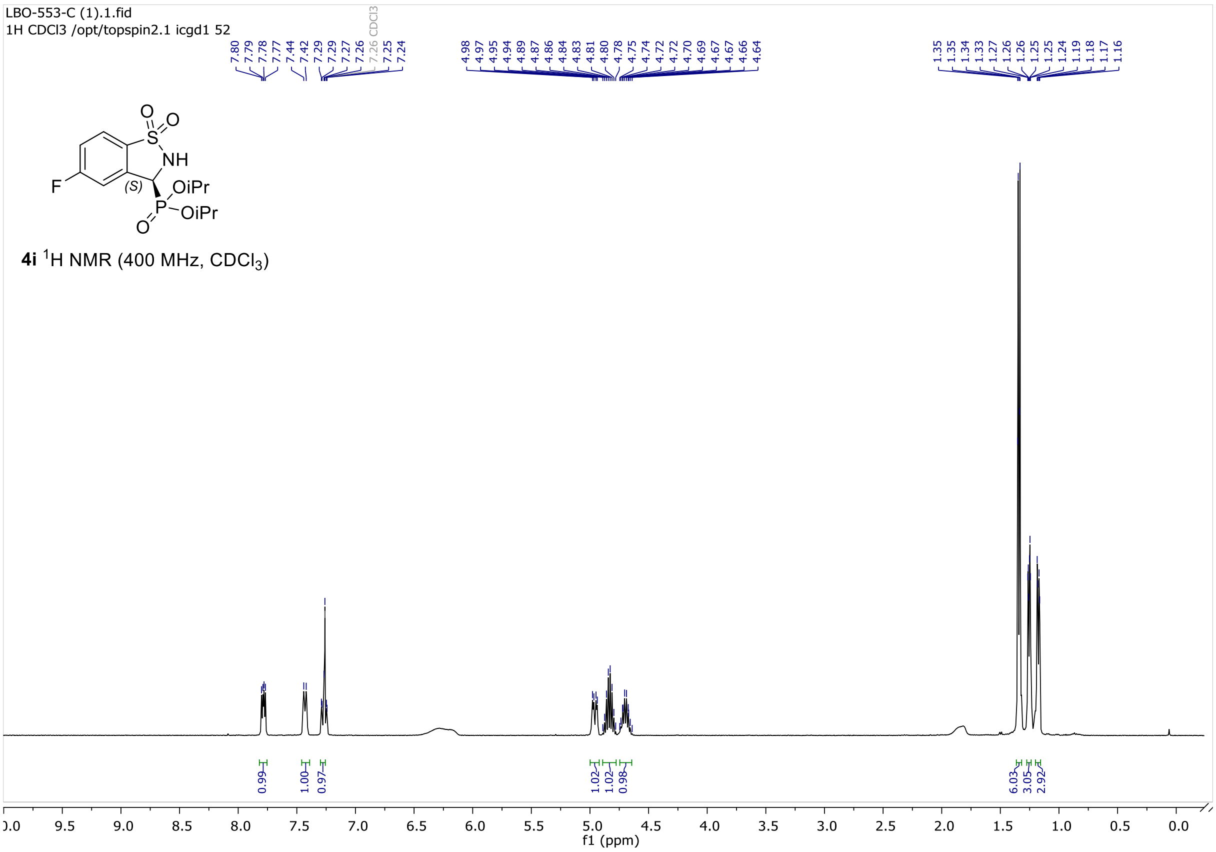
LBO-553-C (1).1.fid
1H CDCl3 /opt/topspin2.1 icgd1 52
7.80
7.79
7.78
7.77
7.44
7.42
7.29
7.29
7.27
7.26 CDCl3
7.25
7.24
4.98
4.97
4.95
4.94
4.89
4.87
4.86
4.84
4.83
4.81
4.80
4.78
4.75
4.74
4.72
4.70
4.69
4.67
4.66
4.64
1.35
1.35
1.34
1.33
1.27
1.26
1.26
1.25
1.25
1.24
1.19
1.18
1.17
1.16
O
O
S
NH
F
(S)
OiPr
P
OiPr
O
4i ^{1}H NMR (400 MHz, $CDCl_3$)
0.99
1.00
0.97
1.02
1.02
0.98
6.03
3.05
2.92
f1 (ppm)

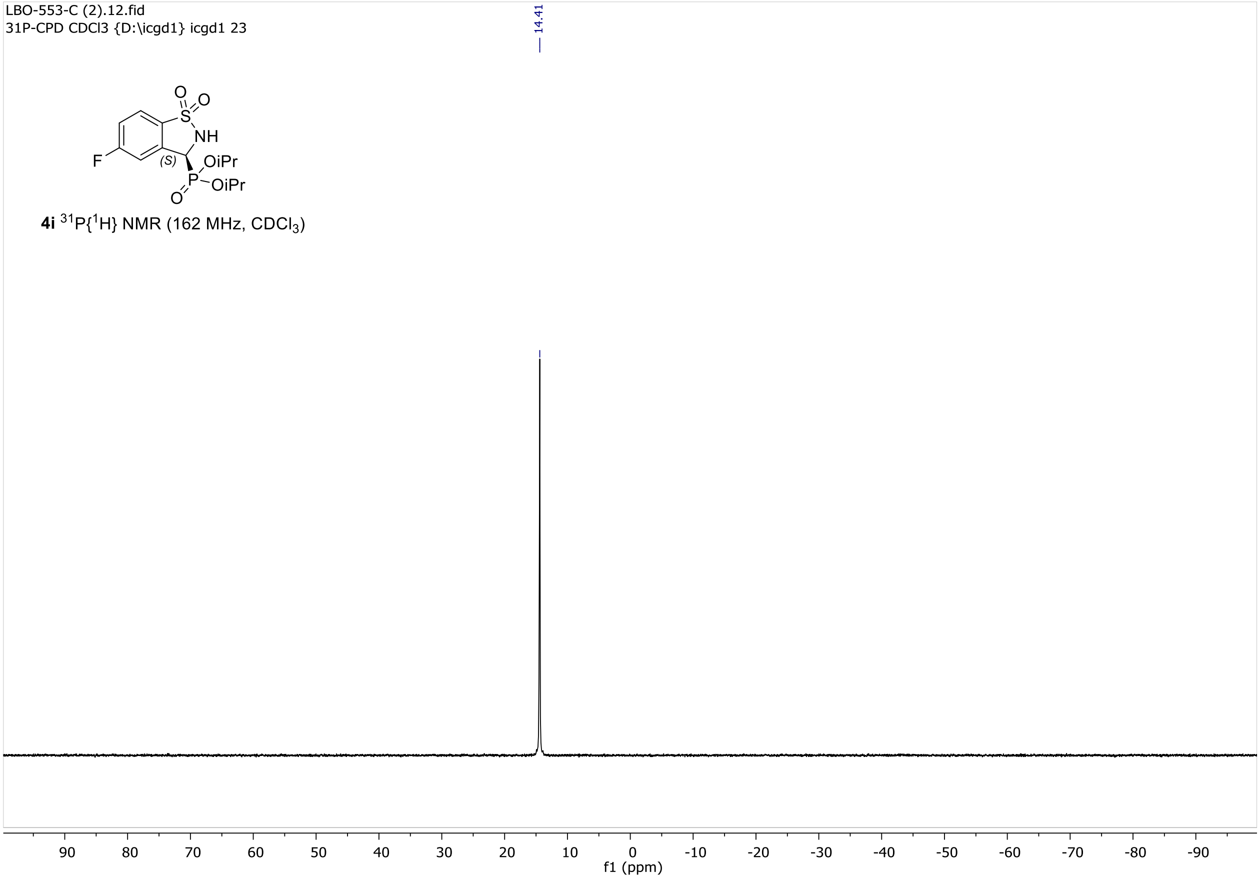
LBO-553-C (2).12.fid
31P-CPD CDCl3 {D:\icgd1} icgd1 23
14.41
4i ³¹P{¹H} NMR (162 MHz, CDCl3)
f1 (ppm)

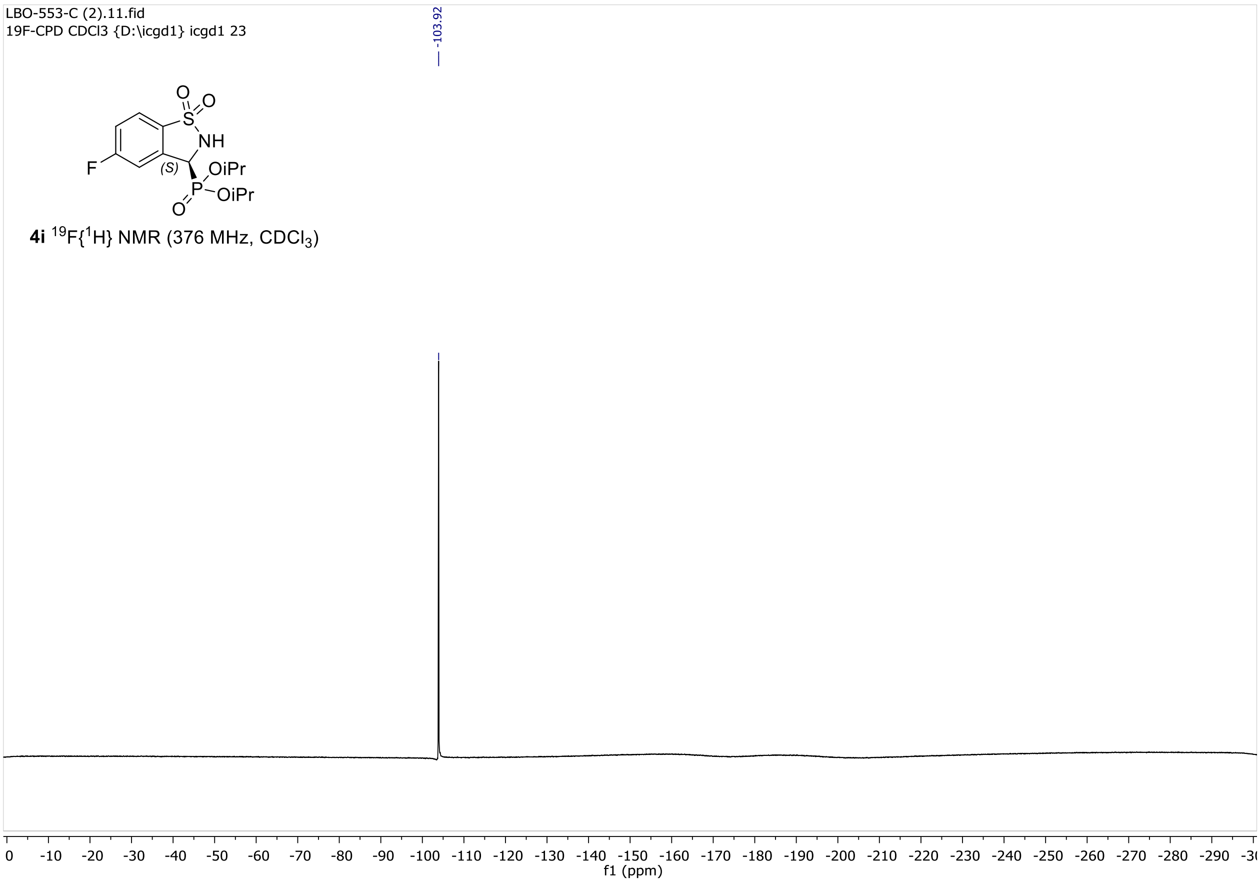
LBO-553-C (2).11.fid
19F-CPD CDCl3 {D:\icgd1} icgd1 23
-103.92
4i ¹⁹F{¹H} NMR (376 MHz, CDCl3)
f1 (ppm)

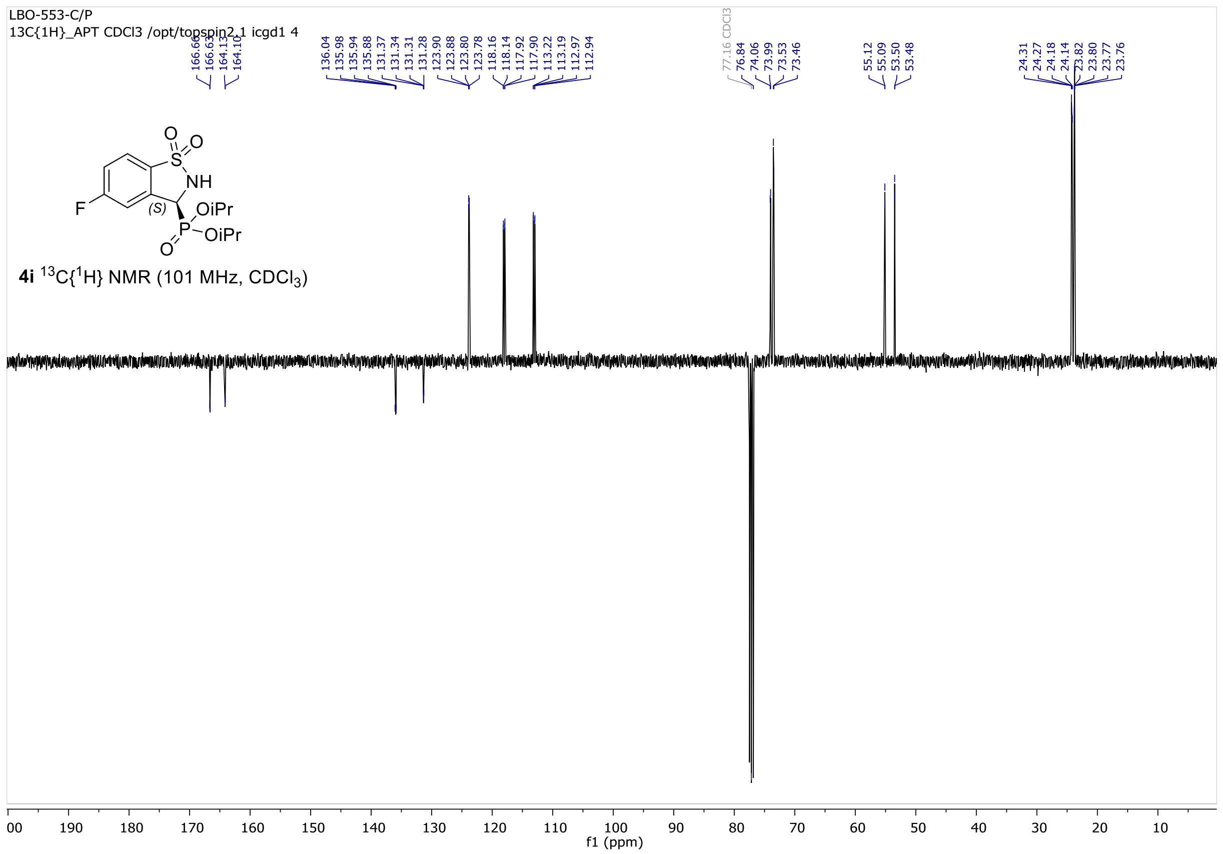

LBO-553-C/P
13C{1H}_APT CDCl3 /opt/topspin2.1 icgd1 4
4i 13C{1H} NMR (101 MHz, CDCl3)
f1 (ppm)

8. HPLC chromatogram of 2a-n and 4a-i

C:\...\Data\projet2\AM2N1\Louise\PPL-(8).lcd

Acquired by	: Admin
Sample Name	: PPL-(8)
Sample ID	:
Tray#	: 1
Vail #	: 31
Injection Volume	: 10 uL
Data File Name	: PPL-(8).lcd
Method File Name	: IC 85-15 30 min 1mLmir
Batch File Name	: echantillon chiraux scree
Report File Name	: Default.lcr
Data Acquired	: 04/10/2022 14:54:57
Data Processed	: 04/10/2022 15:24:59

Chromatogram
PPL-(8) C:\LabSolutions\Data\projet2\AM2N1\Louise\PPL-(8).lcd

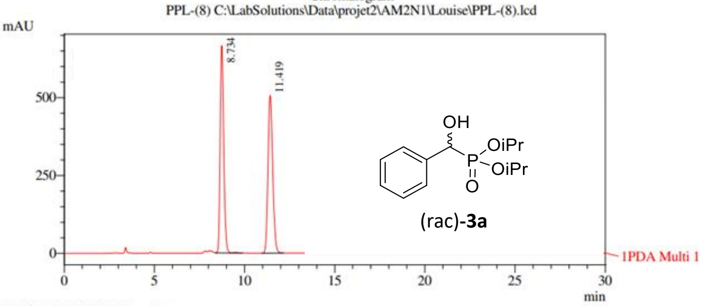

1 PDA Multi 1 / 214nm 4nm

PeakTable

PDA Ch1 214nm 4nm

Peak#	Ret. Time	Area	Height	Area %	Height %
1	8.734	9187112	665338	49.908	56.752
2	11.419	9221139	507014	50.092	43.248
Total		18408251	1172353	100.000	100.000

==== Shimadzu LCsolution Analysis Report ====

C:\LabSolutions\Data\projet2\AM2N1\Louise\PM18B.lcd

Acquired by	: Admin
Sample Name	: PM18B
Sample ID	:
Tray#	: 1
Vail #	: 17
Injection Volume	: 10 uL
Data File Name	: PM18B.lcd
Method File Name	: IC 85-15 30 min 1mLmin.lcm
Batch File Name	: echantillon chiraux taux catalytique.lcb
Report File Name	: Default.lcr
Data Acquired	: 26/07/2023 13:12:02
Data Processed	: 26/07/2023 13:42:05

<Chromatogram>

C:\LabSolutions\Data\projet2\AM2N1\Louise\PM18B.lcd

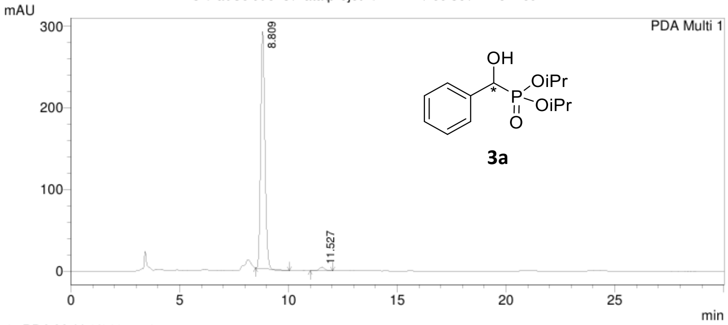

1 PDA Multi 1/210nm 4nm

PeakTable

PDA Ch1 210nm 4nm

Peak#	Ret. Time	Area	Height	Area %	Height %
1	8.809	3980905	290566	97.992	98.506
2	11.527	81560	4406	2.008	1.494
Total		4062465	294972	100.000	100.000

==== Shimadzu LCsolution Analysis Report ====

C:\LabSolutions\Data\projet2\AM2N1\Louise\PPL-(70)-rac.lcd

Acquired by	: Admin
Sample Name	: PPL-(70)-rac
Sample ID	:
Tray#	: 1
Vail #	: 76
Injection Volume	: 10 uL
Data File Name	: PPL-(70)-rac.lcd
Method File Name	: IA 65-35 30 min 0.7mLmin.lcm
Batch File Name	: Copie de racémiques scope.lcb
Report File Name	: Default.lcr
Data Acquired	: 28/06/2023 10:23:49
Data Processed	: 28/06/2023 10:53:53

<Chromatogram>

C:\LabSolutions\Data\projet2\AM2N1\Louise\PPL-(70)-rac.lcd

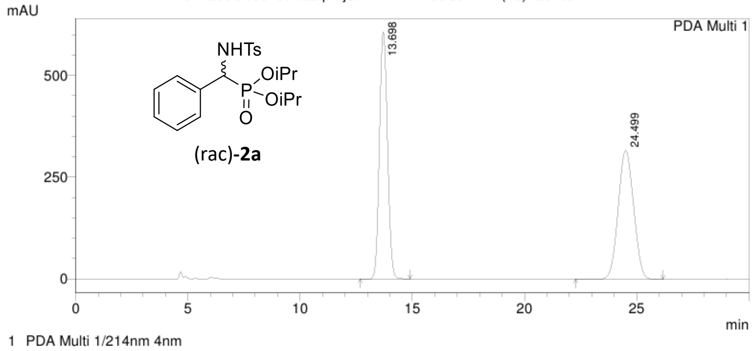

1 PDA Multi 1/214nm 4nm

PeakTable

PDA Ch1 214nm 4nm

Peak#	Ret. Time	Area	Height	Area %	Height %
1	13.698	15150618	606862	50.058	65.695
2	24.499	15115666	316902	49.942	34.305
Total		30266284	923764	100.000	100.000

==== Shimadzu LCsolution Analysis Report ====

C:\LabSolutions\Data\projet2\AM2N1\Louise\PM21F.lcd

Acquired by	: Admin
Sample Name	: PM21F
Sample ID	:
Tray#	: 1
Vail #	: 18
Injection Volume	: 10 uL
Data File Name	: PM21F.lcd
Method File Name	: IA 65-35 30 min 0.7mLmin.lcm
Batch File Name	: Copie de racémiques scope.lcb
Report File Name	: Default.lcr
Data Acquired	: 04/07/2023 12:51:02
Data Processed	: 04/07/2023 13:21:04

<Chromatogram>

C:\LabSolutions\Data\projet2\AM2N1\Louise\PM21F.lcd

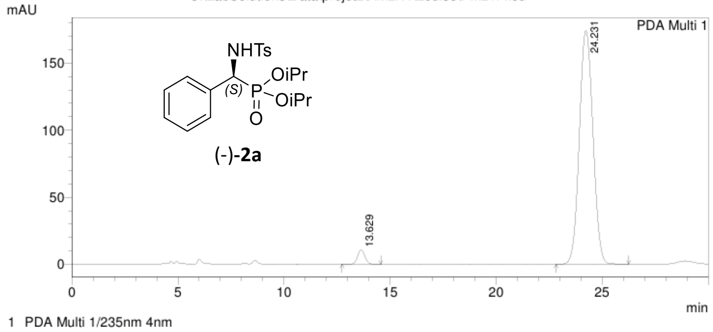

1 PDA Multi 1/235nm 4nm

PeakTable

PDA Ch1 235nm 4nm

Peak#	Ret. Time	Area	Height	Area %	Height %
1	13.629	256192	10654	3.210	5.766
2	24.231	7725973	174116	96.790	94.234
Total		7982165	184770	100.000	100.000

C:\LabSolutions\Data\projet2\AM2N1\Louise\PM009-rac-OMe.lcd

Acquired by	: Admin
Sample Name	: PM009-rac-OMe
Sample ID	:
Tray#	: 1
Vail #	: 5
Injection Volume	: 20 uL
Data File Name	: PM009-rac-OMe.lcd
Method File Name	: IA 65-35 30 min 0.7mLmin.lcm
Batch File Name	: echantillon chiraux taux catalytique.lcb
Report File Name	: Default.lcr
Data Acquired	: 28/07/2023 14:39:43
Data Processed	: 28/07/2023 15:09:45

<Chromatogram>

C:\LabSolutions\Data\projet2\AM2N1\Louise\PM009-rac-OMe.lcd

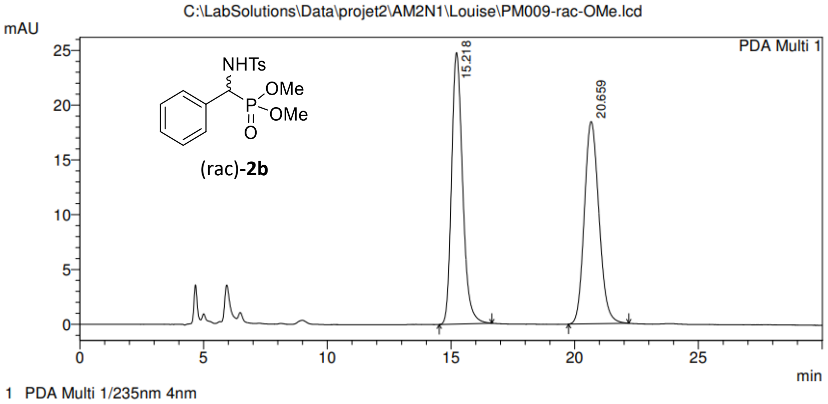

1 PDA Multi 1/235nm 4nm

PeakTable

PDA Ch1 235nm 4nm

Peak#	Ret. Time	Area	Height	Area %	Height %
1	15.218	767458	24782	50.195	57.302
2	20.659	761482	18466	49.805	42.698
Total		1528939	43248	100.000	100.000

C:\LabSolutions\Data\projet2\AM2N1\Louise\PM33A-OMe.lcd

Acquired by	: Admin
Sample Name	: PM33A-OMe
Sample ID	:
Tray#	: 1
Vail #	: 6
Injection Volume	: 10 uL
Data File Name	: PM33A-OMe.lcd
Method File Name	: IA 65-35 30 min 0.7mLmin.lcm
Batch File Name	: echantillon chiraux taux catalytique.lcb
Report File Name	: Default.lcr
Data Acquired	: 28/07/2023 15:29:20
Data Processed	: 28/07/2023 15:59:22

<Chromatogram>

C:\LabSolutions\Data\projet2\AM2N1\Louise\PM33A-OMe.lcd

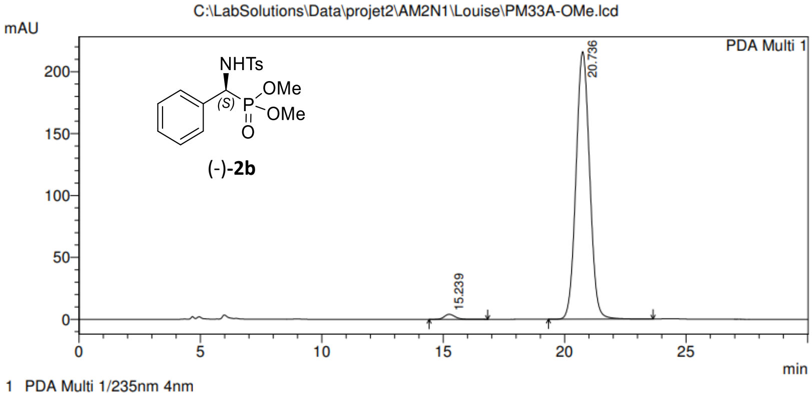

1 PDA Multi 1/235nm 4nm

PeakTable

PDA Ch1 235nm 4nm

Peak#	Ret. Time	Area	Height	Area %	Height %
1	15.239	127442	4058	1.493	1.844
2	20.736	8407616	215984	98.507	98.156
Total		8535058	220042	100.000	100.000

C:\LabSolutions\Data\projet2\AM2N1\Louise\PM17-rac-OEt.lcd

Acquired by	: Admin
Sample Name	: PM17-rac-OEt
Sample ID	:
Tray#	: 1
Vail #	: 3
Injection Volume	: 10 uL
Data File Name	: PM17-rac-OEt.lcd
Method File Name	: IA 65-35 30 min 0.7mLmin.lcm
Batch File Name	: echantillon chiraux taux catalytique.lcb
Report File Name	: Default.lcr
Data Acquired	: 28/07/2023 12:38:31
Data Processed	: 28/07/2023 13:08:34

Chromatogram

PM17-rac-OEt C:\LabSolutions\Data\projet2\AM2N1\Louise\PM17-rac-OEt.lcd

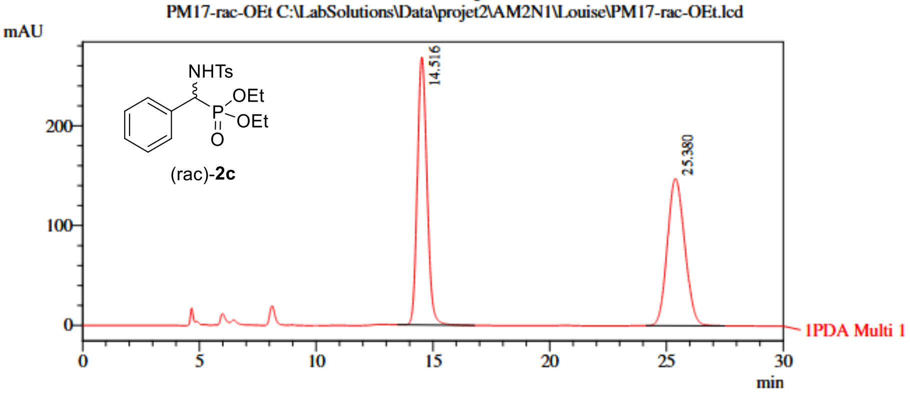

1 PDA Multi 1 / 214nm 4nm

PeakTable

PDA Ch1 214nm 4nm

Peak#	Ret. Time	Area	Height	Area %	Height %
1	14.516	7676569	268317	50.116	64.522
2	25.380	7640977	147539	49.884	35.478
Total		15317547	415856	100.000	100.000

C:\LabSolutions\Data\projet2\AM2N1\Louise\PM33B-OEt.lcd

Acquired by	: Admin
Sample Name	: PM33B-OEt
Sample ID	:
Tray#	: 1
Vail #	: 4
Injection Volume	: 10 uL
Data File Name	: PM33B-OEt.lcd
Method File Name	: IA 65-35 30 min 0.7mLmin.lcm
Batch File Name	: echantillon chiraux taux catalytique.lcb
Report File Name	: Default.lcr
Data Acquired	: 28/07/2023 13:39:07
Data Processed	: 28/07/2023 14:09:09

<Chromatogram>

C:\LabSolutions\Data\projet2\AM2N1\Louise\PM33B-OEt.lcd

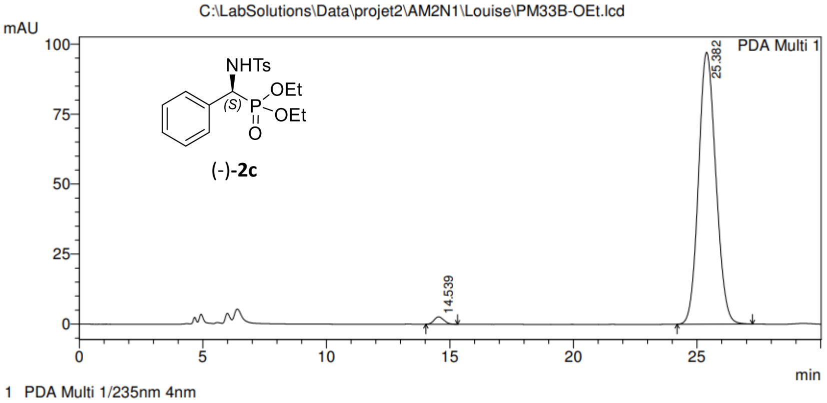

1 PDA Multi 1/235nm 4nm

PeakTable

PDA Ch1 235nm 4nm

Peak#	Ret. Time	Area	Height	Area %	Height %
1	14.539	72981	2626	1.520	2.633
2	25.382	4728338	97106	98.480	97.367
Total		4801320	99732	100.000	100.000

C:\LabSolutions\Data\projet2\AM2N1\Louise\PM6-rac-OBn.lcd

Acquired by	: Admin
Sample Name	: PM6-rac-OBn
Sample ID	:
Tray#	: 1
Vail #	: 1
Injection Volume	: 10 uL
Data File Name	: PM6-rac-OBn.lcd
Method File Name	: IA 65-35 40 min 0.7mlmin.lcm
Batch File Name	: echantillon chiraux taux catalytique.lcb
Report File Name	: Default.lcr
Data Acquired	: 28/07/2023 10:17:20
Data Processed	: 28/07/2023 10:57:22

<Chromatogram>

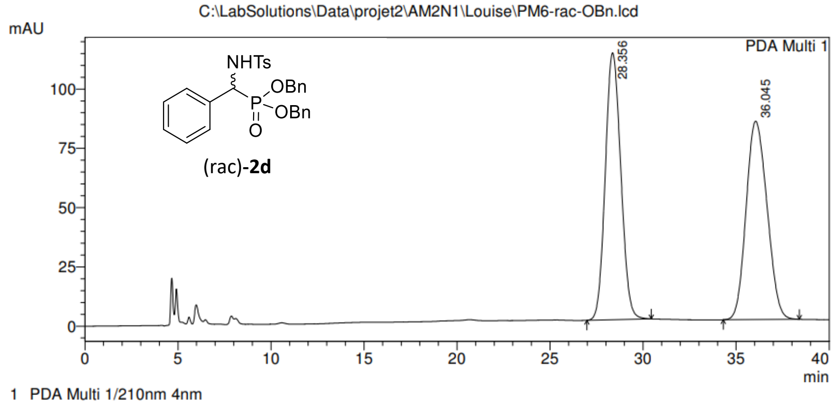

1 PDA Multi 1/210nm 4nm

PeakTable

PDA Ch1 210nm 4nm

Peak#	Ret. Time	Area	Height	Area %	Height %
1	28.356	6540294	112599	49.956	57.373
2	36.045	6551692	83659	50.044	42.627
Total		13091986	196258	100.000	100.000

C:\LabSolutions\Data\projet2\AM2N1\Louise\PM33C-OBn.lcd

Acquired by	: Admin
Sample Name	: PM33C-OBn
Sample ID	:
Tray#	: 1
Vail #	: 2
Injection Volume	: 10 uL
Data File Name	: PM33C-OBn.lcd
Method File Name	: IA 65-35 40 min 0.7mlmin.lcm
Batch File Name	: echantillon chiraux taux catalytique.lcb
Report File Name	: Default.lcr
Data Acquired	: 28/07/2023 11:27:56
Data Processed	: 28/07/2023 12:07:58

<Chromatogram>

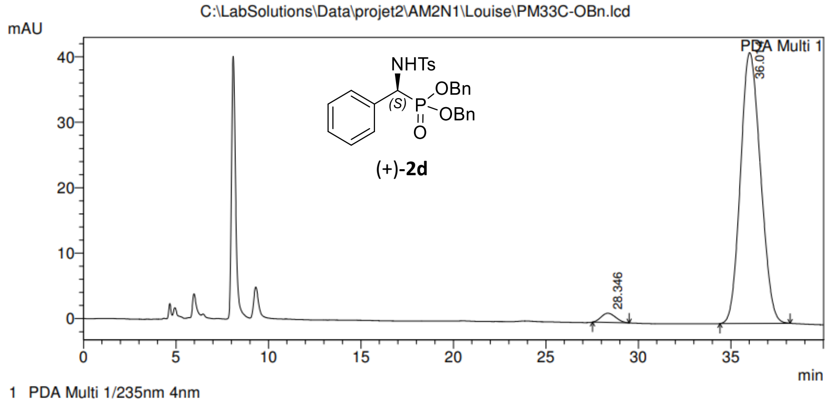

1 PDA Multi 1/235nm 4nm

PeakTable

PDA Ch1 235nm 4nm

Peak#	Ret. Time	Area	Height	Area %	Height %
1	28.346	77059	1407	2.411	3.288
2	36.014	3119601	41379	97.589	96.712
Total		3196660	42786	100.000	100.000

C:\LabSolutions\Data\projet2\AM2N1\Louise\rac 4-Me PPL-97 .lcd

Acquired by	: Admin
Sample Name	: rac 4-Me PPL-97
Sample ID	:
Tray#	: 1
Vail #	: 16
Injection Volume	: 10 uL
Data File Name	: rac 4-Me PPL-97 .lcd
Method File Name	: IA 65-35 30 min 0.7mLmin.lcm
Batch File Name	: Batch Paul muller.lcb
Report File Name	: Default.lcr
Data Acquired	: 14/05/2024 14:26:58
Data Processed	: 14/05/2024 14:57:01

Chromatogram
rac 4-Me PPL-97 C:\LabSolutions\Data\projet2\AM2N1\Louise\rac 4-Me PPL-97 .lcd

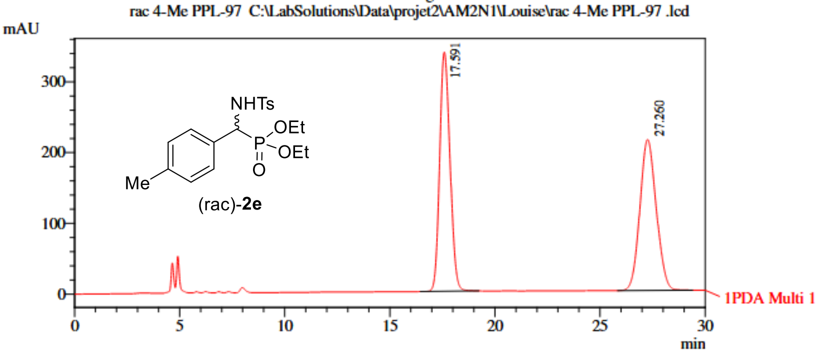

1 PDA Multi 1 / 210nm 4nm

PeakTable

PDA Ch1 210nm 4nm

Peak#	Ret. Time	Area	Height	Area %	Height %
1	17.591	11600391	338142	50.070	61.320
2	27.260	11567847	213296	49.930	38.680
Total		23168238	551438	100.000	100.000

C:\LabSolutions\Data\projet2\AM2N1\Louise\4-Me ATH rc023.lcd

Acquired by	: Admin
Sample Name	: 4-Me ATH rc023
Sample ID	:
Tray#	: 1
Vail #	: 17
Injection Volume	: 10 uL
Data File Name	: 4-Me ATH rc023.lcd
Method File Name	: IA 65-35 30 min 0.7mLmin.lcm
Batch File Name	: Batch Paul muller.lcb
Report File Name	: Default.lcr
Data Acquired	: 13/05/2024 12:39:31
Data Processed	: 13/05/2024 13:09:33

Chromatogram
4-Me ATH rc023 C:\LabSolutions\Data\projet2\AM2N1\Louise\4-Me ATH rc023.lcd

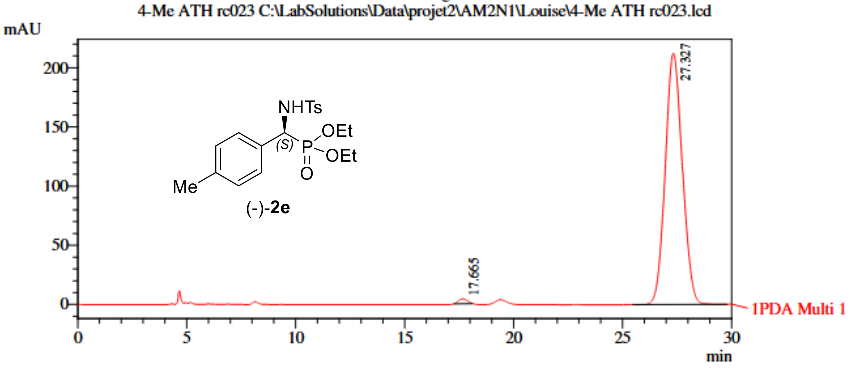

1 PDA Multi 1 / 230nm 4nm

PeakTable

PDA Ch1 230nm 4nm

Peak#	Ret. Time	Area	Height	Area %	Height %
1	17.665	120137	4126	1.026	1.907
2	27.327	11593979	212201	98.974	98.093
Total		11714116	216327	100.000	100.000

C:\LabSolutions\Data\projet2\AM2N1\Louise\RC073 3-Me rac .lcd

Acquired by	: Admin
Sample Name	: RC073 3-Me rac
Sample ID	:
Tray#	: 1
Vail #	: 39
Injection Volume	: 15 uL
Data File Name	: RC073 3-Me rac .lcd
Method File Name	: IA 65-35 40 min 0.7mlmin.lcm
Batch File Name	: Batch Paul muller.lcb
Report File Name	: Default.lcr
Data Acquired	: 25/06/2024 10:28:50
Data Processed	: 25/06/2024 11:08:53

Chromatogram
RC073 3-Me rac C:\LabSolutions\Data\projet2\AM2N1\Louise\RC073 3-Me rac .lcd

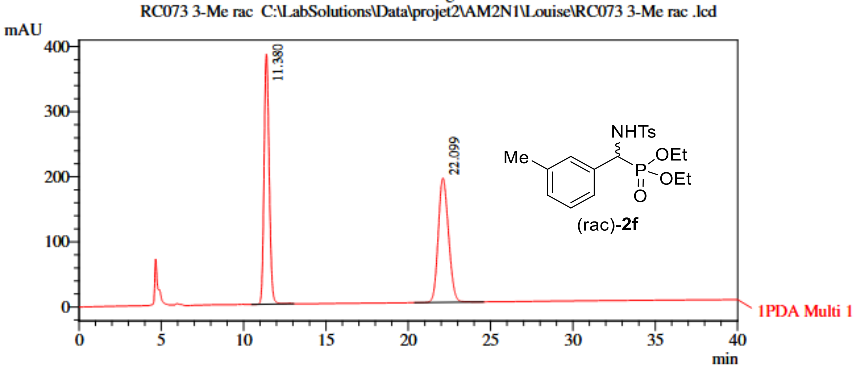

1 PDA Multi 1 / 210nm 4nm

PeakTable

PDA Ch1 210nm 4nm

Peak#	Ret. Time	Area	Height	Area %	Height %
1	11.380	8308654	384026	50.025	66.802
2	22.099	8300431	190843	49.975	33.198
Total		16609085	574869	100.000	100.000

C:\LabSolutions\Data\projet2\AM2N1\Louise\RC081 3-Me ATH.lcd

Acquired by	: Admin
Sample Name	: RC081 3-Me ATH
Sample ID	:
Tray#	: 1
Vail #	: 40
Injection Volume	: 15 uL
Data File Name	: RC081 3-Me ATH.lcd
Method File Name	: IA 65-35 40 min 0.7mlmin.lcm
Batch File Name	: Batch Paul muller.lcb
Report File Name	: Default.lcr
Data Acquired	: 25/06/2024 11:49:26
Data Processed	: 25/06/2024 12:29:29

Chromatogram
RC081 3-Me ATH C:\LabSolutions\Data\projet2\AM2N1\Louise\RC081 3-Me ATH.lcd

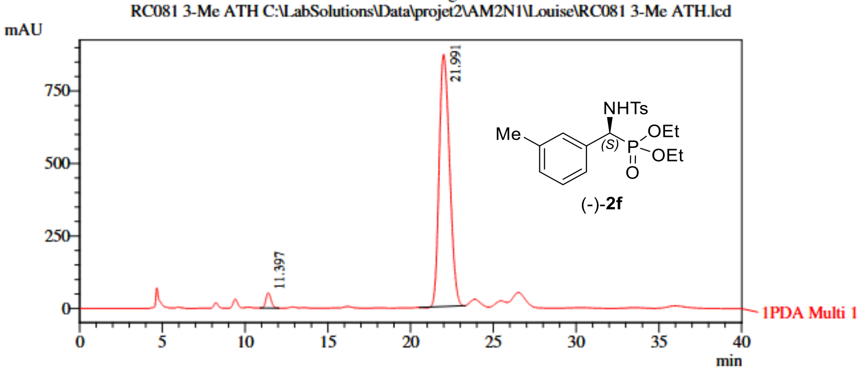

1 PDA Multi 1 / 210nm 4nm

PeakTable

PDA Ch1 210nm 4nm

Peak#	Ret. Time	Area	Height	Area %	Height %
1	11.397	1130026	52399	2.875	5.680
2	21.991	38173120	870084	97.125	94.320
Total		39303147	922484	100.000	100.000

C:\LabSolutions\Data\projet2\AM2N1\Louise\RC071 4-F rac .lcd

Acquired by	: Admin
Sample Name	: RC071 4-F rac
Sample ID	:
Tray#	: 1
Vail #	: 35
Injection Volume	: 15 uL
Data File Name	: RC071 4-F rac .lcd
Method File Name	: IA 65-35 40 min 0.7mlmin.lcm
Batch File Name	: Batch Paul muller.lcb
Report File Name	: Default.lcr
Data Acquired	: 14/06/2024 11:06:48
Data Processed	: 14/06/2024 11:46:52

Chromatogram

RC071 4-F rac C:\LabSolutions\Data\projet2\AM2N1\Louise\RC071 4-F rac .lcd

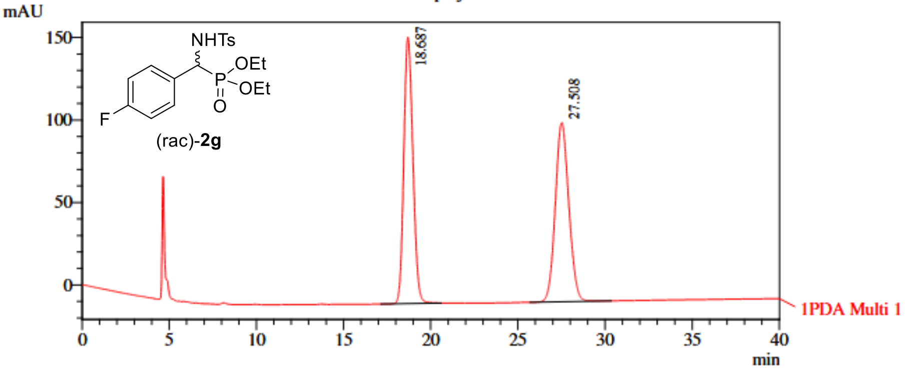

1 PDA Multi 1 / 210nm 4nm

PeakTable

PDA Ch1 210nm 4nm

Peak#	Ret. Time	Area	Height	Area %	Height %
1	18.687	5861671	161474	49.963	59.775
2	27.508	5870433	108662	50.037	40.225
Total		11732104	270136	100.000	100.000

C:\LabSolutions\Data\projet2\AM2N1\Louise\RC078 4-F ATH.lcd

Acquired by	: Admin
Sample Name	: RC078 4-F ATH
Sample ID	:
Tray#	: 1
Vail #	: 36
Injection Volume	: 15 uL
Data File Name	: RC078 4-F ATH.lcd
Method File Name	: IA 65-35 40 min 0.7mlmin.lcm
Batch File Name	: Batch Paul muller.lcb
Report File Name	: Default.lcr
Data Acquired	: 14/06/2024 12:27:23
Data Processed	: 14/06/2024 13:07:25

Chromatogram

RC078 4-F ATH C:\LabSolutions\Data\projet2\AM2N1\Louise\RC078 4-F ATH.lcd

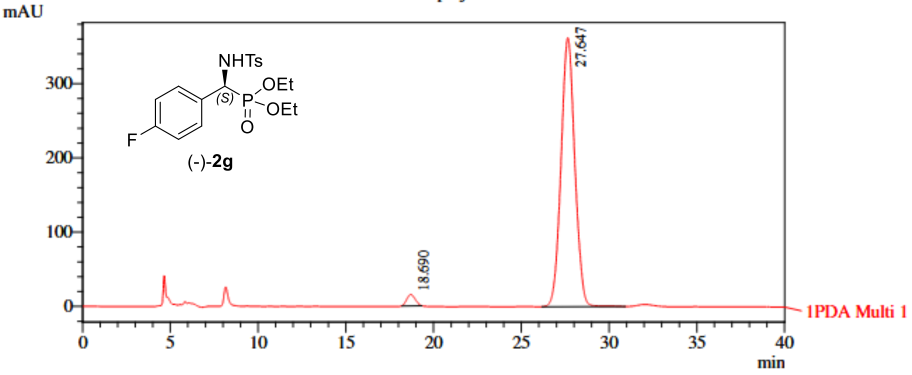

1 PDA Multi 1 / 214nm 4nm

PeakTable

PDA Ch1 214nm 4nm

Peak#	Ret. Time	Area	Height	Area %	Height %
1	18.690	525104	15477	2.585	4.097
2	27.647	19785400	362282	97.415	95.903
Total		20310504	377759	100.000	100.000

C:\LabSolutions\Data\projet2\AM2N1\Louise\RC049 4-Br rac .lcd

Acquired by	: Admin
Sample Name	: RC049 4-Br rac
Sample ID	:
Tray#	: 1
Vail #	: 33
Injection Volume	: 15 uL
Data File Name	: RC049 4-Br rac .lcd
Method File Name	: IA 65-35 50min 0.7ml min.lcm
Batch File Name	: Batch Paul muller.lcb
Report File Name	: Default.lcr
Data Acquired	: 30/05/2024 10:01:16
Data Processed	: 30/05/2024 10:51:20

Chromatogram

RC049 4-Br rac C:\LabSolutions\Data\projet2\AM2N1\Louise\RC049 4-Br rac .lcd

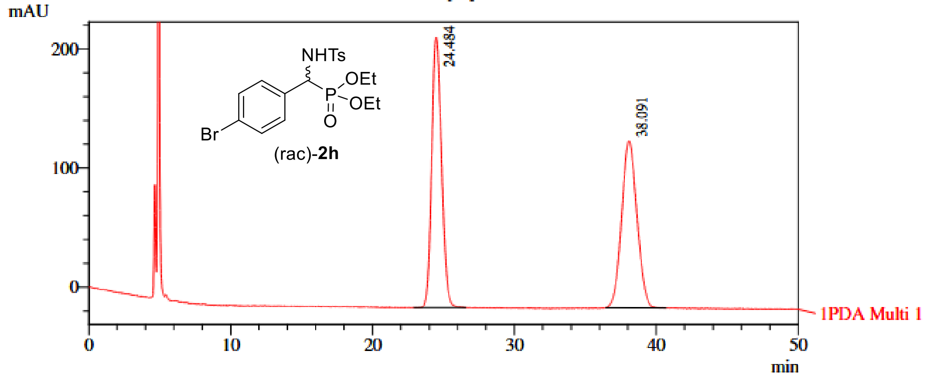

1 PDA Multi 1 / 195nm 4nm

PeakTable

PDA Ch1 195nm 4nm

Peak#	Ret. Time	Area	Height	Area %	Height %
1	24.484	10952729	227075	50.933	61.846
2	38.091	10551614	140089	49.067	38.154
Total		21504343	367164	100.000	100.000

C:\LabSolutions\Data\projet2\AM2N1\Louise\RC058 4-Br ATH.lcd

Acquired by	: Admin
Sample Name	: RC058 4-Br ATH
Sample ID	:
Tray#	: 1
Vail #	: 34
Injection Volume	: 15 uL
Data File Name	: RC058 4-Br ATH.lcd
Method File Name	: IA 65-35 50min 0.7ml min.lcm
Batch File Name	: Batch Paul muller.lcb
Report File Name	: Default.lcr
Data Acquired	: 30/05/2024 11:21:53
Data Processed	: 30/05/2024 12:11:55

Chromatogram

RC058 4-Br ATH C:\LabSolutions\Data\projet2\AM2N1\Louise\RC058 4-Br ATH.lcd

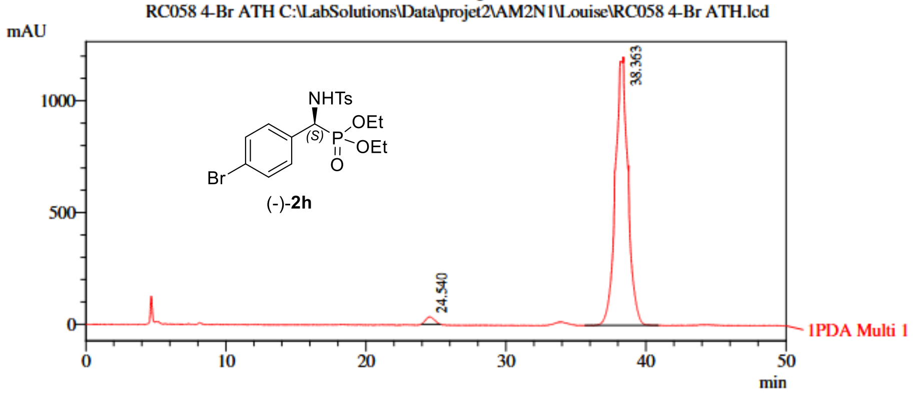

1 PDA Multi 1 / 190nm 4nm

PeakTable

PDA Ch1 190nm 4nm

Peak#	Ret. Time	Area	Height	Area %	Height %
1	24.540	1412185	33586	1.844	2.719
2	38.363	75174991	1201854	98.156	97.281
Total		76587175	1235440	100.000	100.000

C:\LabSolutions\Data\projet2\AM2N1\Louise\RC052 3-F rac .lcd

Acquired by	: Admin
Sample Name	: RC052 3-F rac
Sample ID	:
Tray#	: 1
Vail #	: 31
Injection Volume	: 15 uL
Data File Name	: RC052 3-F rac .lcd
Method File Name	: IA 65-35 40 min 0.7mlmin.lcm
Batch File Name	: Batch Paul muller.lcb
Report File Name	: Default.lcr
Data Acquired	: 06/06/2024 08:56:06
Data Processed	: 06/06/2024 09:21:22

Chromatogram

RC052 3-F rac C:\LabSolutions\Data\projet2\AM2N1\Louise\RC052 3-F rac .lcd

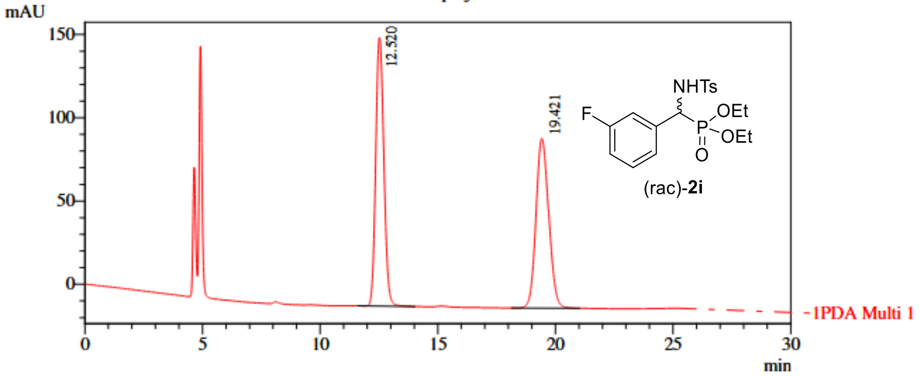

1 PDA Multi 1 / 214nm 4nm

PeakTable

PDA Ch1 214nm 4nm

Peak#	Ret. Time	Area	Height	Area %	Height %
1	12.520	3816618	161296	49.748	61.248
2	19.421	3855333	102051	50.252	38.752
Total		7671951	263346	100.000	100.000

C:\LabSolutions\Data\projet2\AM2N1\Louise\RC066 3-F ATH.lcd

Acquired by	: Admin
Sample Name	: RC066 3-F ATH
Sample ID	:
Tray#	: 1
Vail #	: 32
Injection Volume	: 15 uL
Data File Name	: RC066 3-F ATH.lcd
Method File Name	: IA 65-35 30 min 0.7mLmin.lcm
Batch File Name	: Batch Paul muller.lcb
Report File Name	: Default.lcr
Data Acquired	: 06/06/2024 09:52:05
Data Processed	: 06/06/2024 10:22:07

Chromatogram

RC066 3-F ATH C:\LabSolutions\Data\projet2\AM2N1\Louise\RC066 3-F ATH.lcd

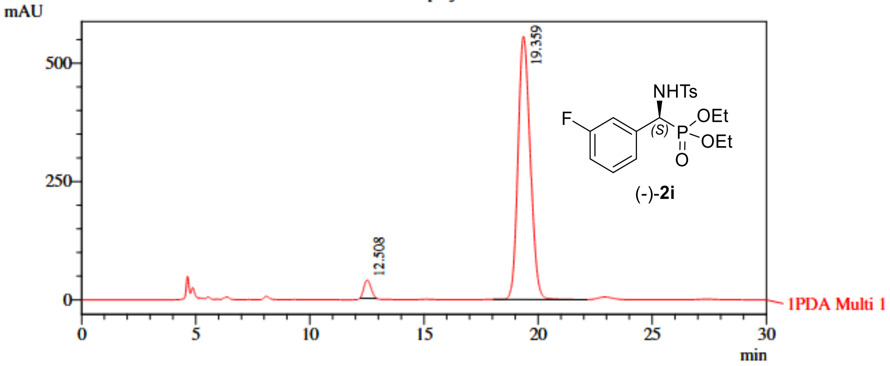

1 PDA Multi 1 / 214nm 4nm

PeakTable

PDA Ch1 214nm 4nm

Peak#	Ret. Time	Area	Height	Area %	Height %
1	12.508	831758	38755	3.841	6.516
2	19.359	20823298	556045	96.159	93.484
Total		21655056	594800	100.000	100.000

C:\LabSolutions\Data\projet2\AM2N1\Louise\RC072 2-F rac .lcd

Acquired by	: Admin
Sample Name	: RC072 2-F rac
Sample ID	:
Tray#	: 1
Vail #	: 37
Injection Volume	: 15 uL
Data File Name	: RC072 2-F rac .lcd
Method File Name	: IA 65-35 40 min 0.7mlmin.lcm
Batch File Name	: Batch Paul muller.lcb
Report File Name	: Default.lcr
Data Acquired	: 17/06/2024 12:44:08
Data Processed	: 17/06/2024 13:24:11

Chromatogram
RC072 2-F rac C:\LabSolutions\Data\projet2\AM2N1\Louise\RC072 2-F rac .lcd

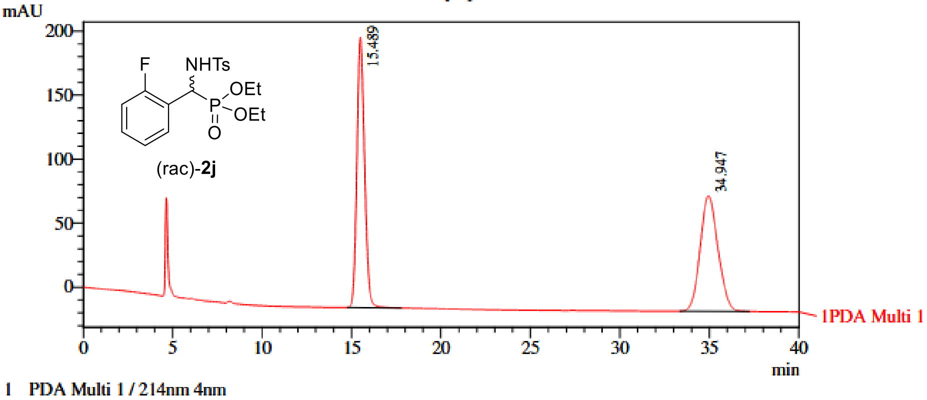

1 PDA Multi 1 / 214nm 4nm

PeakTable

PDA Ch1 214nm 4nm

Peak#	Ret. Time	Area	Height	Area %	Height %
1	15.489	6316832	211160	50.167	70.067
2	34.947	6274699	90209	49.833	29.933
Total		12591531	301369	100.000	100.000

C:\LabSolutions\Data\projet2\AM2N1\Louise\RC079 2-F ATH.lcd

Acquired by	: Admin
Sample Name	: RC079 2-F ATH
Sample ID	:
Tray#	: 1
Vail #	: 38
Injection Volume	: 15 uL
Data File Name	: RC079 2-F ATH.lcd
Method File Name	: IA 65-35 40 min 0.7mlmin.lcm
Batch File Name	: Batch Paul muller.lcb
Report File Name	: Default.lcr
Data Acquired	: 17/06/2024 14:04:43
Data Processed	: 17/06/2024 14:44:45

Chromatogram
RC079 2-F ATH C:\LabSolutions\Data\projet2\AM2N1\Louise\RC079 2-F ATH.lcd

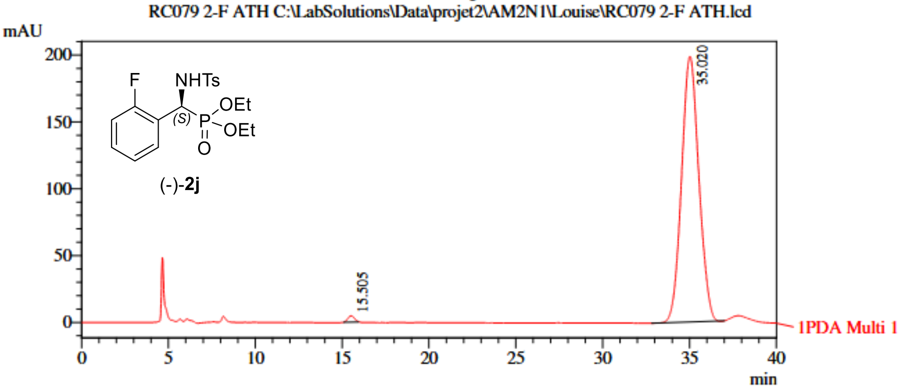

1 PDA Multi 1 / 214nm 4nm

PeakTable

PDA Ch1 214nm 4nm

Peak#	Ret. Time	Area	Height	Area %	Height %
1	15.505	127510	4738	0.922	2.330
2	35.020	13706549	198640	99.078	97.670
Total		13834058	203378	100.000	100.000

C:\LabSolutions\Data\projet2\AM2N1\Louise\RCxxx 4-CN rac .lcd

Acquired by	: Admin
Sample Name	: RCxxx 4-CN rac
Sample ID	:
Tray#	: 1
Vail #	: 43
Injection Volume	: 15 uL
Data File Name	: RCxxx 4-CN rac .lcd
Method File Name	: IA 65-35 60min 0.7ml min.lcm
Batch File Name	: Batch Paul muller.lcb
Report File Name	: Default.lcr
Data Acquired	: 27/06/2024 12:52:14
Data Processed	: 27/06/2024 13:52:16

Chromatogram
RCxxx 4-CN rac C:\LabSolutions\Data\projet2\AM2N1\Louise\RCxxx 4-CN rac .lcd

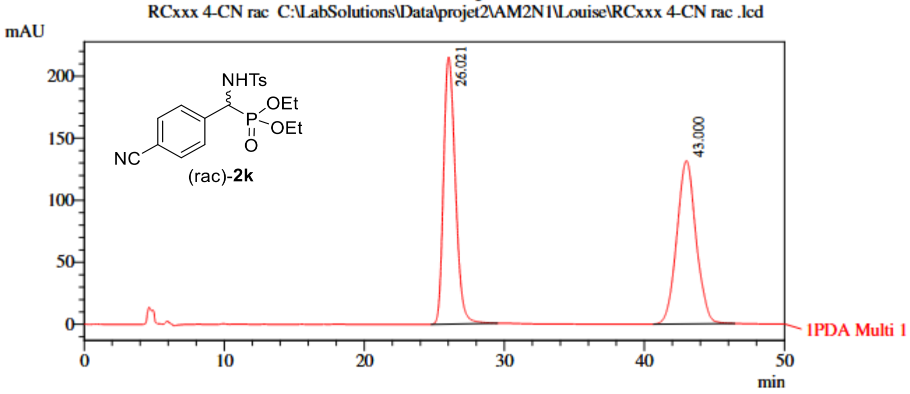

1 PDA Multi 1 / 214nm 4nm

PeakTable

PDA Ch1 214nm 4nm

Peak#	Ret. Time	Area	Height	Area %	Height %
1	26.021	12618748	215386	50.089	62.073
2	43.000	12573681	131601	49.911	37.927
Total		25192429	346987	100.000	100.000

C:\LabSolutions\Data\projet2\AM2N1\Louise\RC084 4-CN ATH.lcd

Acquired by	: Admin
Sample Name	: RC084 4-CN ATH
Sample ID	:
Tray#	: 1
Vail #	: 44
Injection Volume	: 15 uL
Data File Name	: RC084 4-CN ATH.lcd
Method File Name	: IA 65-35 60min 0.7ml min.lcm
Batch File Name	: Batch Paul muller.lcb
Report File Name	: Default.lcr
Data Acquired	: 27/06/2024 14:31:24
Data Processed	: 27/06/2024 15:31:28

Chromatogram
RC084 4-CN ATH C:\LabSolutions\Data\projet2\AM2N1\Louise\RC084 4-CN ATH.lcd

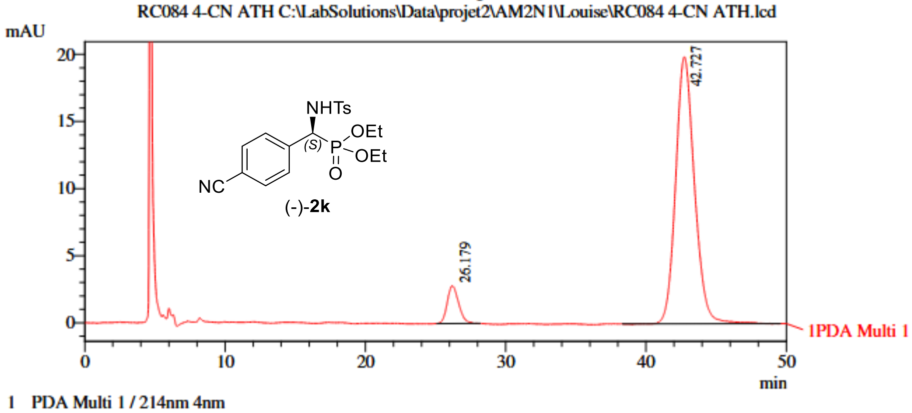

1 PDA Multi 1 / 214nm 4nm

PeakTable

PDA Ch1 214nm 4nm

Peak#	Ret. Time	Area	Height	Area %	Height %
1	26.179	159618	2800	7.912	12.337
2	42.727	1857719	19899	92.088	87.663
Total		2017337	22700	100.000	100.000

C:\LabSolutions\Data\projet2\AM2N1\Louise\RC064 3-CN rac .lcd

Acquired by	: Admin
Sample Name	: RC064 3-CN rac
Sample ID	:
Tray#	: 1
Vail #	: 41
Injection Volume	: 15 uL
Data File Name	: RC064 3-CN rac .lcd
Method File Name	: IA 65-35 60min 0.7ml min.lcm
Batch File Name	: Batch Paul muller.lcb
Report File Name	: Default.lcr
Data Acquired	: 27/06/2024 09:44:34
Data Processed	: 27/06/2024 10:17:31

Chromatogram

RC064 3-CN rac C:\LabSolutions\Data\projet2\AM2N1\Louise\RC064 3-CN rac .lcd

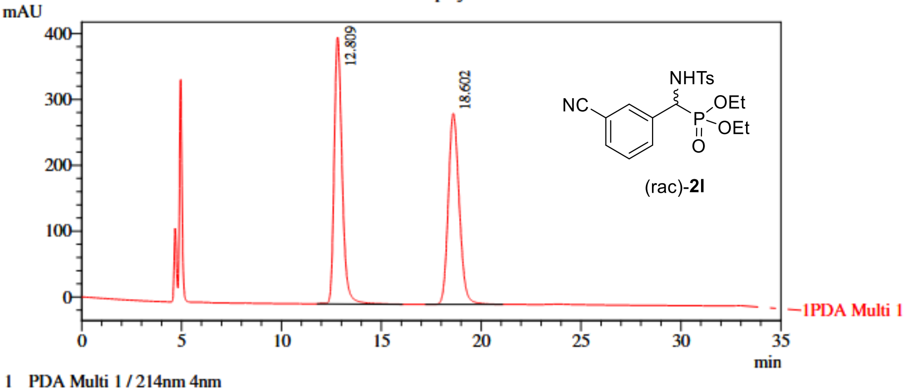

1 PDA Multi 1 / 214nm 4nm

PeakTable

PDA Ch1 214nm 4nm

Peak#	Ret. Time	Area	Height	Area %	Height %
1	12.809	11215550	405095	50.348	58.283
2	18.602	11060520	289948	49.652	41.717
Total		22276070	695043	100.000	100.000

C:\LabSolutions\Data\projet2\AM2N1\Louise\RC085 3-CN ATH.lcd

Acquired by	: Admin
Sample Name	: RC085 3-CN ATH
Sample ID	:
Tray#	: 1
Vail #	: 42
Injection Volume	: 15 uL
Data File Name	: RC085 3-CN ATH.lcd
Method File Name	: IA 65-35 30 min 0.7mLmin.lcm
Batch File Name	: Batch Paul muller.lcb
Report File Name	: Default.lcr
Data Acquired	: 27/06/2024 11:51:40
Data Processed	: 27/06/2024 12:21:40

Chromatogram

RC085 3-CN ATH C:\LabSolutions\Data\projet2\AM2N1\Louise\RC085 3-CN ATH.lcd

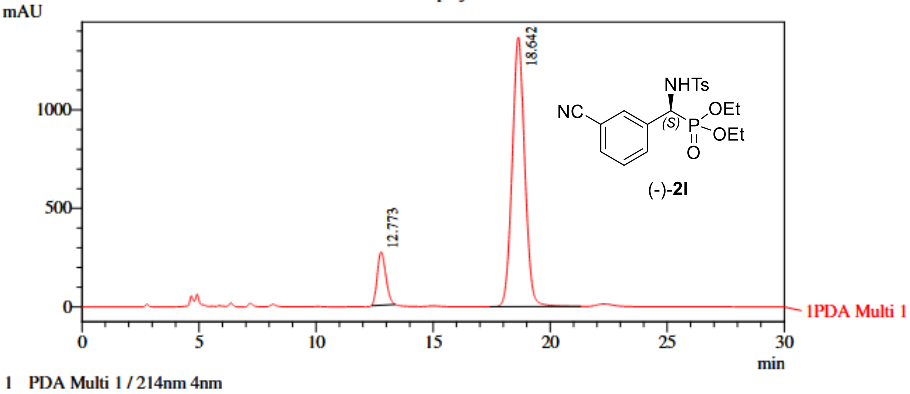

1 PDA Multi 1 / 214nm 4nm

PeakTable

PDA Ch1 214nm 4nm

Peak#	Ret. Time	Area	Height	Area %	Height %
1	12.773	6920817	270524	11.979	16.509
2	18.642	50854747	1368159	88.021	83.491
Total		57775564	1638683	100.000	100.000

C:\LabSolutions\Data\projet2\AM2N1\Louise\rac 4-NO2 PPL-102 .lcd

Acquired by	: Admin
Sample Name	: rac 4-NO2 PPL-102
Sample ID	:
Tray#	: 1
Vail #	: 18
Injection Volume	: 10 uL
Data File Name	: rac 4-NO2 PPL-102 .lcd
Method File Name	: IA 65-35 60min 0.7ml min.lcm
Batch File Name	: Batch Paul muller.lcb
Report File Name	: Default.lcr
Data Acquired	: 21/05/2024 10:43:09
Data Processed	: 21/05/2024 11:43:12

Chromatogram

rac 4-NO2 PPL-102 C:\LabSolutions\Data\projet2\AM2N1\Louise\rac 4-NO2 PPL-102 .lcd

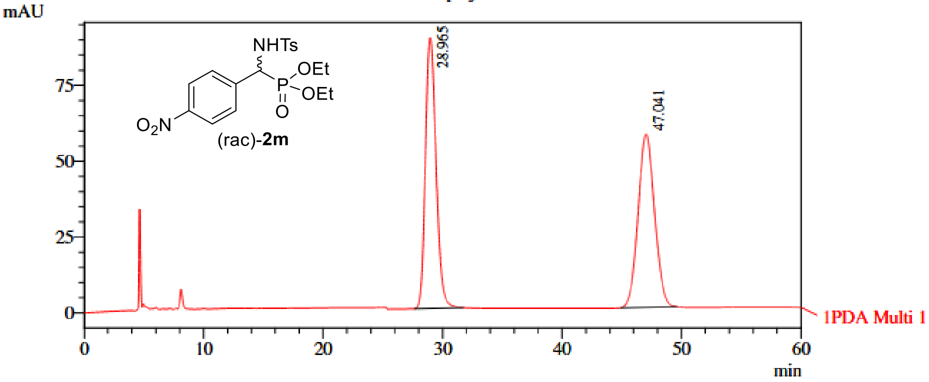

1 PDA Multi 1 / 214nm 4nm

PeakTable

PDA Ch1 214nm 4nm

Peak#	Ret. Time	Area	Height	Area %	Height %
1	28.965	5539347	89294	50.248	61.009
2	47.041	5484594	57068	49.752	38.991
Total		11023941	146361	100.000	100.000

C:\LabSolutions\Data\projet2\AM2N1\Louise\4-NO2 ATH rc-034.lcd

Acquired by	: Admin
Sample Name	: 4-NO2 ATH rc-034
Sample ID	:
Tray#	: 1
Vail #	: 19
Injection Volume	: 10 uL
Data File Name	: 4-NO2 ATH rc-034.lcd
Method File Name	: IA 65-35 60min 0.7ml min.lcm
Batch File Name	: Batch Paul muller.lcb
Report File Name	: Default.lcr
Data Acquired	: 13/05/2024 14:44:25
Data Processed	: 13/05/2024 15:44:28

Chromatogram

4-NO2 ATH rc-034 C:\LabSolutions\Data\projet2\AM2N1\Louise\4-NO2 ATH rc-034.lcd

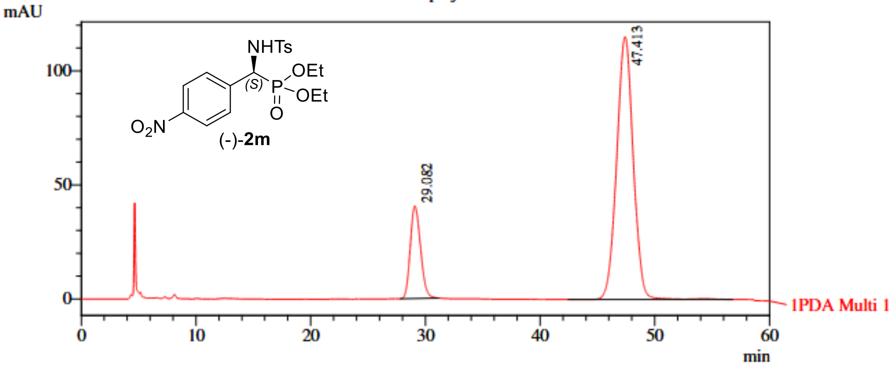

1 PDA Multi 1 / 214nm 4nm

PeakTable

PDA Ch1 214nm 4nm

Peak#	Ret. Time	Area	Height	Area %	Height %
1	29.082	2530480	40564	17.931	26.033
2	47.413	11581627	115255	82.069	73.967
Total		14112107	155819	100.000	100.000

==== Shimadzu LCsolution Analysis Report ====

C:\LabSolutions\Data\projet2\AM2N1\Louise\RC047 3-NO2 rac.lcd

Acquired by	: Admin
Sample Name	: RC047 3-NO2 rac
Sample ID	:
Tray#	: 1
Vail #	: 33
Injection Volume	: 15 uL
Data File Name	: RC047 3-NO2 rac.lcd
Method File Name	: IA 65-35 60min 0.7ml min.lcm
Batch File Name	: Batch Paul muller.lcb
Report File Name	: Default.lcr
Data Acquired	: 06/06/2024 09:52:41
Data Processed	: 06/06/2024 10:17:43

<Chromatogram>

C:\LabSolutions\Data\projet2\AM2N1\Louise\RC047 3-NO2 rac.lcd

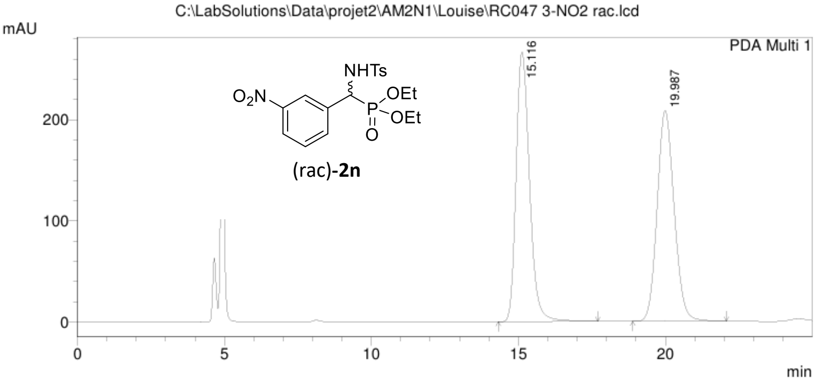

1 PDA Multi 1/214nm 4nm

PeakTable

PDA Ch1 214nm 4nm

Peak#	Ret. Time	Area	Height	Area %	Height %
1	15.116	8443466	266719	50.155	56.121
2	19.987	8391369	208541	49.845	43.879
Total		16834835	475261	100.000	100.000

==== Shimadzu LCsolution Analysis Report ====

C:\LabSolutions\Data\projet2\AM2N1\Louise\RC065 3-NO2 ATH.lcd

Acquired by	: Admin
Sample Name	: RC065 3-NO2 ATH
Sample ID	:
Tray#	: 1
Vail #	: 34
Injection Volume	: 15 uL
Data File Name	: RC065 3-NO2 ATH.lcd
Method File Name	: IA 65-35 30 min 0.7mLmin.lcm
Batch File Name	: Batch Paul muller.lcb
Report File Name	: Default.lcr
Data Acquired	: 06/06/2024 10:38:42
Data Processed	: 06/06/2024 13:00:52

<Chromatogram>

C:\LabSolutions\Data\projet2\AM2N1\Louise\RC065 3-NO2 ATH.lcd

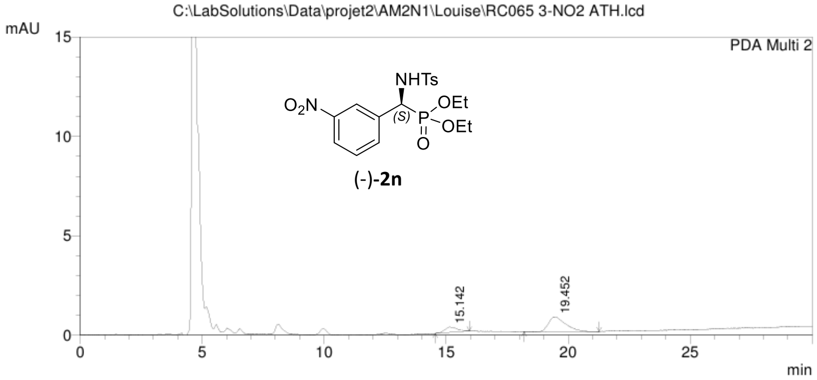

1 PDA Multi 2/210nm 4nm

PeakTable

PDA Ch2 210nm 4nm

Peak#	Ret. Time	Area	Height	Area %	Height %
1	15.142	9436	273	20.355	26.442
2	19.452	36924	760	79.645	73.558
Total		46360	1033	100.000	100.000

C:\LabSolutions\Data\projet2\AM2N1\Laura Borrel\Batch\Tests cata\ATH\LB-505 2 AD-H 20% 0.8 mL.min 8µL t.lcd

Sample Name	: LB-505 2 AD-H 20% 0.8 mL.min 8µL
Vail #	: 1
Injection Volume	: 8 uL
Data File Name	: LB-505 2 AD-H 20% 0.8 mL.min 8µL t.lcd
Method File Name	: I 80-20 30min voie 0 - 0.8 mL.min.lcm
Report File Name	: Default.lcr
Data Acquired	: 14/03/2025 09:43:46

C:\LabSolutions\Data\projet2\AM2N1\Laura Borrel\Batch\Tests cata\ATH\LB-505 2 AD-H 20% 0.8 mL.min 8µL t.lcd

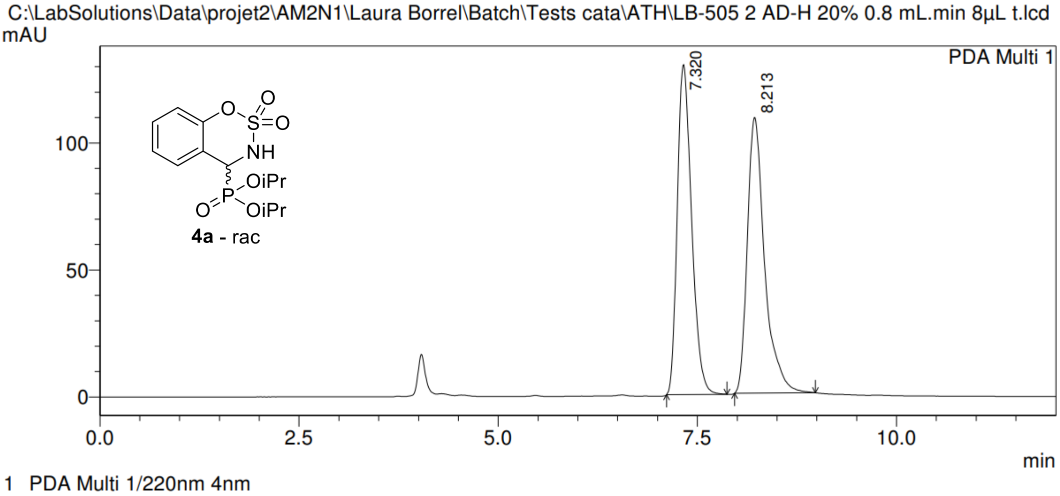

1 PDA Multi 1/220nm 4nm

PeakTable

PDA Ch1 220nm 4nm

Peak#	Ret. Time	Area	Height	Area %	Height %
1	7.320	1625421	129856	49.817	54.491
2	8.213	1637376	108450	50.183	45.509
Total		3262798	238306	100.000	100.000

C:\LabSolutions\Data\projet2\AM2N1\Laura Borrel\Batch\Tests cata\ATH\LB-517 AD-H 20% 0.8 mL.min 8µL t.lcd

Sample Name	: LB-517 AD-H 20% 0.8 mL.min 8µL
Vail #	: 2
Injection Volume	: 8 uL
Data File Name	: LB-517 AD-H 20% 0.8 mL.min 8µL t.lcd
Method File Name	: I 80-20 30min voie 0 - 0.8 mL.min.lcm
Report File Name	:
Data Acquired	: 14/03/2025 10:14:13

C:\LabSolutions\Data\projet2\AM2N1\Laura Borrel\Batch\Tests cata\ATH\LB-517 AD-H 20% 0.8 mL.min 8µL t.lcd

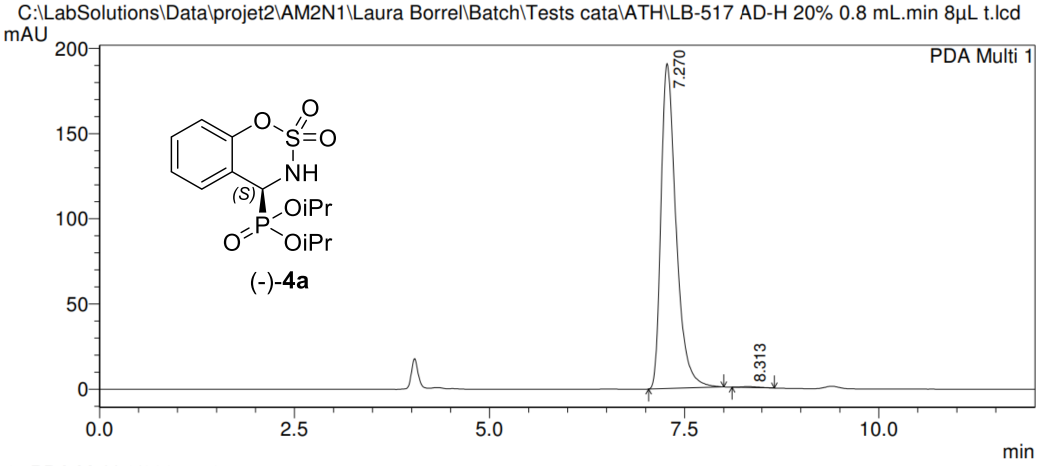

1 PDA Multi 1/220nm 4nm

PeakTable

PDA Ch1 220nm 4nm

Peak#	Ret. Time	Area	Height	Area %	Height %
1	7.270	2557626	190563	99.664	99.721
2	8.313	8625	534	0.336	0.279
Total		2566251	191097	100.000	100.000

F:\LabSolutions\Data\projet2\D1\David Virieux\Laura Borrel\Batch\LB-530 AD-H 10% 0.8 mL.min 8µL t2.lcd

Sample Name	: LB-530 AD-H 10% 0.8 mL.min 8µL
Vail #	: 1
Injection Volume	: 8 uL
Data File Name	: LB-530 AD-H 10% 0.8 mL.min 8µL t2.lcd
Method File Name	: I 90-10 40min voie 0 - 0.8mLmin.lcm
Report File Name	: Default.lcr
Data Acquired	: 14/04/2025 11:29:57

F:\LabSolutions\Data\projet2\D1\David Virieux\Laura Borrel\Batch\LB-530 AD-H 10% 0.8 mL.min 8µL t2.lcd

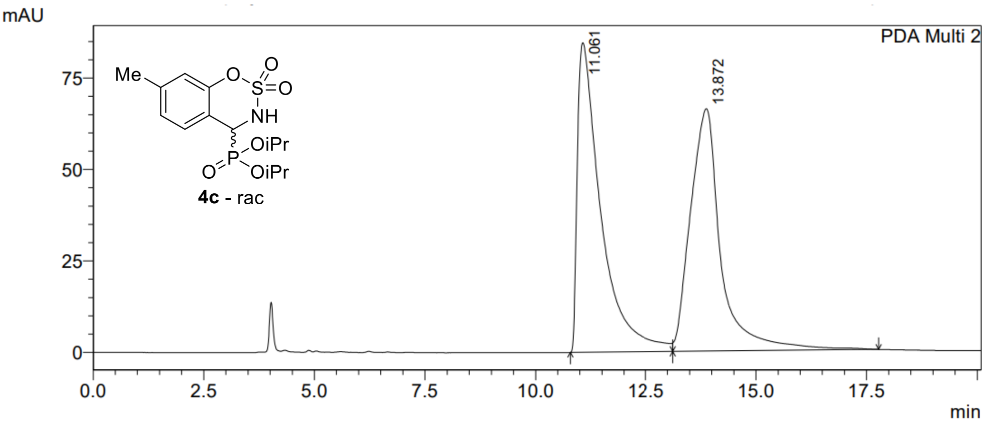

1 PDA Multi 2/220nm 4nm

PDA Ch2 220nm 4nm

Peak#	Ret. Time	Area	Height	Area %	Height %
1	11.061	3141065	84579	49.775	56.068
2	13.872	3169499	66272	50.225	43.932
Total		6310564	150851	100.000	100.000

C:\LabSolutions\Data\projet2\AM2N1\Laura Borrel\Batch\LB-535 AD-H 10% 0.8 mL.min 8µL t.lcd

Sample Name	: LB-535 AD-H 10% 0.8 mL.min 8µL
Vail #	: 7
Injection Volume	: 8 uL
Data File Name	: LB-535 AD-H 10% 0.8 mL.min 8µL t.lcd
Method File Name	: I 90-10 20min voie 0 - 0.8 mLmin.lcm
Report File Name	: Default.lcr
Data Acquired	: 14/04/2025 15:36:16

C:\LabSolutions\Data\projet2\AM2N1\Laura Borrel\Batch\LB-535 AD-H 10% 0.8 mL.min 8µL t.lcd

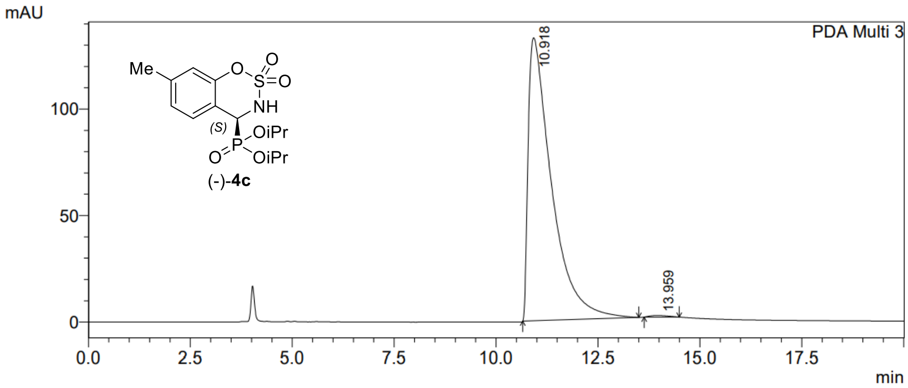

1 PDA Multi 3/220nm 4nm

PeakTable

PDA Ch3 220nm 4nm

Peak#	Ret. Time	Area	Height	Area %	Height %
1	10.918	5317042	132859	99.566	99.439
2	13.959	23173	750	0.434	0.561
Total		5340215	133610	100.000	100.000

Sample name: C6-OMe-rac
Column: Chiralpak IG
Temperature:
Mobile phase: Heptane/ethanol (80/20), 1 mL/min

Data file: C:\CHEM32\1\DATA\17-06-2025\C6-OME-RAC_IG_1.D
Injection date: 6/17/2025 12:57:59 PM **Injection volume:** 5.000
Acq. method: E-20-CD254NM.M **Analysis method:** C6-OME-E-20-CD254.M
Last changed: 6/18/2025 11:20:04 AM **Location:** Vial 51
Column void time (min)

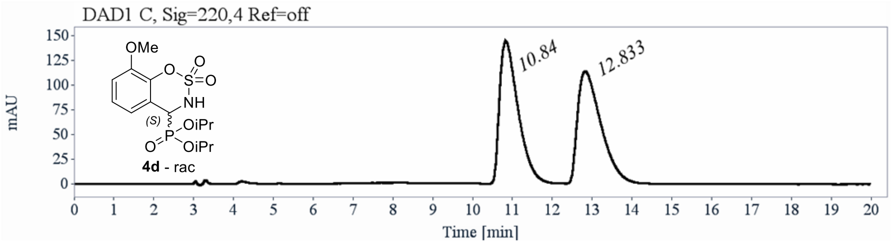

Signal: DAD1 C, Sig=220,4 Ref=off

RT [min]	Area	Area%	Capacity Factor	Enantioselectivity	Resolution (USP)
10.84	4723	50.06	2.67		
12.83	4712	49.94	3.35	1.25	2.07
Sum	9435	100.00			

Sample name: C6-OMe-ee
Column: Chiralpak IG
Temperature:
Mobile phase: Heptane/ethanol (80/20), 1 mL/min

Data file: C:\CHEM32\1\DATA\17-06-2025\C6-OME-EE_IG_2.D
Injection date: 6/17/2025 8:28:43 PM **Injection volume:** 5.000
Acq. method: E-20-CD254NM.M **Analysis method:** C6-OME-E-20-CD254.M
Last changed: 6/18/2025 11:20:04 AM **Location:** Vial 61
Column void time (min)

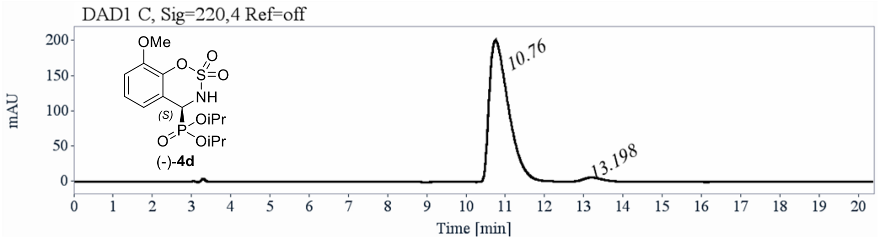

Signal: DAD1 C, Sig=220,4 Ref=off

RT [min]	Area	Area%	Capacity Factor	Enantioselectivity	Resolution (USP)
10.76	6916	96.40	2.65		
13.20	259	3.60	3.47	1.31	2.70
Sum	7175	100.00			

C6-mOMe-rac

Sample name:
Column: Chiralpak IF

Temperature:
Mobile phase: Heptane/ethanol (80/20), 1 mL/min

Data file: C:\CHEM32\1\DATA\17-06-2025\C6-MOME-RAC_IF_2.D
Injection date: 6/17/2025 2:36:17 PM **Injection volume:** 5.000
Acq. method: E-20-CD254NM.M **Analysis method:** C6-MOME-E-20-CD254.M

Last changed: 6/18/2025 11:19:05 AM **Location:** Vial 52
Column void time (min)

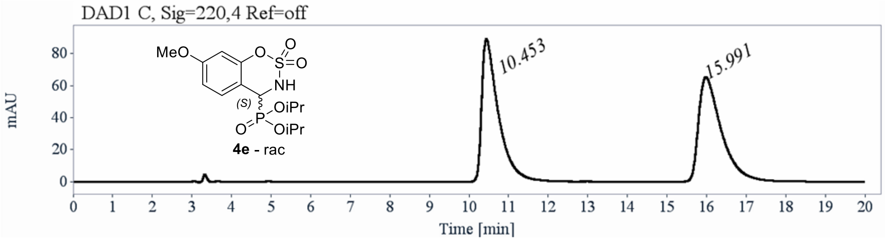

Signal: DAD1 C, Sig=220,4 Ref=off

RT [min]	Area	Area%	Capacity Factor	Enantioselectivity	Resolution (USP)
10.45	2634	49.92	2.54		
15.99	2642	50.08	4.42	1.74	6.39
Sum	5276	100.00			

C6-OMe-ee

Sample name:
Column: Chiralpak IG

Temperature:
Mobile phase: Heptane/ethanol (80/20), 1 mL/min

Data file: C:\CHEM32\1\DATA\17-06-2025\C6-OME-EE_IG_2.D
Injection date: 6/17/2025 8:28:43 PM **Injection volume:** 5.000
Acq. method: E-20-CD254NM.M **Analysis method:** C6-OME-E-20-CD254.M

Last changed: 6/18/2025 11:20:04 AM **Location:** Vial 61
Column void time (min)

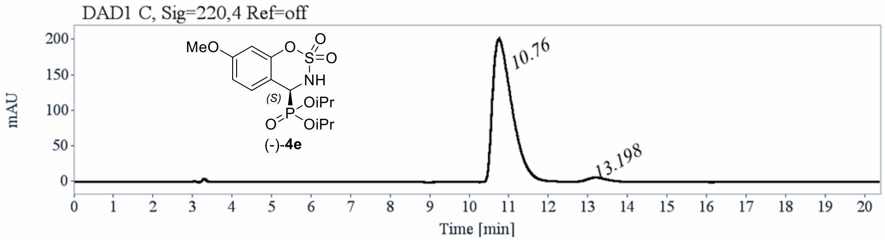

Signal: DAD1 C, Sig=220,4 Ref=off

RT [min]	Area	Area%	Capacity Factor	Enantioselectivity	Resolution (USP)
10.76	6916	96.40	2.65		
13.20	259	3.60	3.47	1.31	2.70
Sum	7175	100.00			

Sample name: C6-pOMe-rac
Column: Chiralpak IF
Temperature:
Mobile phase: Heptane/ethanol (80/20), 1 mL/min

Data file: C:\CHEM32\1\DATA\17-06-2025\C6-POME-RAC_IF_2.D
Injection date: 6/17/2025 2:57:46 PM **Injection volume:** 5.000
Acq. method: E-20-CD254NM.M **Analysis method:** C6-POME-E-20-CD254.M
Last changed: 6/18/2025 11:21:00 AM **Location:** Vial 53
Column void time (min)

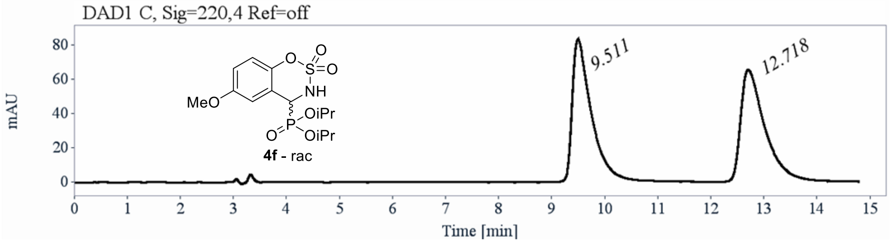

Signal: DAD1 C, Sig=220,4 Ref=off

RT [min]	Area	Area%	Capacity Factor	Enantioselectivity	Resolution (USP)
9.51	2143	50.50	2.22		
12.72	2100	49.50	3.31	1.49	4.54
Sum	4243	100.00			

Sample name: C6-pOMe-ee
Column: Chiralpak IF
Temperature:
Mobile phase: Heptane/ethanol (80/20), 1 mL/min

Data file: C:\CHEM32\1\DATA\17-06-2025\C6-POME-EE_IF_2.D
Injection date: 6/17/2025 4:56:33 PM **Injection volume:** 5.000
Acq. method: E-20-CD254NM.M **Analysis method:** C6-POME-E-20-CD254.M
Last changed: 6/18/2025 11:21:00 AM **Location:** Vial 63
Column void time (min)

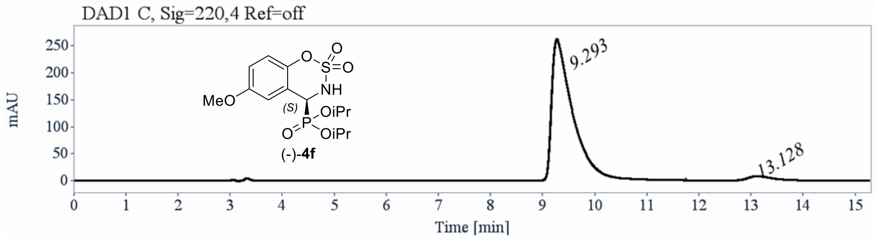

Signal: DAD1 C, Sig=220,4 Ref=off

RT [min]	Area	Area%	Capacity Factor	Enantioselectivity	Resolution (USP)
9.29	7495	96.13	2.15		
13.13	302	3.87	3.45	1.60	4.94
Sum	7796	100.00			

C:\LabSolutions\Data\projet2\AM2N1\Pierre-Olivier Butin\Opti C5 H\t BPO rac H-C5 OD 20%-0.8m.lcd

Sample Name	: BPO rac H-C5 OD 20%-0.8m
Vail #	: 1
Injection Volume	: 15 uL
Data File Name	: t BPO rac H-C5 OD 20%-0.8m.lcd
Method File Name	: OD- 80-20 25min 0.8 mL min 25 degrés.lcm
Report File Name	: Default.lcr
Data Acquired	: 05/05/2025 10:31:48

C:\LabSolutions\Data\projet2\AM2N1\Pierre-Olivier Butin\Opti C5 H\t BPO rac H-C5 OD 20%-0.8m.lcd

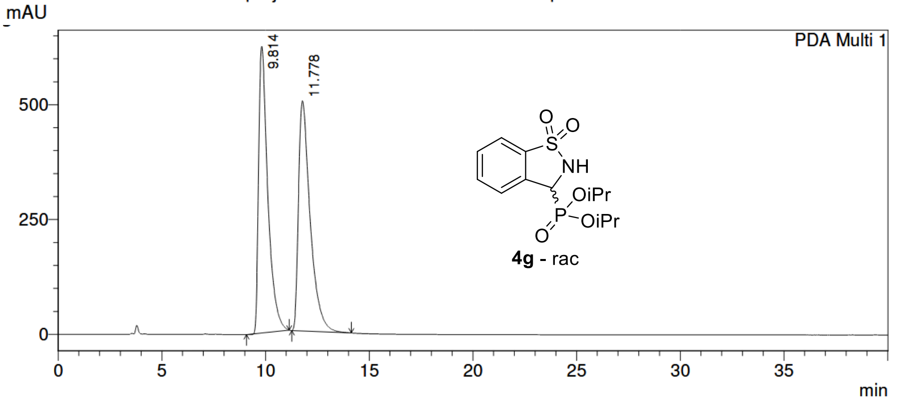

PeakTable

PDA Ch1 220nm 4nm

Peak#	Ret. Time	Area	Height	Area %
1	9.814	19313717	624300	49.613
2	11.778	19614806	501445	50.387
Total		38928523	1125745	100.000

C:\...\Data\Projet 2\D1\David Virieux\Pierre-Olivier Butin\Opti C5 H\t BPO-153-chir Ms TENEB OD 20%-0.8 mL.lcd

Sample Name	: BPO-153-chir Ms TENEB OD 20%-0.8 mL
Vail #	: 53
Injection Volume	: 7 uL
Data File Name	: t BPO-153-chir Ms TENEB OD 20%-0.8 mL.lcd
Method File Name	: OD- 80-20 25min 0.8 mL min 25 degrés.lcm
Report File Name	: Default.lcr
Data Acquired	: 05/05/2025 11:40:40

C:\...\Data\Projet 2\D1\David Virieux\Pierre-Olivier Butin\Opti C5 H\t BPO-153-chir Ms TENEB OD 20%-0.8 mL.lcd

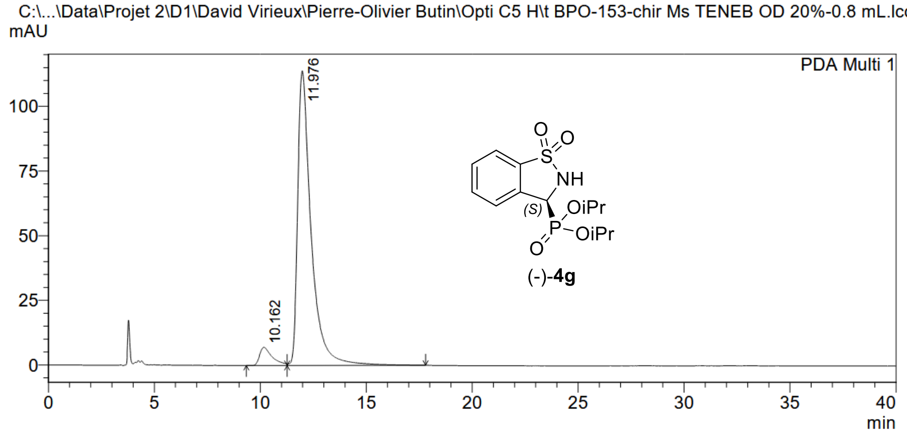

PeakTable

PDA Ch1 220nm 4nm

Peak#	Ret. Time	Area	Height	Area %
1	10.162	289705	7042	5.438
2	11.976	5037518	114004	94.562
Total		5327222	121046	100.000

Sample name: C5-Me-rac

Column: Chiralpak IK

Temperature:

Mobile phase: Heptane/ethanol (60/40), 1 mL/min

Data file: C:\CHEM32\1\DATA\17-06-2025\C5-ME-RAC_3.D

Injection date: 6/18/2025 10:44:07 AM **Injection volume:** 5.000

Acq. method: E-40-CD254NM.M **Analysis method:** C5-ME-E-40-CD254.M

Last changed: 6/18/2025 1:27:21 PM **Location:** Vial 54

Column void time (min)

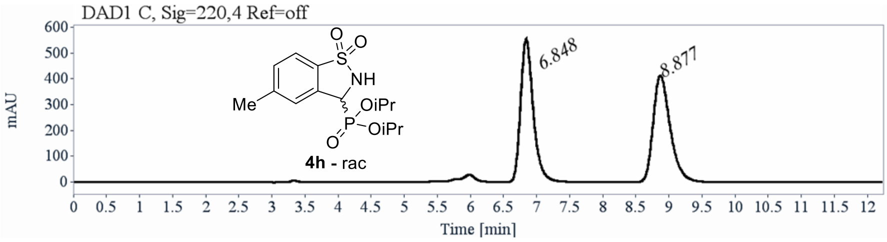

Signal: DAD1 C, Sig=220,4 Ref=off

RT [min]	Area	Area%	Capacity Factor	Enantioselectivity	Resolution (USP)
6.85	7422	49.86	1.32		
8.88	7463	50.14	2.01	1.52	5.07
Sum	14885	100.00			

Sample name: C5-Me-ee

Column: Chiralpak IK

Temperature:

Mobile phase: Heptane/ethanol (60/40), 1 mL/min

Data file: C:\CHEM32\1\DATA\17-06-2025\C5-ME-EE_4.D

Injection date: 6/18/2025 11:29:11 AM **Injection volume:** 5.000

Acq. method: E-40-CD254NM.M **Analysis method:** C5-ME-E-40-CD254.M

Last changed: 6/18/2025 1:27:21 PM **Location:** Vial 64

Column void time (min)

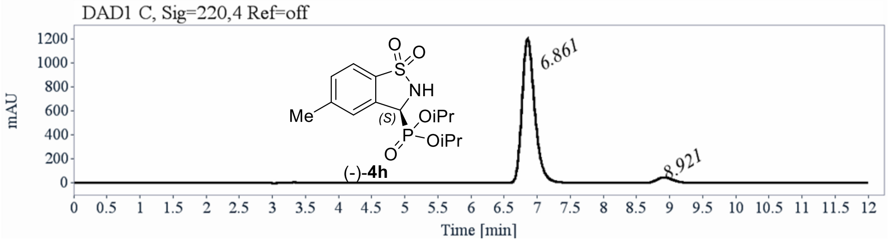

Signal: DAD1 C, Sig=220,4 Ref=off

RT [min]	Area	Area%	Capacity Factor	Enantioselectivity	Resolution (USP)
6.86	16178	94.95	1.33		
8.92	860	5.05	2.02	1.53	5.19
Sum	17038	100.00			

C:\LabSolutions\Data\Projet 2\D1\David Virieux\Laura Borrel\Batch\BPO-175 IB 12 0.8 mL.min 10µL t.lcd

Sample Name : BPO-175 IB 12 0.8 mL.min 10µL
Vail # : 1
Injection Volume : 10 uL
Data File Name : BPO-175 IB 12 0.8 mL.min 10µL t.lcd
Method File Name : I 88-12 40min voie 0 - 0.8 mLmin.lcm
Report File Name : Default.lcr
Data Acquired : 27/06/2025 10:39:39

C:\LabSolutions\Data\Projet 2\D1\David Virieux\Laura Borrel\Batch\BPO-175 IB 12 0.8 mL.min 10µL t.lcd

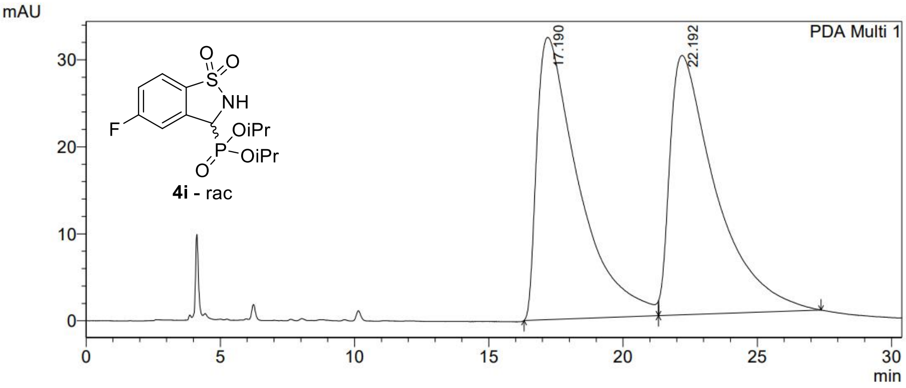

PeakTable

PDA Ch1 220nm 4nm

Peak#	Ret. Time	Area	Height	Area %	Height %
1	17.190	3516772	32451	49.686	52.107
2	22.192	3561252	29826	50.314	47.893
Total		7078024	62277	100.000	100.000

C:\LabSolutions\Data\Projet 2\D1\David Virieux\Laura Borrel\Batch\LB-553 IB 12 0.8 mL.min 8µL t.lcd

Sample Name : LB-553 IB 12 0.8 mL.min 8µL
Vail # : 2
Injection Volume : 8 uL
Data File Name : LB-553 IB 12 0.8 mL.min 8µL t.lcd
Method File Name : I 88-12 40min voie 0 - 0.8 mLmin.lcm
Report File Name :
Data Acquired : 27/06/2025 11:10:29

C:\LabSolutions\Data\Projet 2\D1\David Virieux\Laura Borrel\Batch\LB-553 IB 12 0.8 mL.min 8µL t.lcd

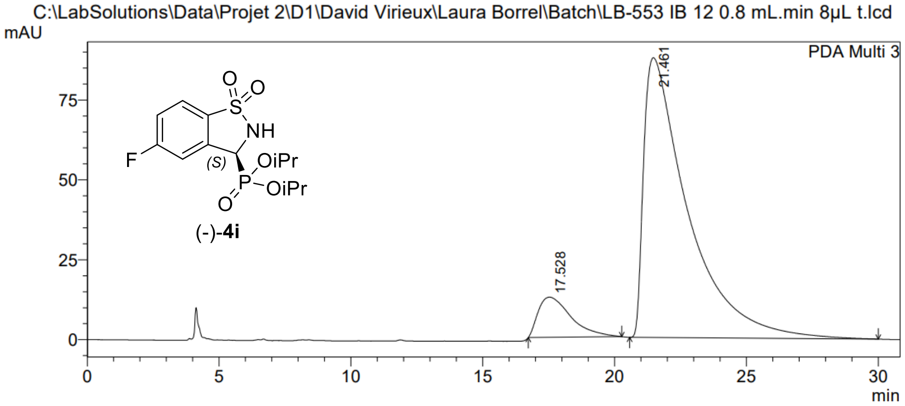

1 PDA Multi 3/220nm 4nm

PeakTable

PDA Ch3 220nm 4nm

Peak#	Ret. Time	Area	Height	Area %	Height %
1	17.528	1087201	12543	9.628	12.531
2	21.461	10205219	87550	90.372	87.469
Total		11292421	100093	100.000	100.000
